# Cryo-EM structures of Thogoto virus polymerase reveal unique RNA transcription and replication mechanisms among orthomyxoviruses

**DOI:** 10.1038/s41467-024-48848-3

**Published:** 2024-05-30

**Authors:** Lu Xue, Tiancai Chang, Zimu Li, Chenchen Wang, Heyu Zhao, Mei Li, Peng Tang, Xin Wen, Mengmeng Yu, Jiqin Wu, Xichen Bao, Xiaojun Wang, Peng Gong, Jun He, Xinwen Chen, Xiaoli Xiong

**Affiliations:** 1 State Key Laboratory of Respiratory Disease, Guangdong Provincial Key Laboratory of Stem Cell and Regenerative Medicine, Guangdong Provincial Key Laboratory of Biocomputing, GIBH-CUHK Joint Research Laboratory on Stem Cell and Regenerative Medicine, Guangzhou Institutes of Biomedicine and Health, Chinese Academy of Sciences, Guangzhou, China; 2 Guangzhou National Laboratory, Guangzhou, Guangdong China; 3 College of Medicine, Southern University of Science and Technology, Shenzhen, China; 4 Key Laboratory of Special Pathogens and Biosafety, Wuhan Institute of Virology, Center for Biosafety Mega-Science, Chinese Academy of Sciences, Wuhan, Hubei China; 5 University of Chinese Academy of Sciences, Beijing, China; 6 State Key Laboratory of Veterinary Biotechnology, Harbin Veterinary Research Institute, Chinese Academy of Agricultural Sciences, Harbin, China

**Keywords:** Cryoelectron microscopy, Enzyme mechanisms, Nucleic acids, Influenza virus, Virus-host interactions

## Abstract

Influenza viruses and thogotoviruses account for most recognized orthomyxoviruses. Thogotoviruses, exemplified by Thogoto virus (THOV), are capable of infecting humans using ticks as vectors. THOV transcribes mRNA without the extraneous 5′ end sequences derived from cap-snatching in influenza virus mRNA. Here, we report cryo-EM structures to characterize THOV polymerase RNA synthesis initiation and elongation. The structures demonstrate that THOV RNA transcription and replication are able to start with short dinucleotide primers and that the polymerase cap-snatching machinery is likely non-functional. Triggered by RNA synthesis, asymmetric THOV polymerase dimers can form without the involvement of host factors. We confirm that, distinctive from influenza viruses, THOV-polymerase RNA synthesis is weakly dependent of the host factors ANP32A/B/E in human cells. This study demonstrates varied mechanisms in RNA synthesis and host factor utilization among orthomyxoviruses, providing insights into the mechanisms behind thogotoviruses’ broad-infectivity range.

## Abstract (web summary)

In this study the authors present the Thogoto virus polymerase cryo-EM structures and reveal unique RNA synthesis mechanisms among orthomyxoviruses. RNA synthesis by Thogoto virus polymerase is shown to be weakly dependent on the host factors ANP32A/B/E in human cells in contrast to influenza viruses.

## Introduction

Orthomyxoviruses comprise the influenza virus genera including *Alphainfluenzavirus* (influenza A viruses, IAV), *Betainfluenzavirus* (influenza B viruses, IBV), *Gammainfluenzavirus* (influenza C viruses, ICV) and *Deltainfluenzavirus* (influenza D viruses, IDV). Besides the influenza viruses, which are important in both human and veterinary medicines, thogotoviruses form a prominent genus within the Orthomyxovirus family. Within the thogotovirus genus, Thogoto virus (THOV), Dhori virus (DHOV), Bourbon virus (BRBV), and Jos virus (JOSV) and a few others are currently recognized^1^. Thogotoviruses are different from influenza viruses in that they are arthropod-borne viruses with the ability to transmit to mammals using ticks as the vector^2,3^. THOV^4^, DHOV^5^, and BRBV^6^ have been reported to infect humans, causing diseases and sometimes deaths.

Orthomyxoviruses are segmented negative-sense RNA viruses (sNSV) and they are distinct from most other RNA viruses in that their genome is transcribed and replicated within the cell nucleus. Being an orthomyxovirus, THOV’s genome comprises six negative-sense single-stranded RNA segments^7^. Numbered by size, segments 1, 2 and 3 encode the PB2, PB1 and PA subunits respectively to constitute the THOV polymerase. Segment 4 and 5 encodes the glycoprotein^8^ and the nucleoprotein (NP)^9^. Finally, segment 6 encodes the matrix protein (M)^10–12^ and, through alternative splicing, ML, an interferon antagonist^13^. Similar to influenza virus, within the virion, PB2, PB1, PA and NP assemble with each viral genome segment to form a vRNP (viral ribonucleoprotein) complex. In the initial stage of orthomyxovirus infection, a productive life cycle is initiated when the vRNP complexes start viral mRNA transcription, after being imported into the nucleus. Interestingly, it has been reported that the mRNA transcribed by the THOV polymerase is distinctive from that of influenza viruses. THOV mRNA lacks extraneous heterologous sequences at the 5′ end^9,14^, in contrast to influenza mRNA, which carries heterologous 5′ sequences ranging from 9 to 13 nucleotides^15–18^. The 5′ heterologous sequences in influenza mRNA have been attributed to the sequences derived from host mRNA by the process of cap-snatching^17,19^. Perplexingly, THOV mRNA is found to have a cap structure^9,14^, despite that 5′ heterologous sequence is absent. The details of THOV mRNA transcription remain largely unclear.

The vRNP complex is also central to orthomyxovirus genome replication. In this process, the polymerase complex initiates de novo synthesis and reads through the vRNA to produce cRNA (complementary RNA)^20^. The cRNA can be further utilized to synthesize vRNA to complete the viral genome replication. Recent evidence has emerged showing that host factors, especially the acidic nuclear phosphoprotein 32 (ANP32) proteins, play an important role in influenza virus genome replication by interacting with the polymerase^21–30^. THOV is distantly related to influenza viruses; THOV genome replication remains largely uncharacterized.

Here, we report a series of THOV polymerase cryo-EM structures with resolutions from 2.3 to 3.2 Å to reveal the initiation and elongation processes of THOV RNA synthesis. The distinctive mechanisms revealed in relation to THOV RNA transcription, replication and host-factor utilization by comparison with influenza viruses provide new evidence that RNA synthesis mechanisms among orthomyxoviruses exhibit profound variability. We discuss such variability in relation to virus host-range.

## Results

### THOV polymerase transcription and replication activity

**Fig. 1:**
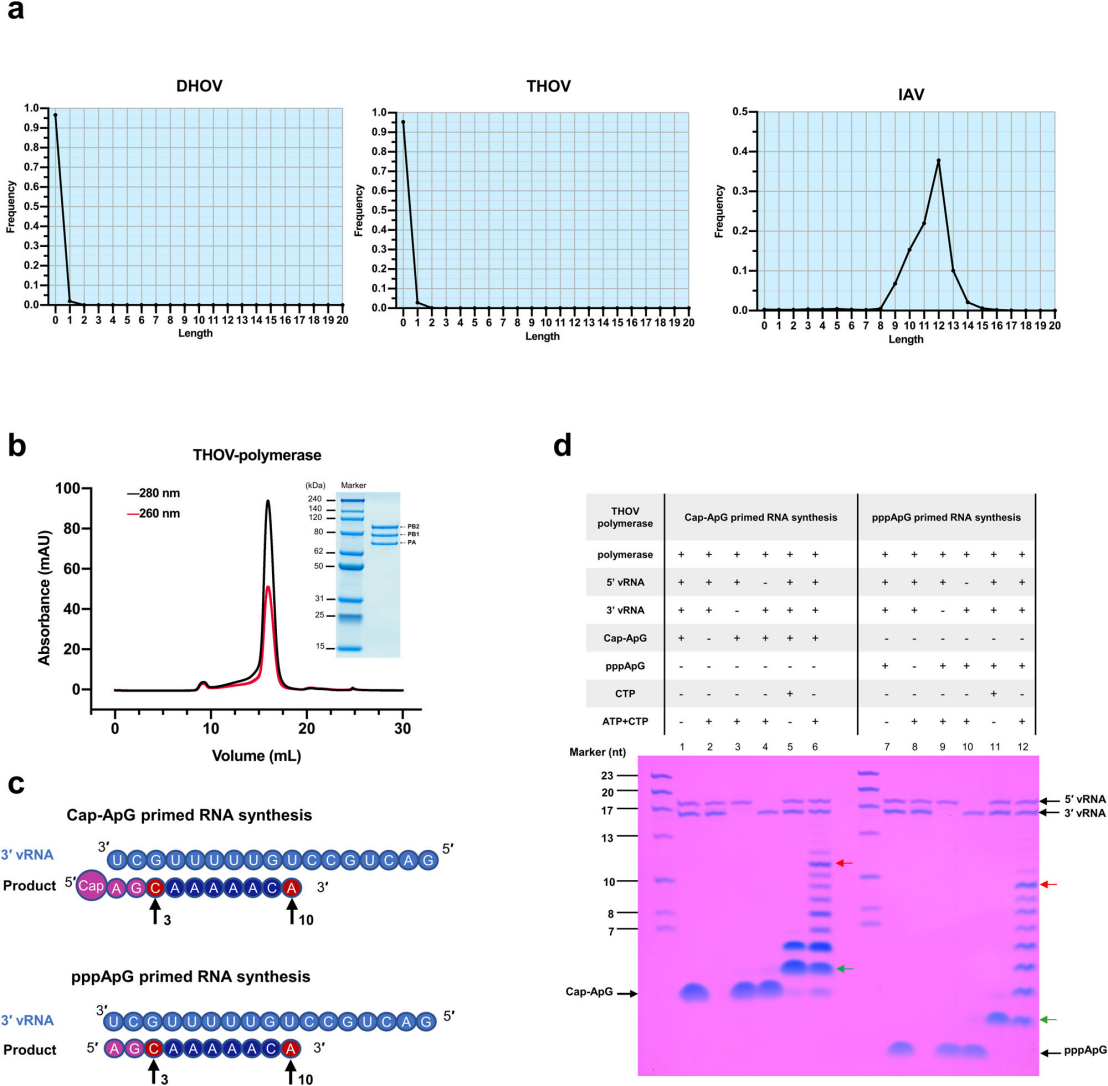
In vivo and in vitro RNA synthesis by THOV polymerase. **a** High-throughput sequencing analysis of the 5′ end regions of mRNA synthesized by mini-replicons of influenza A virus (IAV) and two thogotoviruses – Thogoto virus (THOV) and Dhori virus (DHOV). Histograms show the lengths and frequencies of non-templated (heterologous) sequences found in mRNA transcribed by DHOV, THOV and IAV mini-replicon systems. **b** Size-exclusion chromatography of THOV polymerase complex and the associated Coomassie-blue stained SDS-PAGE gel showing purified THOVPol containing three polymerase subunits. **c** Schematics showing sequences and features of 3′ vRNA template, capped (top panel) and uncapped primers (bottom panel). A representative result from at least 3 independent experiments is shown. **d** In vitro transcriptional and replicative primer extension assays confirming the RNA synthesis activities of the purified THOVPol. Capped (lane 1–6) and uncapped (lane 7–12) primers were tested. Capped/uncapped 3-nt expected elongation products are observed in the presence of CTP alone (lane 5 and 11, indicated by green arrows); Capped/uncapped 10-nt expected elongation products are observed when both ATP and CTP are present (lane 6 and 12, indicated by red arrows). The RNA products from the elongation reactions exhibit heterogeneity (extra bands in lane 5–6 and 11–12) likely due to premature termination (bands smaller than the expected products) and run-off products (bands larger than the expected products). A representative result from at least 3 independent experiments is shown. Source Data are provided as a Source Data file.

To study THOV polymerase (THOVPol) RNA synthesis, we constructed mini-replicon systems expressing green fluorescent protein (GFP) driven by thogotovirus polymerases – THOV and DHOV (Dhori virus^31^) polymerases. A mini-replicon driven by an influenza A virus (IAV) polymerase (FluAPol) was also studied for comparison (Fig. 1a**;** Supplementary Fig. 1). We used high-throughput sequencing to profile the 5′ ends of GFP RNA transcripts. The sequencing results show that the 5′ end sequences of RNA transcripts by polymerases of THOV or DHOV are devoid of extraneous heterologous sequence. By contrast, RNA transcripts by the FluAPol comprise heterologous sequences of 9–14 nucleotides (Fig. 1a). These results are in accordance with previous reports on 5′ sequences of THOV^9,14^ and IAV^18^ mRNA transcripts.

To further explore the mechanism of RNA synthesis by THOVPol, we produced full-length heterotrimeric THOVPol in insect cells (Fig. 1b). The purified THOVPol complex demonstrates RdRp (RNA dependent RNA polymerase) activities in RNA template-dependent primer-extension assays. Using a 17-mer 3′ vRNA as the template (Fig. 1c) and in the presence of a 18-mer 5′ vRNA, as well as ATP and CTP, the polymerase demonstrates the ability to incorporate nucleotides into the capped (m7GpppAmG) or uncapped (pppApG) 2-mer primers to form the expected products (Fig. 1d, lane 6 and 12). In limited RNA synthesis reactions in the presence of CTP alone, we also observed the expected products, which are 1-mer longer than the primers (Fig. 1d, lane 5 and 11). Further assays confirmed that the conserved sequences located in the 5′ and 3′ untranslated regions of the THOV RNA genome are essential in initiating THOV polymerase RNA synthesis in vitro (Fig. 1d, lane 3, 4, 9, 10).

### THOVPol in transcription pre-initiation conformations

**Fig. 2:**
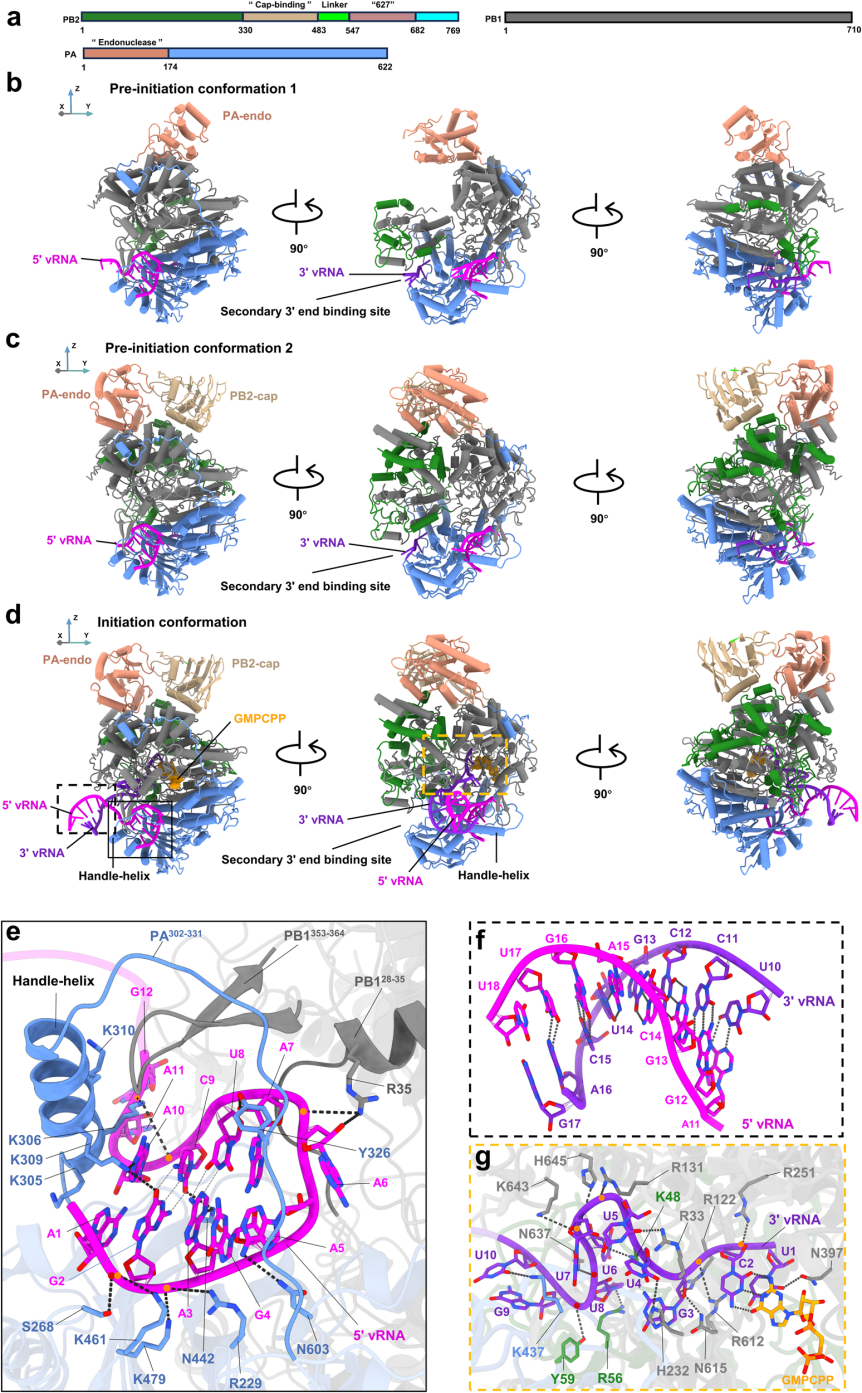
Structures of THOVPol in transcription pre-initiation and initiation conformations. **a** Schematic representation of the domain organizations in THOVPol subunits. The color scheme for each component of the polymerase complex is used in **b**–**d**. **b**–**d** Three different views of THOVPol structures in pre-initiation and initiation conformations. 5′ vRNAs are colored magenta; 3′ vRNAs are colored violet; and GMPCPP is colored orange. Boxed areas in **d** indicate magnified features shown in **e**–**g**. **e** Details of 5′ vRNA binding within the 5′ promoter binding site. Residues interacting with RNA are shown and labeled, black dashed lines indicate hydrogen bonds and electrostatic interactions. Conventional basepairs within the 5′ vRNA are indicated by hydrogen bonds between bases. **f** Sequence complementarity between nucleotides 11–17 of the 5′ vRNA and nucleotides 10–16 of the 3′ vRNA in the RNA duplex formed in the initiation conformation. Hydrogen bonds between conventional basepairs are indicated by dash lines. **g** Close-up views of nucleotides 1–10 of 3′ vRNA and their interaction with polymerase active site residues and GMPCPP. Residues interacting with RNA are shown and labeled, black dashed lines indicate hydrogen bonds and electrostatic interactions.

Three featured conformations relating to transcription initiation were observed by cryo-EM using a sample containing THOVPol, 5′ vRNA, 3′ vRNA, m7GpppA and GMPCPP, a nonhydrolyzable GTP analog (Fig. 2a–d, Supplementary Fig. 2 and Supplementary Table 3). The first identified structure, designated “pre-initiation conformation 1”, shows a polymerase heterotrimer formed by PB2, PB1 and PA (referred to as a polymerase complex monomer, or simply a polymerase) (Fig. 2b, Supplementary Fig. 2 and Supplementary Table 3). Although the 3 polymerase subunits show low sequence identities to their influenza counterparts (∼15%, ∼27% and ∼19% to IAV PB2, PB1 and PA, respectively), THOVPol exhibits similarity in overall architecture to influenza virus polymerases (FluPols). PB1 forms the THOV polymerase core, which is surrounded by PB2 and PA. In pre-initiation conformation 1, the majority of the PA putative endonuclease domain is visible. There is no density for the PB2 putative cap-binding domain and the 627 domain^28,32^, likely indicating them being flexible. The 5′ vRNA is found binding to the 5′ promoter binding site, which is formed by PA and PB1 subunits^27,33–36^ (Fig. 2b, left panel). The 3′ vRNA binds within the secondary 3′ end binding site groove formed by the C-terminal domain of PA and the PB1 subunit (Fig. 2b, middle panel); this site has been proposed to bind 3′ vRNA prior to its entry into the polymerase active site cavity^34^. Due to that the 3′ vRNA is yet to enter the polymerase active site cavity, we ascribe this structure as a pre-initiation conformation.

We further determined THOVPol structure in “pre-initiation conformation 2” (Fig. 2c and Supplementary Fig. 2), in which, the 5′ and 3′ vRNAs are maintained at their respective binding sites as in the pre-initiation conformation 1. Distinctively, the PA putative endonuclease domain rotates by ∼157° and translates by ∼29 Å from its position in the pre-initiation conformation 1 (Supplementary Fig. 3a). Additionally, the PB2 putative cap-binding domain is now visible. More PB2, PB1 and PA residues are resolved likely due to changes in the structure and configuration of the peripheral domains (compare Fig. 2b, c middle panels). In FluPols, two peripheral domains, PB2 cap-binding and PA endonuclease domains, carry out the “cap-snatching” activity. The PB2 cap-binding domain binds the 5′ cap of mRNA, and the PA endonuclease domain cuts the bound mRNA to generate 9- to 13-nucleotide capped RNA fragments to serve as primers for viral mRNA synthesis. The length of primers accounts for the spatial distance between the two cap-snatching domains within the polymerase complex^35,37,38^.

A crystallographic study of isolated THOVPol putative cap-snatching domains revealed notable divergence in key residues within the putative endonuclease and cap-binding sites, compared to those in FluPols. Such results hint at cap-snatching being non-functional in THOVPol^39^. Indeed, no biochemical activity was detected for the isolated THOVPol putative cap-snatching domains^39^. Comparisons identify that the structures of the two putative cap-snatching domains within our cryo-EM structures are essentially the same as the reported crystal structures^39^ (Supplementary Fig. 3d).

### THOVPol in a transcription initiation conformation

The third featured structure is identified as an “initiation conformation” (Fig. 2d, Supplementary Fig. 2 and Supplementary Table 3), in which, nucleotides 1-8 from the 3′ end of the 3′ vRNA enter into the polymerase active site cavity (Fig. 2d, left and middle panels; Fig. 2g). The peripheral domains retain their positions as observed in the pre-initiation conformation 2. Entry of the 3′ vRNA into the polymerase active site is facilitated by nucleotides 10–17 of the 3′ vRNA forming an RNA duplex with 5′ vRNA nucleotides 11-18 outside of the polymerase active site cavity (compare Fig. 2c, d, middle panels; Fig. 2f). Within the RNA duplex, strict sequence complementarity is found between 5′ vRNA nucleotides 11-17 and 3′ vRNA nucleotides 10–16 (Fig. 2f). By sequence analysis, such complementarity is found in each THOV vRNA, except for segment 4, in which a single mismatch is found^40^.

The entry of 3′ vRNA 3′ end sequence 5′-UUUUUGCU-3′ into the polymerase active site cavity (Fig. 2g), triggers reorganization of structural motifs within the polymerase core and rigidifying multiple loops, including PB2^56-126^ and PB1^476-635^ (containing the priming loop) motifs (Supplementary Fig. 4a, b) Multiple pairs of hydrogen bonds and electrostatic interactions are formed once the 3′ vRNA is bound in the active site (Fig. 2g). Additionally, a GMPCPP molecule is base-paired with C2 at the penultimate position from the 3′ end of the 3′ vRNA within the polymerase active site (Fig. 2d, left panel; Fig. 2g). We did not observe the added capped m7GpppA in the polymerase active site. The above-described features suggest that this structure likely mimics a transcription initiation conformation. Previously, influenza virus PB2 cap-binding domains were shown to bind m7G^41–44^. Notably, no m7GpppA density is observed in the THOV PB2 putative cap-binding domain, consistent with the proposal that the putative cap-binding domain is inactive^39^.

In the pre-initiation and initiation conformations (as exemplified in Fig. 2e), the 5′ promoter binding site adopts a cradle-like structure similar to those in FluPols^27,33,34,37^. The base of the cradle is primarily located within the PA, while a short stretch of PB1 N-terminal sequence (PB1^28-35^) also forms part of the cradle. A handle is formed above the cradle by residues PA^302-331^. The handle structure is stabilized by interactions with a β-hairpin from PB1 (PB1^353-364^) (Fig. 2e; Supplementary Fig. 5a). A distinct helix structure is formed towards the N-terminal side of the cradle handle, and we name it the “handle-helix” (PA^305-316^) (Fig. 2e). To bind the cradle, nucleotides 1–10 of the 5′ vRNA adopt a hook structure. The hook structure is stabilized by two conventional G2-C9 and A3-U8 basepairs. The hook also contains two mismatched A1-A10 and G4-A7 basepairs. The A5 and A6 bases in the middle of the hook are flipped out (Fig. 2e). From the cradle, R229^PA^, S268^PA^, Y326^PA^, N442^PA^, K461^PA^, K479^PA^, N603^PA^ and R35^PB1^ form specific interactions with the bound 5′ vRNA promoter (Fig. 2e). The interactions formed by the above residues appear to be specific for THOVPol, and the residues involved are not strictly conserved among the polymerases of thogotoviruses (Supplementary Fig. 6a, b).

The handle-helix contains 4 strategically-positioned positively charged residues, K305^PA^, K306^PA^, K309^PA^ and K310^PA^, which engage extensive interactions with the 5′ and 3′ ends of the bound 5′ vRNA hook (Fig. 2e). K305^PA^ engages in a cation-π interaction with the base of A1; K306^PA^ is located near the 3′ end of the bound 5′ vRNA hook and forms salt bridges with the backbone phosphates of A10 and G12. K309^PA^ extends into the interior of the bound 5′ vRNA to form hydrogen bonds with G2 and A10 bases. Although no specific RNA interaction is engaged by K310^PA^, its close proximity to the phosphate backbone of the bound 5′ vRNA likely stabilizes RNA binding through long-range electrostatic interactions. K305^PA^, K306^PA^ and K309^PA^ are conserved among thogotoviruses (see below).

### THOVPol adopts monomeric and dimeric conformations in transcriptional RNA synthesis

**Fig. 3:**
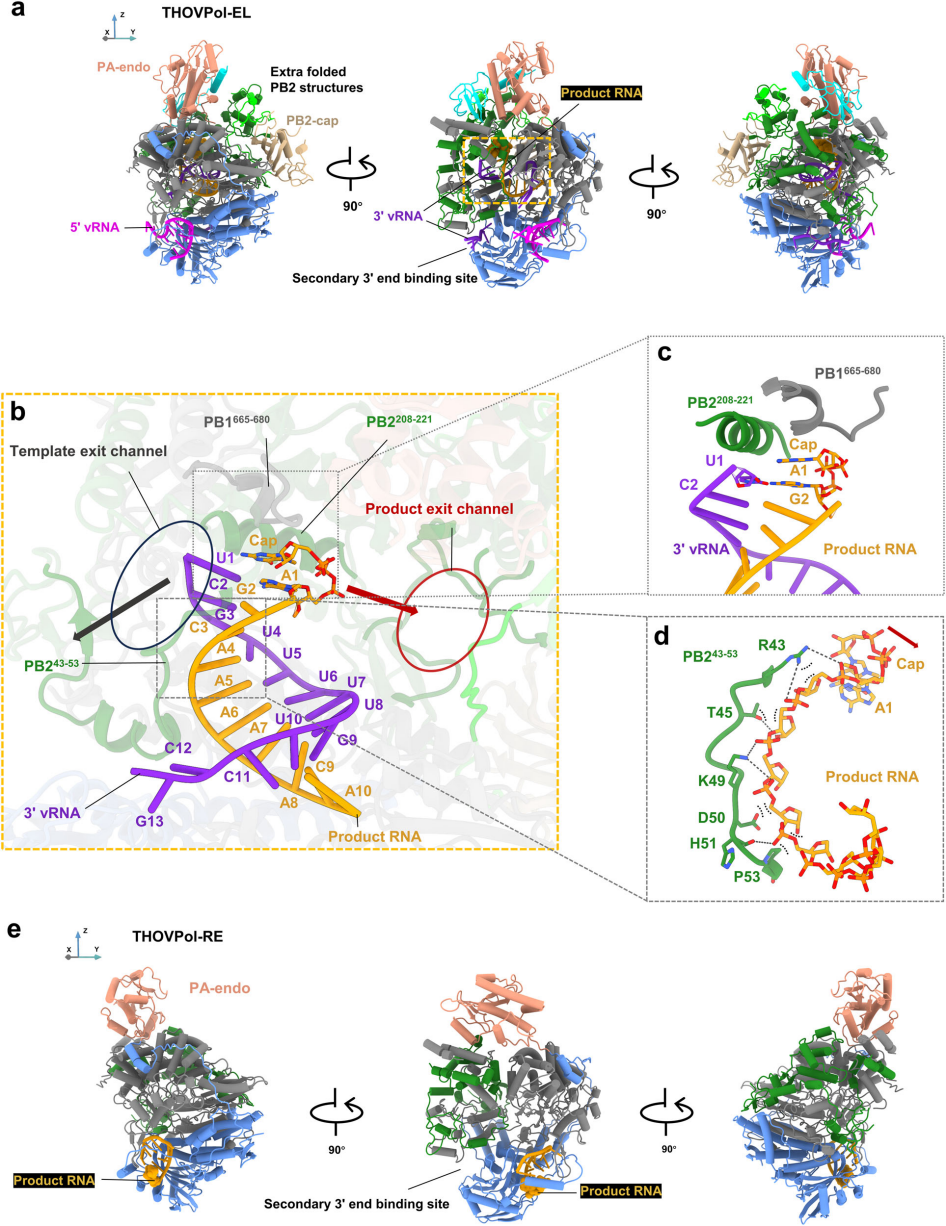
Structures of the monomeric THOV polymerase complexes formed during transcriptional RNA synthesis. **a** Three different views of elongation THOVPol (THOVPol-EL) in cartoon representation. **b** The 10-basepair RNA duplex formed between RNA product and 3′ vRNA in polymerase active site cavity. The template exit channel in THOVPol-EL is circled and the trajectory of template exit is indicated by a black arrow; the product exit channel in THOVPol-EL is circled and a red arrow marks the product RNA exit trajectory. **c** Two structural motifs, PB2^208-221^ and PB1^665-680^, are found to stack on the template nucleotide U1 and the 5′ cap-nucleotide. **d** The stretched PB2^43-53^ loop allows interactions with the phosphate-ribose backbone of the product RNA. Dash lines indicate hydrogen bonds and electrostatic interactions. The VDW contacts are represented as black dashed curves. **e** Three different views of the receiving THOVPol (THOVPol-RE) in cartoon representation. The product RNA is colored orange, and the cap-1 structure (m7GpppAm) is shown as spheres.

**Fig. 4:**
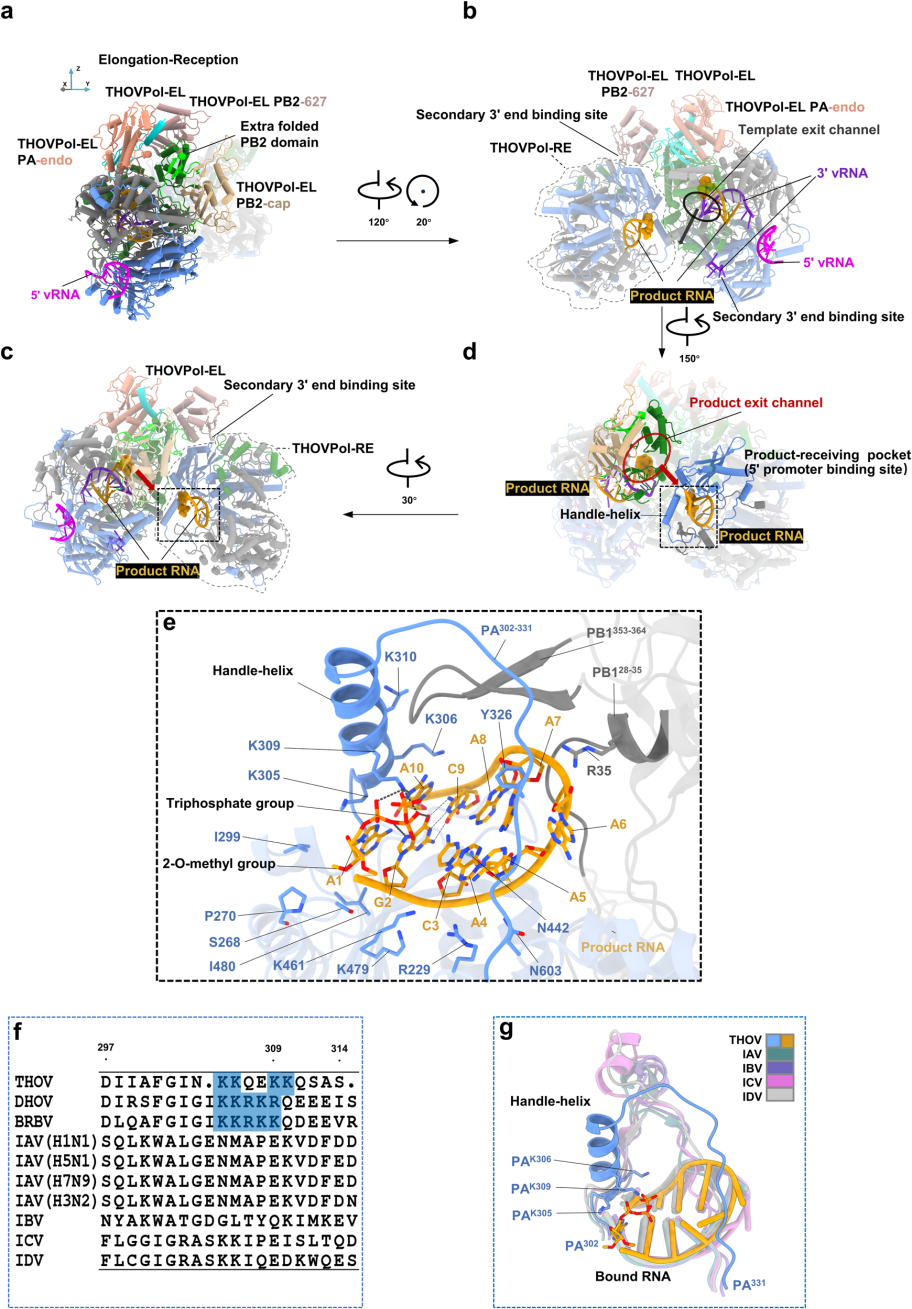
Formation of an asymmetric THOVPol dimer during transcriptional RNA synthesis. **a**–**d** Four different views of the asymmetric THOVPol dimer formed during transcriptional RNA synthesis. The product RNA is colored orange, and the cap-1 structure (m7GpppAm) is shown as spheres. The template exit channel in THOVPol-EL is circled and the template exit trajectory is indicated with a black arrow. The product exit channel in THOVPol-EL is circled and the product exit trajectory is indicated with a red arrow. **e** Product RNA (orange) bound to the product-receiving pocket (5′ promoter binding site) formed by PA and PB1. Cap-1 structures comprising the triphosphate group and the 2-O-methyl group at nucleotide A1 located at the 5′ end of the product RNA are indicated and shown as sticks. Sidechains of the RNA interacting residues from the product-receiving pocket are shown as sticks; they form similar interactions as observed for 5′ vRNA binding (see Fig. 2e); hydrogen bonds and salt-bridges formed by them are omitted for clarity. The black dashed lines indicate the interactions from K309^PA^ of the handle-helix to the triphosphate group and bases (G2 and A10). The conventional basepairs within the bound product RNA are shown as thin gray dashed lines. Note that the triphosphate group engages in intra-molecular interaction forming 2 charged hydrogen bonds with G2 guanine of product RNA. **f** Multiple sequence alignment of the PA handle-helix regions of different orthomyxoviruses. The highlighted (blue) residues, K305^PA^, K306^PA^ and K309^PA^, are conserved in the thogotovirus genus. **g** Comparison of the 5′ promoter binding sites and the bound RNA molecules in polymerases of THOV and influenza viruses (PDB: 6RR7 (IAV), 4WRT (IBV), 6XZG (ICV), 6KUU (IDV)).

To further understand THOVPol RNA transcription, we prepared a transcriptional elongation sample by primer-extension of m7GpppAmG, a dinucleotide primer with a cap-1 structure. Cryo-EM imaging of this sample identified THOVPol in two monomeric conformations and one asymmetric dimer conformation (Fig. 3a, e; Fig. 4a–d and Supplementary Fig. 7; Supplementary Table 4). Product RNA is only identified within the polymerase active site in one monomeric conformation, which is designated “THOVPol-EL”, representing a transcriptional elongation conformation.

In THOVPol-EL, the RNA duplex formed between the 5′ and 3′ vRNAs in the initiation conformation is completely disrupted. The template 3′ vRNA is further translocated by 9 nt into the polymerase active site cavity (Fig. 3a, middle panel and Fig. 3b). Translocation of the template is likely caused by the elongation process, as evidenced by the 10-nt product RNA bound in the polymerase active site cavity (Fig. 3b). The product RNA represents the elongation of the m7GpppAmG primer by 8 nucleotides. The RNA product forms a 10-basepair duplex with the 3′ vRNA (Fig. 3b). An additional 3′ vRNA is bound in the secondary 3′ end binding site of THOVPol-EL (Fig. 3a, middle panel), similar to the pre-initiation conformations (see Fig. 2b, c). Re-configuration of both the putative cap-snatching domains is observed (compare Supplementary Fig. 8a, b).

The other monomeric conformation contains no RNA in the polymerase active site cavity. Only a single RNA segment is bound in the 5′ promoter binding site (Fig. 3e, left and middle panels). This RNA shows density for 10 nucleotides with a triphosphate group from the 5′ cap, identifying this segment as the product RNA (Supplementary Fig. 7 and Fig. 3e). Therefore, the product RNA is likely received after release from the transcribing THOVPol-EL. We designate the second monomeric conformation as a product receiving “THOVPol-RE” conformation.

**Fig. 5:**
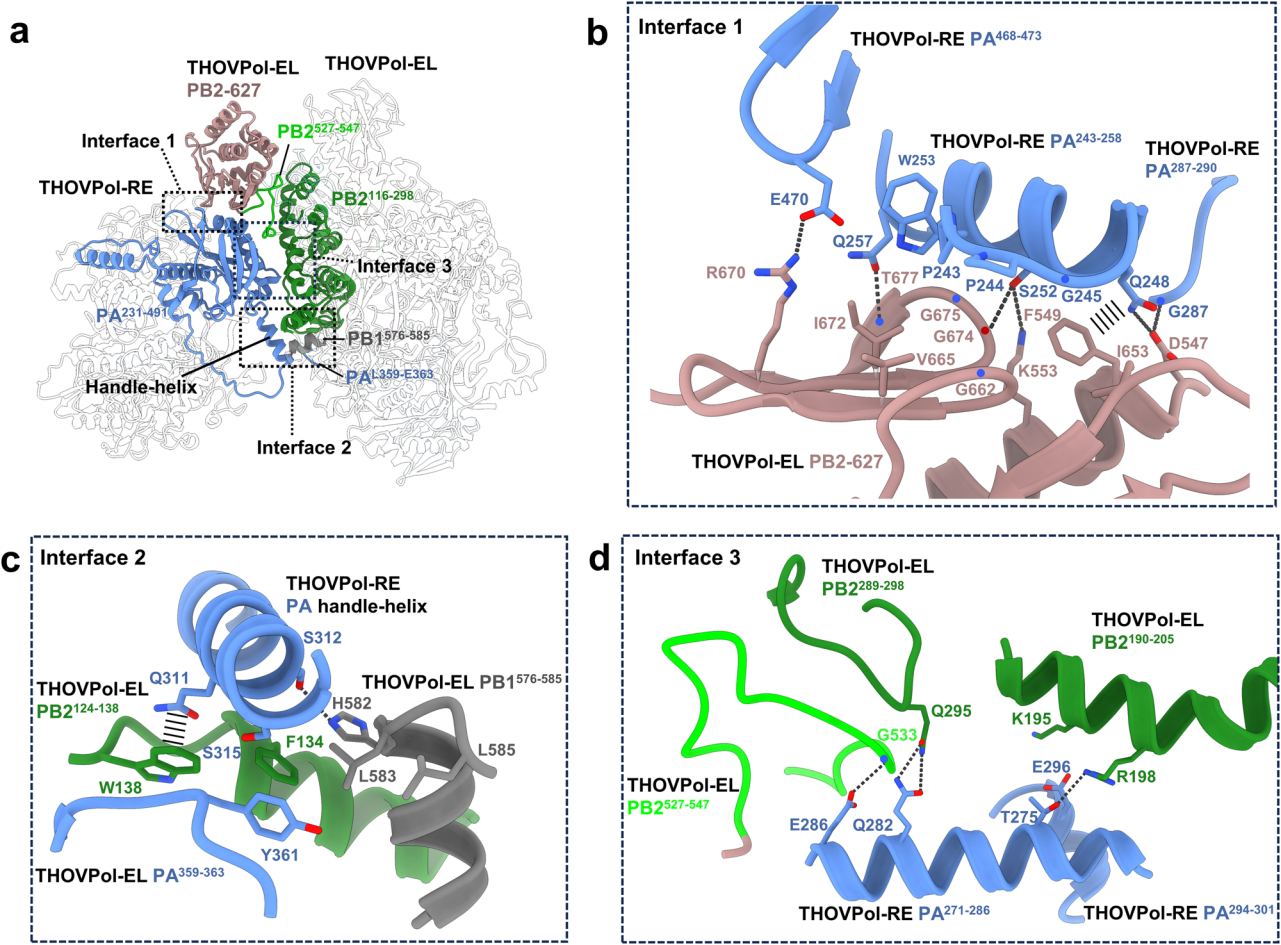
The interfaces of the asymmetric THOVPol dimer. **a** The asymmetric THOVPol dimer is shown with regions involved in dimer formation highlighted in colors. The locations of the three interaction interfaces primarily between the PA of THOVPol-RE and the PB2 of THOVPol-EL are shown by the dashed boxes and their structures are detailed in **b–d**. **b** Interactions in interface 1 between THOVPol-RE PB2-627 domain (salmon) and THOVPol-RE PA structural elements (blue). Hydrogen bonds and salt-bridges are shown by dashed lines, amide-π interactions are indicated by wide dashed lines. **c** Interactions in interface 2 between THOVPol-RE PA handle-helix (blue) and THOVPol-EL PB2 (green), PB1 (gray) and PA (blue) structural elements. **d** Interface 3 interactions between THOVPol-RE PA (blue) and THOVPol-EL PB2 structural elements (green and lime).

The asymmetric dimer is essentially formed by the association of THOVPol-EL and THOVPol-RE, representing an “elongation-reception” conformation (Fig. 4a–d and Supplementary Fig. 7, Supplementary Table 4). Previously, asymmetric polymerase dimer undergoing RNA synthesis has not been captured for orthomyxoviruses. Influenza C virus (ICV) polymerase (FluCPol) asymmetric dimers were captured without product RNA bound^27^. THOVPol-EL and THOVPol-RE in the asymmetric dimer differ from their monomeric forms by configuration differences in their peripheral domains (compare Supplementary Fig. 8b–e). PB2 627 domain is resolved in THOVPol-EL within the asymmetric dimer (Fig. 4a–c), but is not visible in the monomeric form of THOVPol-EL (Fig. 3a). The ordering of the PB2 627 domain likely facilitates the recruitment of a THOVPol-RE molecule to form the asymmetric dimer (see Fig. 5). The PA putative endonuclease domain is resolved in the monomeric form of THOVPol-RE (Fig. 3e) but invisible in THOVPol-RE of an asymmetric dimer (Fig. 4b, c). Apart from the above discussed details, THOVPol-EL exhibits almost identical features whether in its monomeric form or as part of an asymmetric dimer. Similarly, THOVPol-RE maintains nearly identical features across its monomeric and asymmetric dimer forms.

### Structural features of THOVPol-EL

By comparison with the initiation conformation, the formation of the template-product RNA duplex in the THOVPol-EL polymerase active site cavity (Fig. 3a, b) promotes the polymerase core to adopt a more open conformation (Supplementary Fig. 4c). Specifically, the template exit channel is unblocked (Supplementary Fig. 4d), and the THOVPol-EL priming loop is fully extruded (Supplementary Fig. 4e). We observed notable structural changes in two polymerase core structural motifs, PB2^208-221^ and PB1^665-680^, involved in opening the product exit channel (Fig. 3b, c; Supplementary Fig. 4f). These two motifs are found to stack on the nucleotides of template U1 and 5′ cap (Fig. 3c). While these motifs may stabilize the RNA duplex, they may also facilitate the subsequent template-product strand separation (Fig. 3b, c). We also found that the PB2^43-53^ loop (Fig. 3b, d) is stretched by the opening of the polymerase core (Supplementary Fig. 4g), to interact with the phosphate-ribose backbone of the RNA product via hydrogen bonds, electrostatic interactions and VDW (Van der Waals) contacts (Fig. 3d). These interactions may support sustained sliding of the RNA product and its passage into the product exit channel. The two structural motifs, PB1^665-680^, gating the product exit channel, and PB2^43-53^ loop, interacting with the bent product RNA on the opposite side of the product exit channel, appear to function in concert to guide the capped RNA product into the product exit channel (Fig. 3b). Interestingly, the interactions mediated by the PB1^665-680^ and PB2^43-53^ motifs appear to be specific to THOVPol; equivalent interactions have not been found in FluPol structures bound to RNA products. We note that within the asymmetric dimer, the opening of the product exit channel is directly opposite to the 5′ promoter binding site of THOVPol-RE (Fig. 4c, d).

Within the polymerase active site cavity, the RNA elongation causes an upward movement of the PB1^665-680^ motif (Supplementary Fig. 4f). The moved PB1^665-680^ motif would clash with the PA putative endonuclease domain if this domain were positioned as observed in the initiation conformation (Supplementary Fig. 3e). Therefore, RNA elongation triggers the re-configuration of polymerase peripheral domains in THOVPol-EL. From their positions in the initiation conformation, the PA putative endonuclease domain rotates by ∼122° and translates by ∼19 Å (compare Supplementary Fig. 8a, b**;** Supplementary Fig. 3b); the PB2 putative cap-binding domain rotates by ∼158° and translates by ∼42 Å (compare Supplementary Fig. 8a, b; Supplementary Fig. 3c). Likely due to the peripheral domain re-configuration, more PB2 residues (PB2^264-329^; PB2^485-546^; PB2^686-751^) are resolved. Notably, PB2^264-329^ and PB2^485-546^ form extra structures between the putative PB2 cap-binding and PA endonuclease domains (Fig. 3a and Fig. 4a; Supplementary Fig. 8b, d).

### Structural features of THOVPol-RE

Within the asymmetric dimer, THOVPol-RE peripheral domains are likely to be flexible showing no density (Fig. 4b, c). The product RNA bound in the THOVPol-RE 5′ promoter binding site adopts a hook structure (Fig. 4e) similar to the 5′ vRNA in the THOVPol initiation conformation. The bound product RNA differs from the 5′ vRNA at nucleotides 3, 4, and 8 (compare Fig. 2e and Fig. 4e); There are 1 conventional basepair (G2-C9) and 3 mismatched basepairs (A1-A10, C3-A8 and A4-A7) within the hook (Fig. 4e). The cap-1 2-O-methyl group located on A1 of the product RNA appears to be well accommodated by the P270^PA^, I299^PA^ and I480^PA^ sidechains via hydrophobic contacts (Fig. 4e). Of these, P270^PA^ and I480^PA^ are conserved among PAs of thogotoviruses (Supplementary Fig. 6a). Density is not observed for the product RNA m7G cap, likely due to flexibility. However, the product RNA shows unambiguous densities for the cap triphosphate group (Supplementary Fig. 7d). The 4 strategically-positioned basic residues, K305^PA^, K306^PA^, K309^PA^ and K310^PA^, on the handle-helix, are located in close proximity to interact with the triphosphate group electrostatically. In particular, K309^PA^ forms 2 specific interactions to A10 and the triphosphate of the bound product RNA (Fig. 4e).

A sequence alignment shows that the PAs of THOV, DHOV and BRBV (Bourbon virus^6^) all contain 4-5 basic amino acids in the handle-helix region (Fig. 4f). Among them, residues equivalent to K305^PA^, K306^PA^ and K309^PA^ in THOVPol are strictly conserved among thogotoviruses (Fig. 4f). Based on the THOVPol structure, the strictly conserved K305^PA^, K306^PA^ and K309^PA^ emanate from the handle-helix towards the triphosphate group of the product RNA (Fig. 4e, g). Comparison reveals that the THOVPol PA handle-helix adopts a distinct structure compared with the equivalent regions in FluPols (Fig. 4g and Supplementary Fig. 5). The equivalent regions in FluPols form loop structures and are substantially away from the bound RNA molecules^27,34,35,38^ (Fig. 4g and Supplementary Fig. 5). The comparison suggests a unique interaction pattern between the THOVPol PA handle-helix and the 5′ triphosphate group of the RNA bound to the 5′ promoter binding site. Such interaction appears to be specific to the polymerases of thogotoviruses (Fig. 4f, g and Supplementary Fig. 5).

### Formation of asymmetric THOVPol dimer during replicative RNA synthesis

A replicative elongation sample was prepared using the non-capped dinucleotide primer pppApG. Cryo-EM imaging of the sample identified an asymmetric polymerase dimer structure (THOVPol-EL_R_-THOVPol-RE_R_) and a monomeric product receiving polymerase structure (THOVPol-RE_R_). No monomeric elongation polymerase structure (THOVPol-EL_R_) was observed (Supplementary Fig. 9, Supplementary Table 5). The asymmetric dimer structure determined from the replicative elongation sample closely resembles that determined from the transcriptional elongation sample (Supplementary Fig. 10a–d). THOVPol-EL_R_ and THOVPol-RE_R_ within the asymmetric dimer show high similarity in peripheral domain configuration (Supplementary Fig. 10a–d) and RNA binding (Supplementary Fig. 10a–i), compared with equivalent structures obtained from the transcriptional elongation sample. Therefore, we show that, starting from capped or non-capped dinucleotide, RNA synthesis induces highly similar THOVPol conformations.

### Interface of the asymmetric THOVPol dimer

We describe the dimer interface based on the THOVPol-EL-THOVPol-RE structure. The narrow but long dimer interface is formed by the THOVPol-EL PB2 subunit and the THOVPol-RE PA N-terminal domain (Fig. 5a). In THOVPol-EL, the interface spans multiple regions of PB2, including the PB2 627 domain (Fig. 5a).

Located on one end of the dimer interface (interface 1, Fig. 5b), the THOVPol-EL 627 domain exposes a hydrophobic surface containing F549^PB2^, I653^PB2^, G662^PB2^, V665^PB2^, I672^PB2^, G674^PB2^, and G675^PB2^. This surface contacts W253^PA^ and the hydrophobic PA^243-245^ loop (with the sequence “PPG”) of THOVPol-RE. The hydrophobic contact is further stabilized by salt bridges and hydrogen bonds (Fig. 5b). Notably, an amide-π interaction is found between F549^PB2^ and Q248^PA^ (Fig. 5b).

Towards the other end of the dimer interface (interface 2, Fig. 5c), interactions are formed across the THOVPol-RE PA handle-helix and structural elements from all 3 subunits of THOVPol-EL, namely, the PB2^124-138^ helix, PA^359-363^ loop and PB1^576-585^ helix. On THOVPol-EL, F134^PB2^, W138^PB2^, L583^PB1^, L585^PB1^ and Y361^PA^, from the 3 different subunits, form a hydrophobic patch to contact the THOVPol-RE PA handle-helix. This contact is further stabilized by an amide-π interaction between THOVPol-RE Q311^PA^ and THOVPol-EL W138^PB2^ and a hydrogen bond between THOVPol-RE S312^PA^ and THOVPol-EL H582^PB1^ (Fig. 5c). By comparison with the monomeric THOVPol-EL structure, reorientation of the W138^PB2^ sidechain is observed upon dimer formation (Supplementary Fig. 11).

In the middle of the dimer interface (interface 3, Fig. 5d), the THOVPol-RE PA^271-286^ helix interacts with several structural elements of THOVPol-EL PB2. Within the THOVPol-RE PA^271-286^ helix, the centrally located Q282^PA^ engages a pair of hydrogen bonds with Q295^PB2^ of the THOVPol-EL PB2^289-298^ loop (Fig. 5d). This central interaction is further stabilized by several hydrogen bond and electrostatic interactions that are located at or near both ends of the THOVPol-RE PA^271-286^ helix (Fig. 5d).

### Asymmetric THOVPol dimer and RNA synthesis

**Fig. 6:**
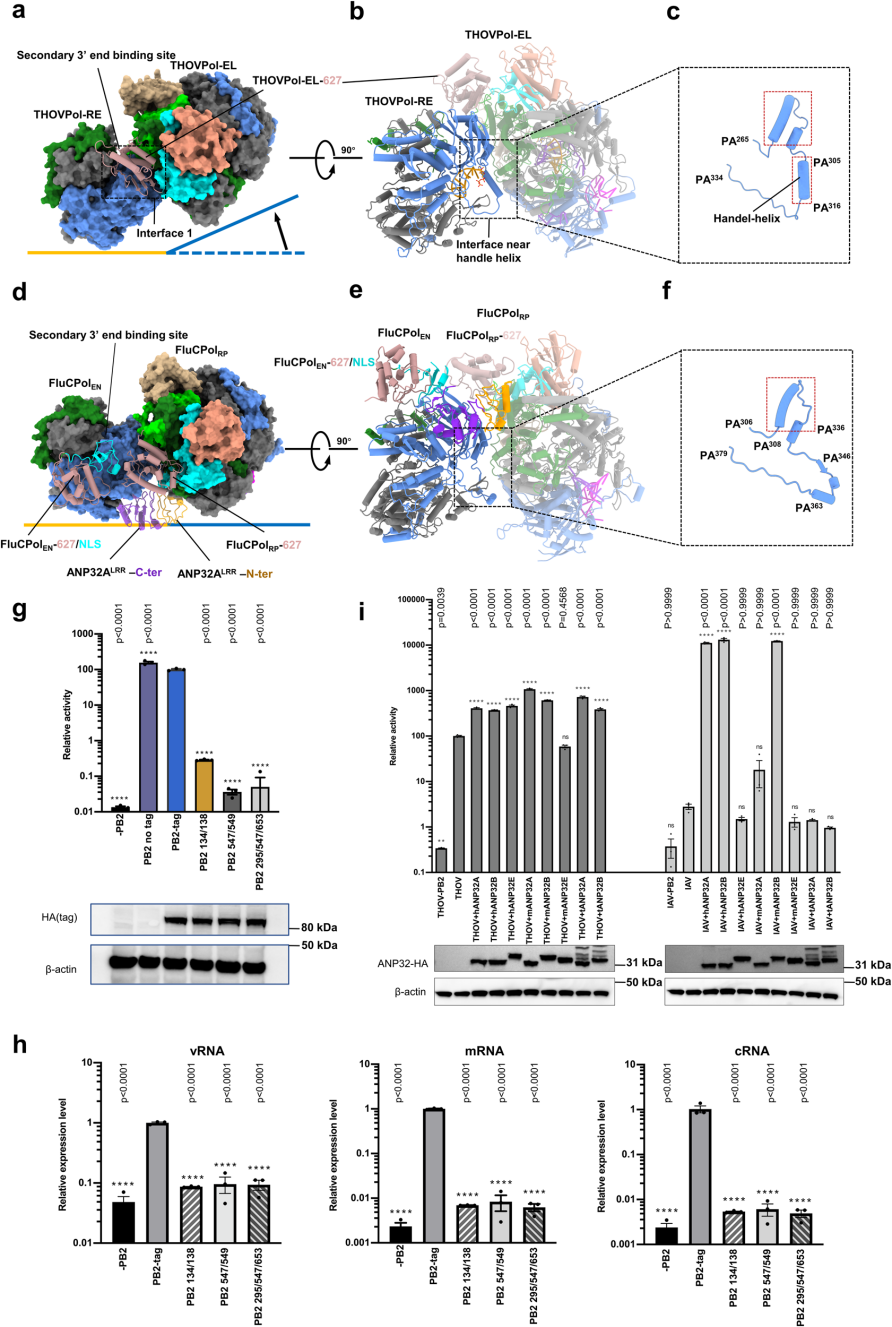
Asymmetric dimer structures of THOVPol and FluCPol compared. Up-and-down comparison of THOVPol (**a**, **b**) with FluCPol (**d**, **e**) (PDB 6XZQ) asymmetric dimer structures. Note that ANP32A binds at the FluCPol dimer interface. The THOVPol and FluCPol are aligned based on a structural alignment between THOVPol-RE and FluCPol_EN_. In **a** and **d**, Polymerase dimers are shown as topview surface representations; In **b** and **e**, Polymerase dimers are shown in cartoon sideviews. Location of the THOVPol-EL PB2-627 domain is indicated in **a** and **b**. The location of interface 1 involving the THOVPol-EL PB2-627 domain is shown in the dashed box in **a**; the configuration of the peripheral domains near the dimer interface 1 in the THOVPol dimer (**a**) is noticeably different from that in the FluCPol dimer (**d**). **c** The structural elements near the THOVPol-RE PA handle-helix are magnified and the structural elements involved in dimer formation are highlighted in dashed red-boxes. **f** The equivalent FluCPol_EN_ regions near the dimer interface are magnified and structural elements involved in dimer formation are highlighted in the dashed red-box. **g** Effects of dimer interface mutations on THOVPol activity as measured by a mini-replicon driven luciferase assay. **h** Effects of dimer interface mutations on levels of vRNA, mRNA and cRNA in a mini-replicon assay as measured by RT-qPCR. **i** In a human ANP32A (hANP32A), ANP32B (hANP32B) and ANP32E (hANP32E) triple knock-out (TKO) 293 T cell line, activities of THOVPol and FluAPol were assessed by a mini-replicon driven luciferase assay; effects of various human (h), mouse (m) and tick (t) ANP32 protein (hANP32A, hANP32B, hANP32E, mANP32A, mANP32B, mANP32E, tANP32A and tANP32B) expression in the TKO cells on THOVPol (THOV) and FluAPol (IAV) activities were tested. Activity and expression level data are reported as mean ± SEM from three independent experiments (*n* = 3). All statistics used one-way ANOVA Dunnett’s multiple comparisons test. (ns, *p* > 0.05; *p < 0.05; **p < 0.01; ***p < 0.001; ****p < 0.0001). Source Data are provided as a Source Data file.

In the asymmetric ICV polymerase (FluCPol) dimer structures^27^, although there is no product RNA, a clear parallel exists with the asymmetric THOVPol dimer structures we present (compare Fig. 6a, b to Fig. 6d, e). The THOVPol structures therefore are the first to confirm that one polymerase functions to synthesize RNA, while the other polymerase functions to receive the RNA product in the asymmetric dimer. Our structural analysis also shows that RNA elongation alone in THOVPol can trigger the re-configuration of the peripheral domains to facilitate the assembly of the asymmetric dimer. In contrast, the assembly of the asymmetric FluCPol dimer has been found to rely heavily on host factor ANP32 proteins, such that FluCPol completely loses activity in ANP32A and ANP32B double knockout cells^27^. The observation that monomeric transcribing polymerase conformations are incompatible with the asymmetric dimer conformation in FluPols has led to the suggestion that the primary function of the asymmetric polymerase dimer is to support genome replication^27,33,45–47^.

To better understand the function of the THOVPol asymmetric dimer, we conducted a structural comparison between THOVPol and FluCPol^27^ dimers, using a superposition based on the THOVPol-RE and FluCPol_EN_ (encapsidating FluCPol) components of the asymmetric dimers. The THOVPol asymmetric dimer structure is formed without the involvement of additional factors, while ANP32A can bind at the FluCPol asymmetric dimer interface^27^. The comparison indicates that the two monomers in the THOVPol dimer are latched closer together (Fig. 6a) than those in the FluCPol dimer (Fig. 6d). There are notable differences between THOVPol and FluCPol in the asymmetric dimer interfaces. Whereas the dimer interface in FluCPol is mainly formed via polar electrostatic and hydrogen bond interactions^27^, the THOVPol dimer is formed via a combination of hydrophobic and polar interactions (Fig. 5b-d). In THOVPol dimer interface 1, the 627 domain of THOVPol-EL is oriented towards the secondary 3′ end binding site of THOVPol-RE and positioned above it (Fig. 6a). In contrast, the PB2 627 domain of FluCPol_RP_ binds away from the secondary 3′ end binding site of FluCPol_EN_ and is in proximity to the PB2 627 and NLS (nuclear localization signal) domains of FluCPol_EN_ (Fig. 6d). Interestingly, within the FluCPol asymmetric dimer, the PB2 NLS of FluCPol_EN_ is instead positioned above the secondary 3′ end binding site of its own monomer. In the THOVPol dimer, away from the PB2 627 domain, (Fig. 6b), the handle-helix is involved in dimer formation (Fig. 6c). Due to the absence of the handle-helix, the FluCPol_EN_ 5′ promoter binding site loop (PA^346^-PA^363^) is not involved in the interactions forming the asymmetric FluCPol dimer (Fig. 6e, f). The above analysis reveals profound differences between THOVPol and FluCPol asymmetric dimers.

To investigate the impact of dimer interface mutations on THOVPol activity, we introduced alanine substitutions into clusters of amino acid residues in interface 2 (F134A^PB2^/W138A^PB2^, Fig. 5c), interface 1 (D547A^PB2^/F549A^PB2^, Fig. 5b) and interfaces 1 and 3 (Q295A^PB2^/D547A^PB2^/I653A^PB2^, Fig. 5b, d). THOV mini-replicon assays show that mutations at the dimer interfaces significantly reduce THOVPol-driven luciferase activity (Fig. 6g). Further RT-qPCR assays reveal that mRNA and cRNA levels are equally affected by the interface mutations, with reductions of at least 100-fold (Fig. 6h).

It was also shown that the FluCPol asymmetric dimer is stabilized by the interaction between the ANP32A N-terminal leucine-rich repeats (ANP32A^LRR^) and PB2, particularly with the PB2 627 domain (Fig. 6d and Supplementary Fig. 12b). The dimerization is further stabilized by additional interactions from ANP32A^LRR^ to the P3 CTD domain of FluCPol_EN_ (Supplementary Fig. 12c, right panel). Due to the shift in the dimer interface and the altered PB2 627 domain orientation in the THOVPol dimer, ANP32A^LRR^ cannot interact with the PB2 627 domain of THOVPol-EL, via the equivalent interactions observed in the FluCPol-ANP32A complex^27^ (Supplementary Fig. 12a–d). In addition, due to a much shorter PA^463-484^ β-hairpin compared with the FluCPol equivalent P3^523-556^, an equivalent interaction to the C-terminal region of ANP32A^LRR^ is not possible in THOVPol (Supplementary Fig. 12c, left panel).

Based on our observation that the FluCPol-ANP32A interaction cannot be maintained in the THOVPol asymmetric dimer and that RNA synthesis alone triggers THOVPol asymmetric dimer formation, we hypothesized that THOVPol may be less dependent on ANP32A for function, in contrast to FluPols. We tested this hypothesis by mini-replicon assays in ANP32A, B and E triple-knockout human 293 T cells (TKO cells). In these TKO cells, the activity of FluAPol is greatly reduced; the expression of hANP32A or hANP32B in the TKO cells is able to enhance FluAPol activity by ∼5000-fold (Fig. 6i, right side columns), while hANP32E has almost no effect on FluAPol activity^24,29^. In contrast, THOVPol maintains higher activity in the TKO cells; expression of hANP32A, hANP32B and hANP32E in the TKO cells increases THOVPol activity by ∼3 - 5-fold (Fig. 6i, left side columns). These results suggest that, unlike FluAPol^21,23,45,46^, FluBPol^24^, and FluCPol^27^, THOV polymerase function is much less reliant on human ANP32A, ANP32B or ANP32E. We further tested the effects of mouse and tick ANP32 proteins on THOVPol activity, finding that their enhancement of THOVPol activity is limited to ~3 - 10-fold.

## Discussion

Replication and transcription of the RNA genome by RNA viruses face continued host cell surveillance. For example, RIG-I surveils 5′ triphosphate containing dsRNA without a cap-1 structure^48^. Influenza viruses employ a cap-snatching mechanism to render their viral mRNA indistinguishable from the host mRNA^16,17^. THOVPol mRNA has no 5′ end extraneous sequence, suggesting that THOV mRNA synthesis most likely starts with short primers. In the THOVPol transcription initiation conformation (Supplementary Fig. 13b), which is similar to a FluPol undergoing cap-snatching transcription initiation^33,35,37,49^ (Supplementary Fig. 13a), the distance (40 Å), between the active sites of the putative cap-snatching domains, is smaller than that between the active sites of the functional cap-snatching domains of FluAPol (56 Å). However, this distance is still substantial and thus incompatible with the suggestion that the putative THOVPol cap-snatching domains may provide short capped primers for THOV transcription. Although cap-snatching activity has been detected for the crudely purified THOV virus core^50^, our structural analysis, in line with a previous structural and biochemical study^39^, has not identified evidence of cap-snatching activity for THOVPol. In this respect, together with the observation that critical catalytic residues are substituted^39^, we are tempted to conclude that cap-snatching is non-functional in THOVPol. Such a proposal may be further supported by the fact that thogotovirus BRBV (Bourbon virus) is not inhibited by inhibitors targeting FluPol cap-snatching activities^51^.

Lack of mRNA 5′ end extraneous sequence also means that THOV mRNA and cRNA only differ at their 5′ ends by the 5′ cap structure. We have demonstrated that THOVPol has the ability to prime RNA synthesis using capped and non-capped dinucleotide primers. Therefore, THOV mRNA and cRNA synthesis could be initiated with short primers. We notice that the THOVPol priming loop has a distinct amino acid sequence and adopts a different conformation during transcription initiation by comparison with that of FluAPol (Supplementary Fig. 13a, b, middle and right panels).

For FluPols, the asymmetric dimer conformation is not compatible with the monomeric, transcription active, cap-snatching active conformation. The bound primer, tethered between the PB2 cap-binding domain and the polymerase active site, presumably prevents the necessary reconfiguration of the peripheral domains (Supplementary Fig. 13a, c) to form the asymmetric dimer associated with influenza virus genome replication^27^. Without structural restraints posed by the primer tethered cap-binding domain as in FluPol (Supplementary Fig. 13c), RNA elongation alone, starting from capped or non-capped short primers, is found to trigger re-configuration of the peripheral domains (Supplementary Fig. 13d) to allow the formation of the THOVPol elongation-reception asymmetric dimer without host factor involvement. Under such circumstances, asymmetric dimerization could be modulated by the availability of free THOVPol. Such a dimerization mechanism implies that during early viral infection, THOVPol should function as a monomer when the free THOVPol concentration is low; however, a switch to a polymerase asymmetric dimer may occur when free THOVPol accumulates. Increased polymerase concentration has been proposed to be an important factor in the switch of polymerase activity from transcription to replication for flu viruses^52,53^.

In summary, despite superficial structural similarities, THOVPol functions very differently from FluPols in RNA synthesis and host-factor utilization. Polymerase activity differences may play a role in growth kinetics differences between thogotoviruses and IAV^54^. It remains unclear how short capped primers are generated in the cell nucleus. Recent evidence suggests that influenza virus preferentially cap-snatches primers from shorter capped RNA fragments generated by the RNA degradation machinery exosome^55^. There is also evidence that exosomes are able to generate capped dinucleotides^56^, although it is unclear whether such capped dinucleotides can be used by THOVPol for mRNA synthesis.

Diverse tick-borne thogotoviruses, shown to replicate in mammalian cells and infect mice, have demonstrated their potential for cross-species transmission^2,57–59^. Numerous human infections, some of which have been fatal, highlight the public health threat posed by thogotoviruses^3–5,60^. Growing evidence suggests that thogotoviruses are adapted well enough to infect mammals^8,57^. The THOVPol structures reported here shed light on the thogotovirus genome transcription and replication. Adaptation to ANP32 proteins has been recognized as a major barrier preventing cross-species transmission of flu viruses. Weak ANP32A/B/E dependence by THOV in RNA synthesis likely contributes thogotoviruses’ adaptability in mammalian cells. This study also highlights the diversity in host-factor utilization and RNA synthesis mechanisms among orthomyxoviruses. These findings should guide the development of broad-spectrum therapeutics targeting the polymerases of thogotoviruses and other orthomyxoviruses.

*Note added in proof*: while this paper was in final revision, structures of influenza virus A and B polymerase oligomers, formed in the presence of ANP32 proteins, were reported^61,62^.

## Methods

### Cells

Human embryonic kidney 293 T cells (ATCC CRL-3216) were purchased from ATCC. ANP32A, B and E triple-knockout human 293 T cells (TKO cells) were generated by coauthor Prof. Xiaojun Wang’s group^24^. The cells were maintained in a humidified atmosphere with 5 % CO_2_ at 37 °C, in Dulbecco’s modified Eagle’s medium (DMEM) supplemented with 10% fetal bovine serum (FBS) and 1% penicillin/streptomycin (Gibco). *Spodoptera frugiperda* (Sf9) cells maintained in SF-900 II SFM (Gibco) were used for generating recombinant baculovirus (rBV) stocks and protein expression. All cell lines used in this study were routinely checked for Mycoplasma and microbial contamination.

### Protein expression and purification

Codon-optimized synthetic genes for THOV (Thogoto virus (isolate SiAr 126)) polymerase subunits with GenBank ID: PA (MT628442.1), PB1 (MT628441.1), PB2 (MT628440.1)], were cloned into the pFast-DUAL vector for co-expression under the control of polH (for PB2 and PB1) and p10 (for PA) promoters. An extension containing a Strep II tag and a 6xHis tag was introduced to the C-terminus of the PB2 subunit. Recombinant bacmid was generated using the Bac-to-Bac expression system. Recombinant baculovirus generation, amplification and protein expression were carried out in Sf9 insect cells. Sf9 cells were infected with recombinant baculovirus, at 48 h post-infection, cells were collected by centrifugation at 3000 rpm for 10 min before lysis by sonication in buffer A (50 mM Tris-HCl pH 8.0, 500 mM NaCl, 5% (v/v) glycerol, 8 mM TCEP) supplemented with protease inhibitor cocktail (Roche, Complete Mini, EDTA-free, 4693132001) and Benzonase Nuclease (Vazyme, RM1022-00). The lysate was cleared by centrifugation (39,190 g for 60 min at 4 °C), before the lysate supernatant was incubated with Strep-Tactin resin (Cytiva, 29401324) for 2 h at 4 °C. The Strep-Tactin resin was washed twice with buffer A before the protein was eluted in buffer B (50 mM Tris-HCl pH 8.0, 300 mM NaCl, 1% (v/v) glycerol, 8 mM TCEP) supplemented with 2.5 mM d-desthiobiotin (Sigma-Aldrich, D1411). Fractions containing the three polymerase subunits were pooled and incubated with Ni Sepharose 6 Fast Flow (Cytiva, 17531806) for 3 h at 4 °C before protein elution with buffer B supplemented with 400 mM imidazole. Fractions containing the target protein were pooled, concentrated and loaded onto a Superose 6 increase 10/300 GL (GE healthcare, 29091596) pre-equilibrated with buffer B. The purified protein was flash-frozen in liquid nitrogen and stored at −80 °C for future use.

### Next-generation sequencing of mRNA 5′ end

HEK293T cells in 6-well plates were co-transfected with expression plasmids encoding THOV polymerase subunits and NP (PA (1 μg), PB1 (1 μg), PB2 (1 μg), and NP (1 μg)) and a plasmid (1 μg) carrying a polI promoter-driven gene cassette containing a green fluorescent protein (GFP) open reading frame (ORF) sequence flanked by THOV 3′ and 5′ vRNA sequences. Total RNA was extracted at 48 h postinfection by an RNAsimple Total RNA kit (TIANGEN, DP419) according to the manufacturer’s instructions. The first-strand synthesis of cDNA was performed using the SMARTer PCR cDNA Synthesis Kit (Takara, 634925). A total of 1 μg RNA was used as the template and incubated at 42 °C for 1 hour in the presence of GFP-specific primers (5′-CGTAGGTCAGGGTGGTCACGA-3′ and template switch oligo (TSO) 5′-ACACTCTTTCCCTACACGACGCTCTTCCGATCTCTAACrGrGrG-3′) and SMARTScribe Reverse Transcriptase for the reverse transcription reaction. cDNA amplification was performed using the Advantage 2 PCR Kit (Takara, 639207) with specific primers designed to include both Illumina adapter sequences and index sequences. The PCR parameters were as follows: initial denaturation step at 95 °C for 1 min, followed by 28 cycles of denaturation at 95 °C for 15 seconds, annealing at 65 °C for 30 s, and extension at 68 °C for 3 min. The final extension step was carried out at 68 °C for 5 min. Final PCR amplification products were subsequently purified, quantified and sequenced on an Illumina HiSeq 6000 for library analysis. A mini-replicon system using A/PR/8/34 (H1N1) IAV polymerase subunits, NP and IAV 3′ and 5′ vRNA sequences was used for comparison.

### Cap-dependent transcription activity assay

THOVPol primer extension assays were based on conditions previously reported for FluAPol^33^ and FluBPol^37^. The purified THOV polymerase at 4.8 μM in a buffer containing 25 mM Tris-HCl (pH 7.5), 300 mM NaCl, 8 mM TCEP, 1 U μl^−1^ RNasin (Thermo Fisher, N8080119), 1% (v/v) glycerol, and 2 mM MnCl_2_ was mixed with 2.4 μM 5′ vRNA, 2.4 μM 3′ vRNA and 0.24 mM m^7^GpppAmG. The reactions were initiated by the addition of either 2.4 mM CTP alone or a combination of 2.4 mM ATP and CTP. The reaction mixture was incubated at 30 °C for 6 h before being quenched with an equal volume of stop solution (95% (v/v) formamide, 20 mM EDTA (pH 8.0), 0.02% (w/v) xylene cyanol)^63^. The mixture was denatured at 95 °C for 5 min and subsequently loaded onto a 22% (w/v) polyacrylamide-7 M urea gel. Following staining with Stains-All (Sigma-Aldrich, E9379), the gel was scanned by Epson Perfection V800 Photo. Details regarding the promoter sequence, primers, and other relevant information can be found in Supplementary Table 1.

### ApG-primed replication activity assay

The purified polymerase at 4.8 μM in a buffer containing 25 mM Tris-HCl (pH 7.5), 300 mM NaCl, 8 mM TCEP, 1 U μl^−1^ RNasin (Thermo Fisher, N8080119), 1% (v/v) glycerol and 2 mM MnCl_2_ was mixed with 2.4 μM 5′ vRNA, 2.4 μM 3′ vRNA and 0.12 mM pppApG. The reaction was initiated by the addition of either 2.4 mM CTP alone or a combination of 2.4 mM ATP and CTP. The reaction mixture was incubated at 30 °C for 6 h. The subsequent sample processing followed the same procedure as described above. Details regarding the promoter sequence, primers, and other relevant information can be found in Supplementary Table 1.

### THOV mini-replicon assay

HEK293T cells in 48-well plates were co-transfected with expression plasmids encoding THOV polymerase subunits and NP (PA (15 ng), PB1 (15 ng), PB2/PB2-HA (15 ng), and NP (75 ng)) and a plasmid (75 ng) carrying a polI promoter-driven gene cassette containing a Firefly luciferase (Fluc) open reading frame (ORF) sequence flanked by THOV 3′ and 5′ vRNA sequences. PB2-HA was constructed to allow quantification of PB2 (polymerase) expression by western blotting. A plasmid (5 ng) expressing Renilla luciferase (Rluc) driven by the SV40 promoter was used as an internal reference reporter plasmid to monitor gene expression and transfection efficiency. To investigate the THOVPol dimer interface mutations, the same plasmids were transfected into cells except that the following PB2-HA mutations were introduced: F134A^PB2^/W138A^PB2^, D547A^PB2^/F549A^PB2^ and Q295A^PB2^/D547A^PB2^/I653A^PB2^. To assess the effect of ANP32 proteins on THOV polymerase activity in TKO cells^24,29^, we supplemented C-terminally HA tagged human ANP32A (hANP32A, NP_006296.1), human ANP32B (hANP32B, NP_006392.1), human hANP32E (hANP32E, NP_112182.1), mouse ANP32A (mANP32A, NP_033802.2), mouse ANP32B (mANP32B, NP_570959.1), mouse mANP32E (mANP32E, NP_075699.3), tick (*Rhipicephalus sanguineus*, brown dog tick, from which THOV has been isolated^3^) ANP32A (tANP32A, XP_037517575.1) and tick ANP32B (tANP32B, XP_037517574.1), respectively. 24 hours after transfection, the activities of firefly luciferase and Renilla luciferase were measured using a Dual-Luciferase Reporter Kit (Promega, E1910). Firefly luciferase activity was normalized to Renilla luciferase activity^29^. PB2 negative control experiments were performed by substituting the PB2 expression plasmid with an empty plasmid. The equivalent A/PR/8/34(H1N1) IAV mini-replicon system was assayed for comparison.

### Western blotting

To assess the effect of dimer interface amino acid mutations on the polymerase expression level, HEK293T cells expressing mutant polymerases were collected 24 h post-transfection. The cells were lysed for 10 minutes at 98 °C with 5x protein loading buffer (Solarbio, P1040). After centrifugation, the samples were separated by SDS-PAGE and subsequently transferred onto a PVDF membrane. After blocking with 5% (w/v) skim milk, the membrane was incubated with a mouse anti-HA monoclonal antibody (1:2000 dilution, Sino Biological, 100028-MM10). This was followed by incubation with a secondary goat anti-mouse antibody conjugated to horseradish peroxidase (1:2000 dilution, Beyotime, A0216). Protein bands were detected by chemical luminescence using Pierce ECL western blotting substrate (Thermo Fisher, 32106). To further evaluate the expression levels of THOV polymerase and ANP32 proteins in 293T TKO cells, cells were collected 24 h post-transfection with the same western blotting procedure as described above. Western blotting of β-actin was used as an internal control.

### RT-qPCR

RNA was extracted from HEK293T cells using the RNAsimple Total RNA kit (TIANGEN, DP419) according to the manufacturer’s instructions. cDNA was synthesized using a HiScript III 1st Strand cDNA Synthesis Kit (Vazyme, R312-01) following the manufacturer’s protocol. Quantitative real-time PCR analysis of mRNA, cRNA and vRNA of luciferase^23^ was conducted using Taq Pro Universal SYBR qPCR Master Mix (Vazyme, Q712-03) on a CFX96 Real-Time PCR Detection System (Bio-Rad). β-actin mRNA served as an internal control. The primer sequences utilized in RT-qPCR are listed in Supplementary Table 2.

### Cryo-EM sample preparation and data collection

To capture THOV polymerase in different conformations, we performed the following reactions by incubating the THOV polymerase with vRNAs and other components based on conditions previously reported for FluAPol^33^ and FluBPol^37^. All reactions were carried out in cryo-EM buffer (25 mM Tris-HCl pH 7.5, 300 mM NaCl, 2 mM MnCl_2_, 8 mM TCEP, 1% (v/v) glycerol).

Transcriptional initiation sample: 3.2 μM THOVPol was incubated at 4 °C for 20 min with 160 μM GMPCPP, 64 μM m7GpppA and 3.2 μM each 5′ and 3′ vRNA. THOVPol was found to adopt transcription pre-initiation and initiation conformations.

Transcriptional elongation sample: 3.2 μM THOVPol was incubated at 30 °C for 6 h with 2.5 μM 3′ vRNA (17-mer), 2.5 μM 5′ vRNA (18-mer), 160 μM capped 2-nucleotide primer (5′-m^7^GpppAmG-3′), 1.6 mM each ATP and CTP.

Replicative elongation sample: 3.2 μM THOVPol was incubated at 30 °C for 6 h with 1.6 μM 3′ vRNA (17-mer), 1.6 μM 5′ vRNA (18-mer), 160 μM 2-nucleotide primer (5′-pppApG-3′), and 1.6 mM each ATP and CTP.

For each reaction, 3 µl of the sample was applied onto each glow-discharged (at 15 mA for 30 s in air, GloCube, Quorum) holey grid (transcriptional initiation sample used Quantifoil R1.2/R1.3, Cu 300 mesh grids; transcriptional elongation and replicative elongation samples used ANTcryo^TM^ R1.2/1.3, Au 300 mesh grids). The grids were blotted for 2.5 s with a force of 4 before plunge-freezing into liquid ethane using a Vitrobot (Thermo Fisher) at 4 °C and 100% humidity. Cryo-grids were loaded onto a 300 keV Titan Krios electron microscope (Thermo Fisher) equipped with a Falcon4 direct electron detector with SelectrisX energy filter (slit width 10 eV) for data collection using EPU. Each movie was collected with the electron event representation (EER) mode and was recorded in counting mode at a ×165,000 magnification, giving a pixel size of 0.73 Å with defocus ranging from −0.6 to −2.4 µm. Gain-normalized movies of 30 frames were collected with a total exposure of ∼50 e^−^/Å^2^.

### Cryo-EM image processing

The following data processing procedures are summarized in Supplementary Fig. 2, Supplementary Fig. 7, and Supplementary Fig. 9. Sample-specific data collection and processing parameters are summarized in Supplementary Tables 3–5.

For the transcriptional initiation sample dataset (Supplementary Fig. 2), within RELION v4.0^64^, movies were motion corrected, dose weighted and CTF estimated. Based on a subset of the micrographs (100 micrographs), particles were reference-free picked by the Laplacian-of-Gaussian filter^65^ and 2D classified. Well-featured particles (40,969 particles) from the subset of the micrographs were selected as templates to train Topaz which picked particles from all the recorded micrographs. Well-featured particles from 2D classification were further selected for 3D classification using a reference based on a cryoSPARC generated initial model. The initial model used was generated using well-featured blob picked particles in cryoSPARC v4.3^66^ using standard settings (Supplementary Fig. 14a). After the first round of 3D classification, the particles of pre-initiation conformation 1 were selected and subjected to two additional rounds of 3D classification to remove bad particles. Particles remaining after excluding pre-initiation conformation 1 particles from the first round of 3D classification were subjected to two rounds of 3D classification. Based on whether the 3′ vRNA enters the polymerase active site cavity, particles were divided into two categories and subjected to further 3D classification. Particles were further divided into 4 classes based on structural features of the PB2 putative cap-binding domain to obtain pre-initiation conformations 2 and 3, initiation conformation and initiation conformation 2. Together with pre-initiation conformation 1, the 5 conformations were subjected to iterative auto-refinement, CTF refinement and Bayesian polishing. Prior to visualization, all density maps were sharpened by applying negative *B*-factors (Supplementary Table 3). The final resolutions of the five structures of pre-initiation conformation 1, pre-initiation conformation 2, pre-initiation conformation 3, initiation conformation and initiation conformation 2 are 2.30 Å, 3.16 Å, 2.78 Å, 3.06 Å, and 2.87 Å, respectively (Supplementary Table 3).

For the transcriptional elongation sample dataset (Supplementary Fig. 7), the same processing steps as the transcriptional initiation sample were used. In RELION v4.0, motion correction, CTF estimation, Topaz-based particle picking and 2D classifications were performed. Approximately 10.1 million particles were selected for the first round of 3D classification using a cryoSPARC generated initial model. The initial model used was generated using well-featured blob picked particles in cryoSPARC v4.3^66^ using standard settings (Supplementary Fig. 14b). After excluding bad particle classes and a class that is identical to pre-initiation class 1, two representative classes were identified based on the presence or absence of a duplex RNA within the polymerase active site cavity. One class, referred to as the receiving conformation, was selected for further 3D classification to remove bad particles. The classes showing duplex RNA within the polymerase active site cavity were subjected to further 3D classification, resulting in two distinct 3D classes: the monomeric transcriptional elongation class and the dimer transcription elongation-reception class. For the monomeric transcriptional elongation class, a further 2 rounds of 3D classification were performed to remove bad particles. The 5 conformations were subjected to 3D refinement, CTF refinement, Bayesian polishing and sharpening. The final resolutions of the three structures for transcription reception conformation, transcription elongation conformation and transcription elongation-reception conformation are 2.52 Å, 2.65 Å, and 2.70 Å, respectively (Supplementary Table 3).

For the replicative elongation sample dataset (Supplementary Fig. 9), movie motion correction was performed using the RELION v4.0^64^ implemented MotionCor2 algorithm. The subsequent processing steps were all performed in cryoSPARC v4.3^66^. Micrographs were subjected to CTF estimation and then manually inspected. Low-quality images were discarded. Particles with diameters ranging from 60 to 180 Å were automatically picked on 500 micrographs. These picked particles were extracted and subjected to a round of 2D classification. Well-featured particles were selected as templates for template picking using a particle diameter of 150 Å in all recorded images. These picked particles were further 2D classified and subjected to multiple rounds of “ab-initio reconstruction” jobs. The best initial model was used for 3D non-uniform refinement. Particles assigned to the 3D refinement were further selected as templates for Topaz^67^ training and picking in all recorded images. Successive “ab-initio reconstruction” jobs were used to eliminate particles displaying poor structural features. Well-featured particles selected through the above procedures were subjected to a final non-uniform refinement and sharpening. For the monomeric replication reception conformation, the data processing workflow is the same as that for the dimer conformation, except that a 100 to 130 Å diameter range was used for blob picking and that a particle diameter of 115 Å was used for template picking. A final dimeric replication elongation-reception map with a resolution of 2.58 Å and a final monomeric replication reception map with a resolution of 2.33 Å were obtained.

### Cryo-EM model building and refinement

The structures of influenza D polymerase^34^ 6KUU and THOVPol PA N-ter^39^ 4CGX were fitted into the transcription pre-initiation conformation 1 map to generate a starting model. The generated structure of pre-initiation conformation 1 and the structures of the THOVPol^39^ PB2 putative cap-binding domain 4CHE and PB2 627 domain 4CHD serve as the initial models for model budling for the other nine structures. Molecular models were visualized and docked into the EM density maps using UCSF Chimera v1.4^68^. Manual adjustment of the models was performed in COOT v0.9.8.1^69^, followed by real-space refinement using PHENIX v.1.20.1^70^. Models of RNAs and ligands were manually placed into the corresponding map densities in COOT. The data processing and refinement statistics are provided in Supplementary Tables 3-5. All structure-related figures were generated using UCSF Chimera^68^ or ChimeraX^71^.

### Reporting summary

Further information on research design is available in the Nature Portfolio Reporting Summary linked to this article.

### Supplementary information

Supplementary Information

Peer Review File

Reporting Summary

### Source data

Source Data

## Data Availability

Cryo-EM density maps and coordinates generated in this study have been deposited in the Electron Microscopy Data Bank (EMDB, https://www.ebi.ac.uk/emdb/) and the Protein Data Bank (PDB, https://www.rcsb.org/) with the following accession numbers: THOVPol transcription pre-initiation conformation 1: EMD-39838 and 8Z85; THOVPol transcription pre-initiation conformation 2: EMD-39848 and 8Z8J; THOVPol transcription pre-initiation conformation 3: EMD-39849 and 8Z8N; THOVPol transcription initiation conformation: EMD-39850 and 8Z8X; THOVPol transcription initiation conformation 2: EMD-39852 and 8Z90; THOVPol transcription elongation conformation: EMD-39855 and 8Z97; THOVPol transcription reception conformation: EMD-39856 and 8Z98; THOVPol transcription elongation-reception conformation: EMD-39862 and 8Z9H; THOVPol replication reception conformation: EMD-39867 and 8Z9Q; THOVPol replication elongation-reception conformation: EMD-39868 and 8Z9R. Source data are provided with this paper.
