# Supplementary material for: Cryo-EM structures of Thogoto virus polymerase reveal unique RNA transcription and replication mechanisms among orthomyxoviruses
Source: Nat Commun. 2024 May 30;15:4620. doi: 10.1038/s41467-024-48848-3 (PMC11139864; doi:10.1038/s41467-024-48848-3)
Supplement: Supplementary file 1 — Supplementary Information [file 41467_2024_48848_MOESM1_ESM.pdf]

Cryo-EM structures of Thogoto virus polymerase reveal unique RNA transcription and replication mechanisms among orthomyxoviruses

Lu Xue[1*], Tiancai Chang[1*], Zimu Li[1,2], Chenchen Wang[3], Heyu Zhao[1], Mei Li[2], Peng Tang[1], Xin Wen[4,5], Mengmeng Yu[6], Jiqin Wu[4], Xichen Bao[1], Xiaojun Wang[6], Peng Gong[4], Jun He[1], Xinwen Chen[2,4#], Xiaoli Xiong[1#]

1. State Key Laboratory of Respiratory Disease, CAS Key Laboratory of Regenerative Biology, Guangdong Provincial Key Laboratory of Stem Cell and Regenerative Medicine, Guangdong Provincial Key Laboratory of Biocomputing, Guangzhou Institutes of Biomedicine and Health, Chinese Academy of Sciences, Guangzhou, Guangdong, China.
2. Guangzhou National Laboratory, Guangzhou, Guangdong, China.
3. College of Medicine, Southern University of Science and Technology, Shenzhen, China
4. Key Laboratory of Special Pathogens and Biosafety, Wuhan Institute of Virology, Center for Biosafety Mega-Science, Chinese Academy of Sciences, Wuhan, Hubei, China
5. University of Chinese Academy of Sciences, Beijing, China
6. State Key Laboratory of Veterinary Biotechnology, Harbin Veterinary Research Institute, Chinese Academy of Agricultural Sciences, Harbin, China.

*These authors contributed equally: Lu Xue, Tiancai Chang.

#e-mail: chen_xinwen@gzlab.ac.cn; xiong_xiaoli@gibh.ac.cn.

This PDF file includes:

Supplementary Figures 1 to 14

Supplementary Tables 1 to 5

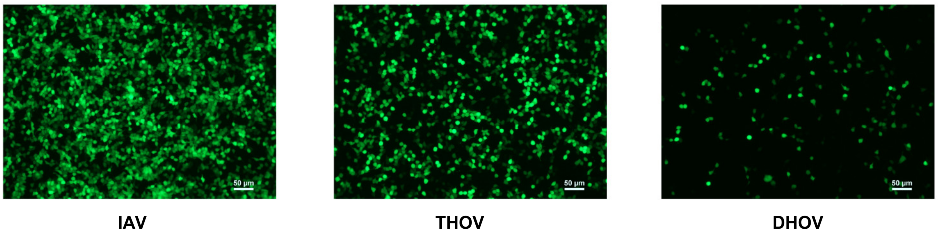

Supplementary Fig. 1 | Mini-replicon systems expressing green fluorescent protein (GFP) driven by influenza A virus (IAV) polymerase (FluAPol) and thogotovirus - THOV, DHOV polymerases, respectively. Representative images from at least 3 independent experiments are shown

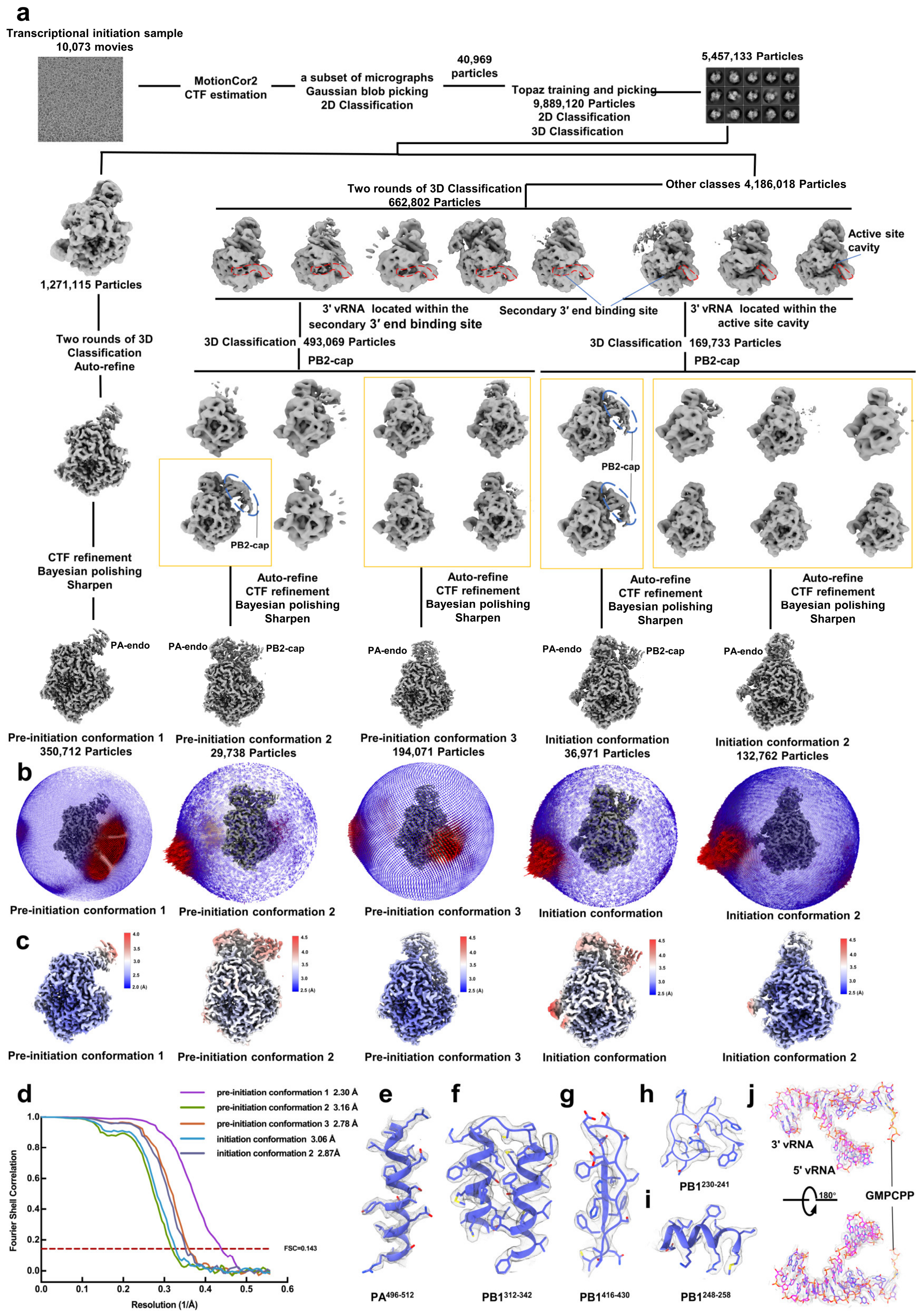

Supplementary Fig. 2 | Cryo-EM data processing and analysis of the THOVPol transcriptional initiation sample. a, Cryo-EM image processing flowchart for the transcriptional initiation sample. Note that "Pre-initiation conformation 3" has almost the same structure as "pre-initiation conformations 2" but without the PB2 putative cap-binding domain resolved. "initiation conformation 2" adopts a structure highly similar

to “initiation conformation” but without the PB2 putative cap-binding domain resolved. **b,** Angular distribution of the particles used for the reconstruction of the polymerase structures. **c**, Local resolution maps for the five distinct transcription initiation conformations. **d**, Fourier shell correlation (FSC) curves of the reconstructed maps for the five transcription initiation conformations. Overall resolutions of the structures were assessed by the gold-standard FSC 0.143 cut-off criteria. **e-j.** Representative densities of structural elements in structures of pre-initiation conformation 1 (**e**), 2 (**f**) and 3 (**g**), initiation conformation (**h and j**) and initiation conformation 2 (**i**).

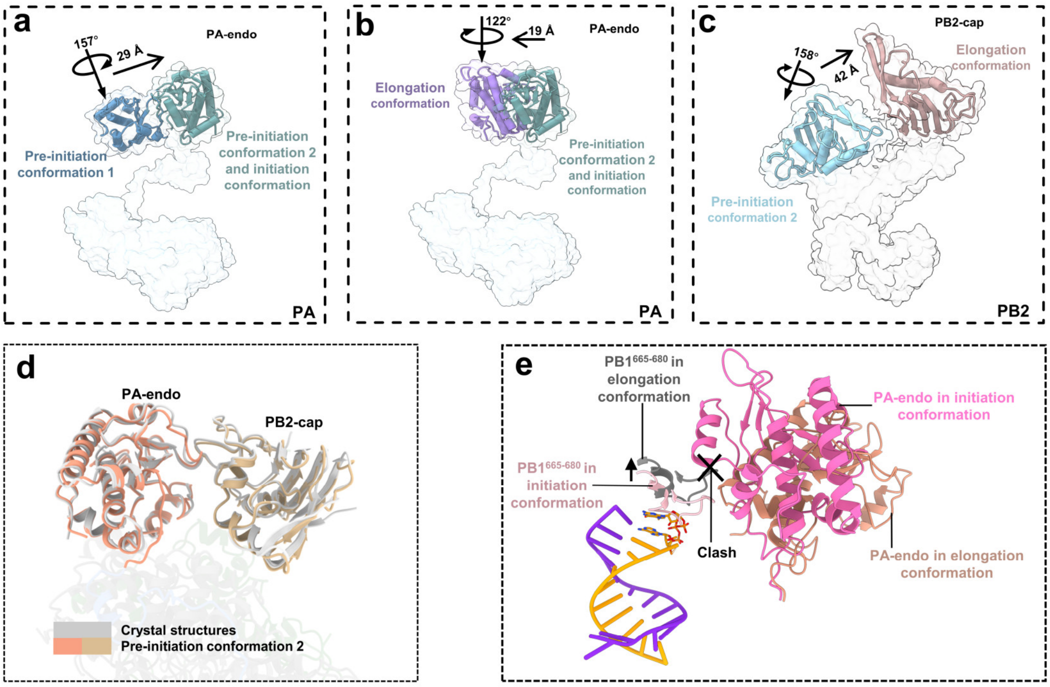

Supplementary Fig. 3 | Movement of peripheral domains of THOVPol among different conformations. a-c, Schematic representations of the relative movement of PA putative endonuclease and PB2 putative cap-binding domains among different conformations relating to THOV RNA transcription. **a**, Movement of PA putative endonuclease domain between Pre-initiation conformation 1 and Pre-initiation conformation 2/Initiation conformation. **b**, Movement of PA putative endonuclease between Pre-initiation conformation 2/initiation conformation and Elongation conformation. **c**, Movement of PB2 putative cap-binding domain between Pre-initiation conformation 2 and Elongation conformation. **d**, Superposition of crystal structures of isolated THOVPol PA putative endonuclease and PB2 putative cap-binding domains onto equivalent structural domains in the cryo-EM structure of THOVPol in Pre-initiation conformation 2, the superposition shows that the domain structures are in agreement with previous crystal structures. **e**, A structural superposition of THOVPol initiation and elongation conformations identifies that RNA elongation induces an upward movement of the $PB1^{665\text{-}680}$ structural motif; this motif clashes with the PA putative endonuclease domain in the initiation conformation; therefore, RNA elongation presumably triggers re-configuration of PA putative endonuclease domain and other peripheral domains in RNA elongation THOVPol-EL conformation.

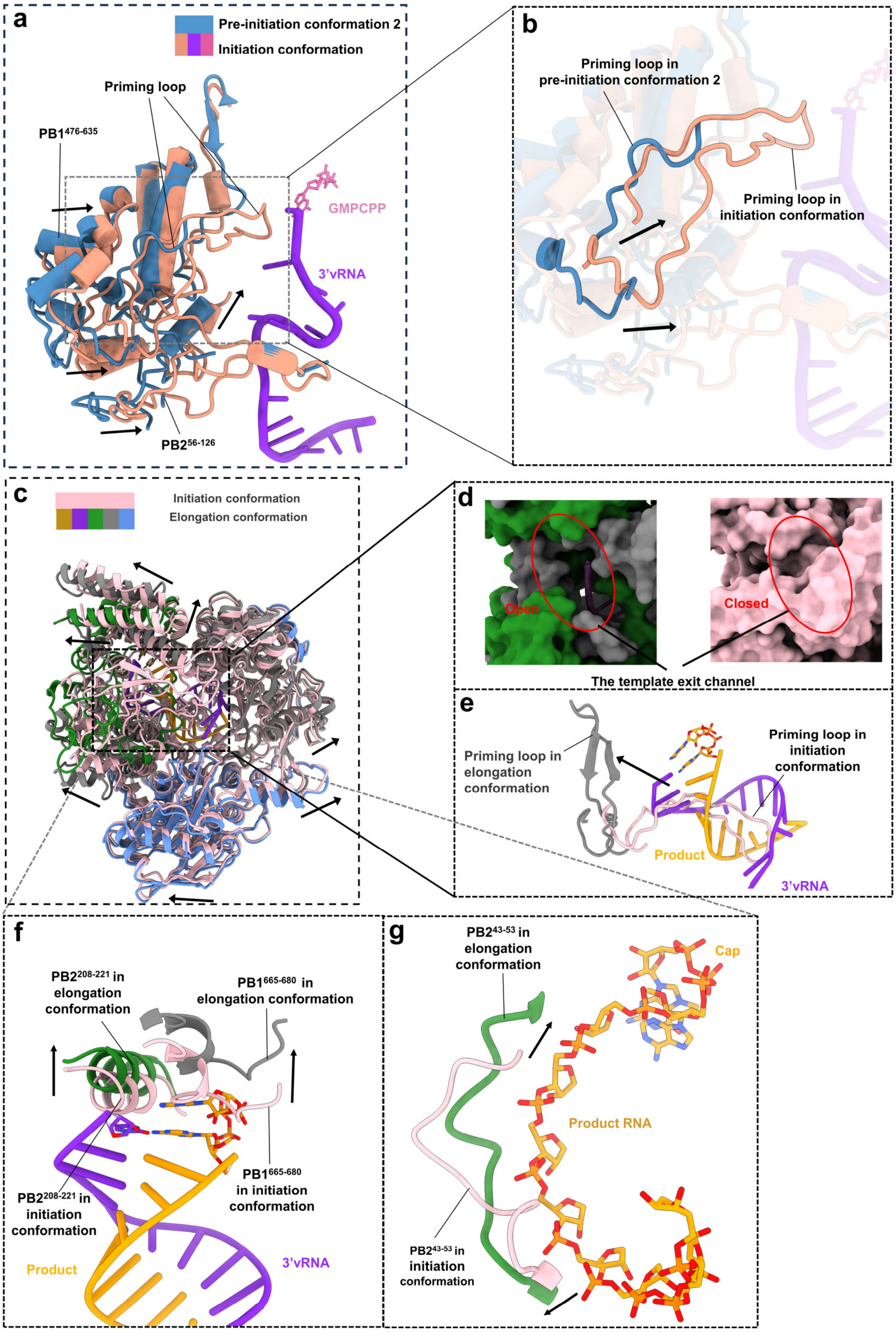

Supplementary Fig. 4 | Movement of structural elements within the THOV polymerase core among different conformations relating to RNA transcription. a and **b**, Comparison of the THOVPol pre-initiation conformation 2 (blue) and the initiation conformation (salmon) structures reveals an obvious deviation of polymerase

core structure (i.e. PB2$^{56-126}$ and PB1$^{476-635}$ containing the priming loop). **c-g**, Superposition of the polymerase core structures in initiation and elongation conformations identifies that the formation of template-product RNA duplex leads to polymerase core opening, causing various structural changes including: unblocking of the template exit channel (**d**); full extrusion of the THOVPol-EL priming loop from active cavity (**e**); an upward movement of the PB2$^{208-221}$ and PB1$^{665-680}$ structural motifs (**f**); stretching of the PB2$^{43-53}$ loop to interact with the phosphate-ribose backbone of the product RNA (**g**).

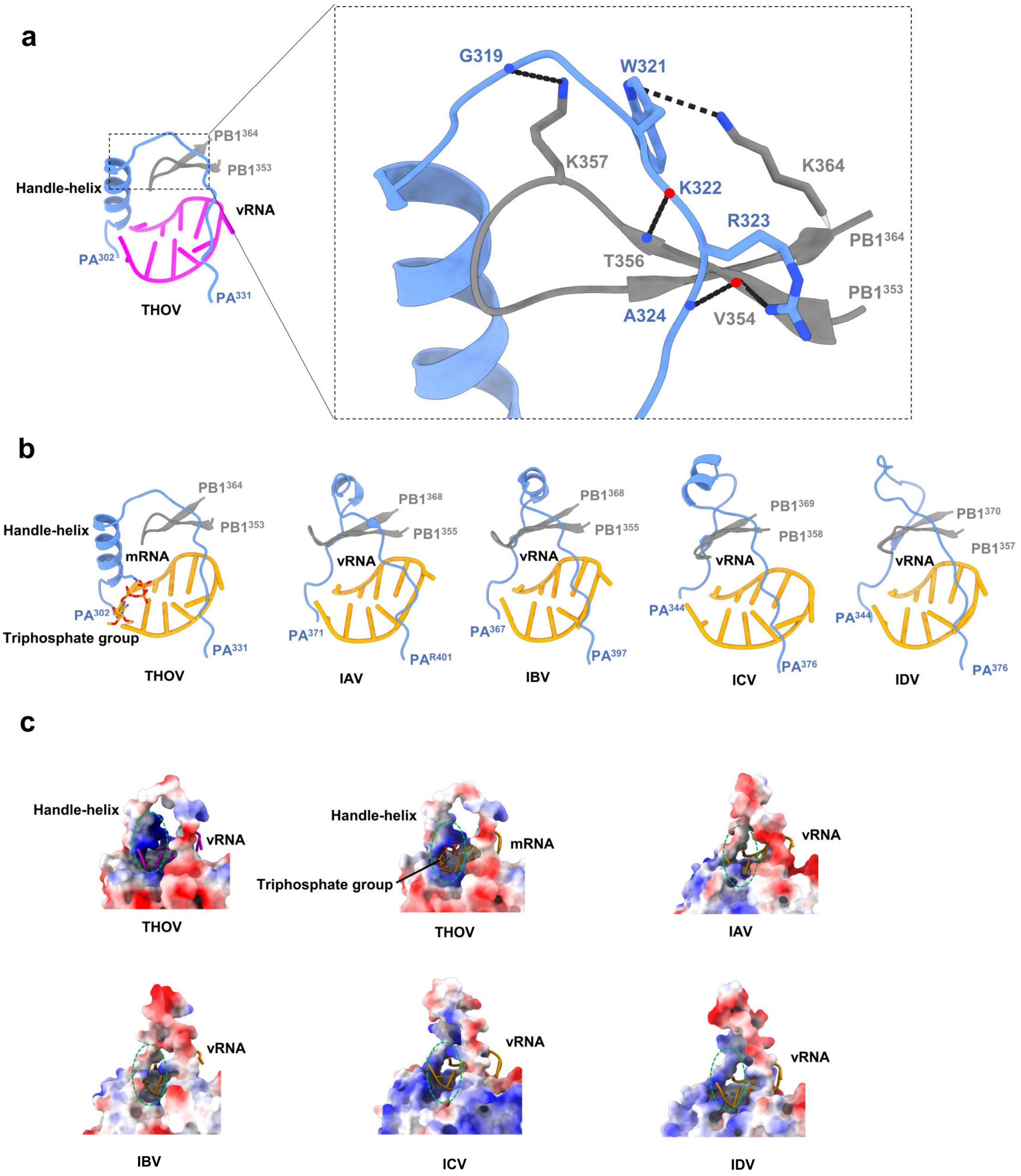

Supplementary Fig. 5 | Comparison of 5′ promoter binding sites in THOVPol and FluPols. a, A cartoon representation of the RNA bound 5′ promoter binding sites of THOVPol. Detailed interactions between the PA handle structure and a β-hairpin from PB1 (PB1$^{353\text{-}364}$) are shown in the dashed box. **b**, A comparison of the RNA bound 5′ promoter binding sites of THOVPol and different FluPols. **c**, Surface representations showing electrostatic potentials around the 5′ promoter binding sites in THOVPol and FluPols.

a

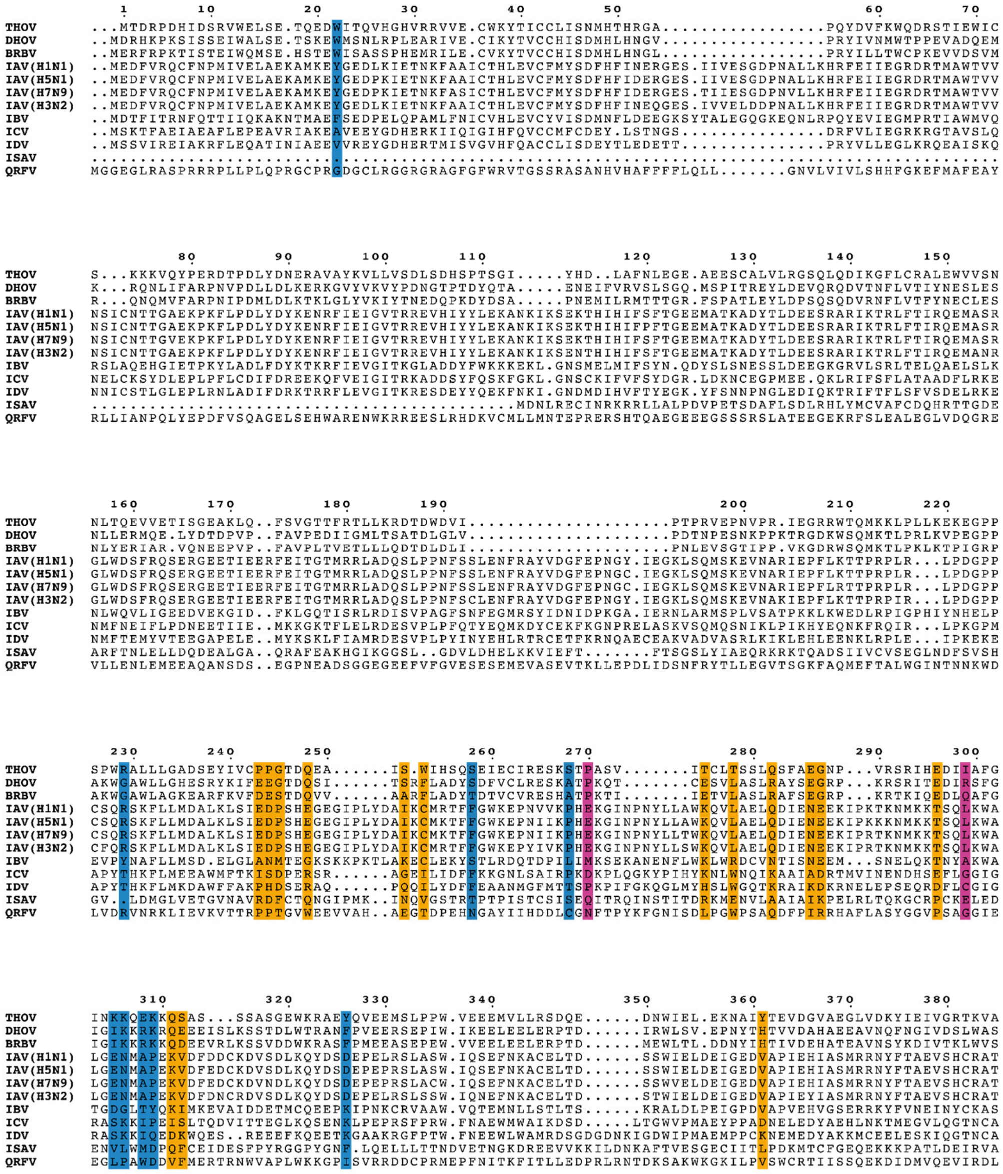
1 10 20 30 40 50 60 70
THOV
DHOV
BRBV
IAV(H1N1)
IAV(H5N1)
IAV(H7N9)
IAV(H3N2)
IBV
ICV
IDV
ISAV
QRFV
80 90 100 110 120 130 140 150
160 170 180 190 200 210 220
230 240 250 260 270 280 290 300
310 320 330 340 350 360 370 380

```
              390       400       410       420                430       440       450       460
THOV        SVIEKWQIAATRTFSQLHTDRSRITACPIITRDP...S.........GNCQFWGMVLLGPHHVKRDTDNAPLLIAEIMGED...TEEKYPKHSV
DHOV        ALVEKMQIAATRAYNELHSDRARVTTVPIITRKEWHGT.........VFSQLWGFVIIGPHHIKQETDRIPIVTVELTDQD...NPEKYPNHSF
BRBV        ALIERMQVAATRAFNELHTDRAKVTTVPIITRKSWKTH.........SLSQLWGFVIVGPHHIKQETDRIPIVTVELVSED...NPEKYPNHSH
IAV(H1N1)   EYIMKGVYINTALLNASCAAMDDFQLIPMISKCRTKEG........RRKTNLYGFIIKGRSHLRNDTDVVNFVSMEFSLTDPRLEPHKWEKYCV
IAV(H5N1)   EYIMKGVYINTALLNASCAAMDDFQLIPMISKCRTKEG........RRKTNLYGFIIKGRSHLRNDTDVVNFVSMEFSLTDPRLEPHKWEKYCV
IAV(H7N9)   EYIMKGVYINTALLNASCAAMDDFQLIPMISKCRTKEG........RRKTNLYGFIIKGRSHLRNDTDVVNFVSMEFSLTDPRLEPHKWEKYCV
IAV(H3N2)   EYIMKGVYINTALLNASCAAMDDFQLIPMISKCRTKEG........RRKTNLYGFIIKGRSHLRNDTDVVNFVSMEFSLTDPRLEPHKWEKYCV
IBV         TVMMKYVLFHTSLLNESNASMGKYKVIPITNRVVNEKG........ESFDMLYGLAVKGQSHLRGDTDVVTVVTFEFSSTDPRVDSGKWPKYTV
ICV         REMGKCILTVGALMTECRLFPGKIKVVPIYARSKERKSMQEGLPVPSEMDCLFGICVKSKSHLNKDDGMYTIITFEFSIREPNLEKH..QKYTV
IDV         REMSKLIHTIGSLHTECRNFPGKVKIVPIYCRGTLRGE.........STDCLFGIAIKGKSHLNKDDGMYTVVTFEFSTEEPNPSKH..EKYTV
ISAV        GE.RFEPSVKPKTQRYGKLSEKWLELEKFIFTASKTEV.DTFLSVGTERLESIGVCVGALHRATTTRIIRPMIQGGKCWGMMFKTKSKMGDTRK
QRFV        GVIGLWEEINANPVKVERTVSALSELMR.LFQEGFREK.NPYISV..KRKCKGWVITGHKQTPALTDTLLELGVGYKPYQMSNVVEEDMRQCET

                          470           480       490       500       510       520             530
THOV        FS...........LKVEEKQFLLS.....LKITSFSRNKLYTFSNIRRVLIQPASIYSQVVLSRAAENNSLNLEVN......PEIQLYLEGAQ.
DHOV        VR...........FLYNKGTESMGHEDLLIRVTSIAKYRLFTFSTIRRVYIQPCSVYSKLILNQSAEAREKDFSMD......KKIEVYLEGRP.
BRBV        FK...........FLYHKGNDSVGHEDVLVRVTSIAKYKLFTFSTIRRVYIQPCSVFSKLILKQSAEKREKDFSLE......NRIEIMINGRP.
IAV(H1N1)   LEIGD.......MLLRSAIGQVSRPMFLYVRTNGTSKIKMKWGMEMRRCLLQSLQQIESMIEAESSVKEKDMTKEFFE....NKSETWPIGESP
IAV(H5N1)   LEIGD.......MLLRTAIGQVSRPMFLYVRTNGTSKIKMKWGMEMRRCLLQSLQQIESMIEAESSVKEKDMTKEFFE....NKSETWPIGESP
IAV(H7N9)   LEIGD.......MLLRTAVGQVSRPMFLYVRTNGTSKIKMKWGMEMRRCLLQSLQQIESMIEAESSVKEKDLTKEFFE....NKSETWPIGESP
IAV(H3N2)   LEIGD.......MLLRSAIGQMSRPMFLYVRTNGTSKIKMKWGMEMRRCLLQSLQQIESMIEAESSVKEKDMTKEFFE....NKSETWPIGESP
IBV         FRIGS.......LF....VSGREKSVYLYCRVNGTNKIQMKWGMEARRCLLQSMQQMEAIVEQESSIQGYDMTKACFKGDRVNSPKTFSIGTQE
ICV         FEAGHTTVR...MKKGESVIGREVPLYLYCRTTALSKIKNDWLSKARRCFITTMDTVETICLRESAKAEENLVEKTLNEK..QMWIGKKNGE..
IDV         FEAGTVPVEAVVLTPKRERVLKEKKLFLYCRTTGMSKLKNDWFSKCRRCLIPTMETVEQIVLKECALKEENRVSEMLENK..RAWIAHENGENL
ISAV        ...EGYCYAIIFGKGEDKSGQNRMTMMGKTVYWHLRVVKSKGDWMAQQLCANKSRIWQHDPELVTEGVTVLMTPFSQKIATISRWRAMRLDSMF
QRFV        DDIEKMKWVDWMSKLAATESEPLGSKVSRDDIFKEAESRHVLDEPAKKMCSKVYDLYRSHKIGATCSKFMGFYSRMGGSYLRAIAGNSRQHSSL

                     540       550       560       570       580       590           600       610       620
THOV        ...RGMTLYQWVRMILCLEFLMAIYNNPQMEGFLANMRRLHMSRHAMMERRQVFLPFGSRPEDK....VNECIINNPIVAYLAKGWNSMPNVYY
DHOV        ...VALSWKSWLIKVFCLEYLMAIHNNPQMEGFLANIRRLHMARHAMMEKAGVYIPHGSAPEEK....CNECVINNPLVAYLARTWNELPNVYM
BRBV        ...VLVTWRSWIIRVLCIEYLMAIHNNPQMEGFLANIRRLHMARHAMMEKSCVFLPFGSAPEDK....CNECIINNPVVAFLARTWNELPNVYM
IAV(H1N1)   KGVEEGSIGKVCRTLLAKSVFNSLYASPQLEGFSAESRKLLLIVQALRDNLEPGTFDLGGLYEA....IEECLINDPWVLLNASWFNSFLTHAL
IAV(H5N1)   KGVEEGSIGKVCRTLLAKSVFNSLYASPQLEGFSAESRRLLLIVQALRDNLEPGTFDLGGLYEA....IEECLINDPWVLLNASWFNSFLTHAL
IAV(H7N9)   KGVEEGSIGKVCRTLLAKSVFNSLYASPQLEGFSAESRKLLLIVQALRDNLEPGTFDLEGLYEA....IEECLINDPWVLLNASWFNSFLTHAL
IAV(H3N2)   KGVEDGSIGKVCRTLLAKSVFNSLYASPQLEGFSAESRKLLLVVQALRDNLEPGTFDLEGLYEA....IEECLINDPWVLLNASWFNSFLTHAL
IBV         GKLVKGSFGKALRVIFTKCLMHYVFGNAQLEGFSAESRRLLLLIQALKDRKGPWVFDLEGMYSG....IEECISNNPWVIQSAYWFNEWLGFEK
ICV         ..LIAQPLREALRVQLVQQFYFCIYNDSQLEGFCNEQKKILMALEGDKKNKSSFGFNPEGLLEK....IEECLINNPMCLFMAQRLNELVIEAS
IDV         TRLVSTKLKDLCRMLIVTQFYYCIYNDNQLEGFCNEQKKFLMFLQADKDSKSAFTFNQKGLYEK....IEECIVSNPLCIFLADRLNKLFLVAK
ISAV        HVSSAWHHSPACEAASA..MLRKFVEIVHAINQKRDWGVVGSMEDMVKEVEEIGEHLQTACDFR.VHNMCKALIQKIAVSTQ............
QRFV        AIMPLYYKTYQEDGTSTRHLTGLVIRGPHHVRESTDTINLLIVEKTCLSRREAQERLSGGALVNGVWWVRKNAIRKADPTYLAFLHNSLFVPTN

THOV        ................................
DHOV        ................................
BRBV        ................................
IAV(H1N1)   R...............................
IAV(H5N1)   R...............................
IAV(H7N9)   R...............................
IAV(H3N2)   R...............................
IBV         EGSKVLESVDEIMDE.................
ICV         KRGAKFFKTD......................
IDV         SNGAKYFE........................
ISAV        ................................
QRFV        FLGELVTTPKHFLRQRQPRILGRNPVWQLWLL
```

b

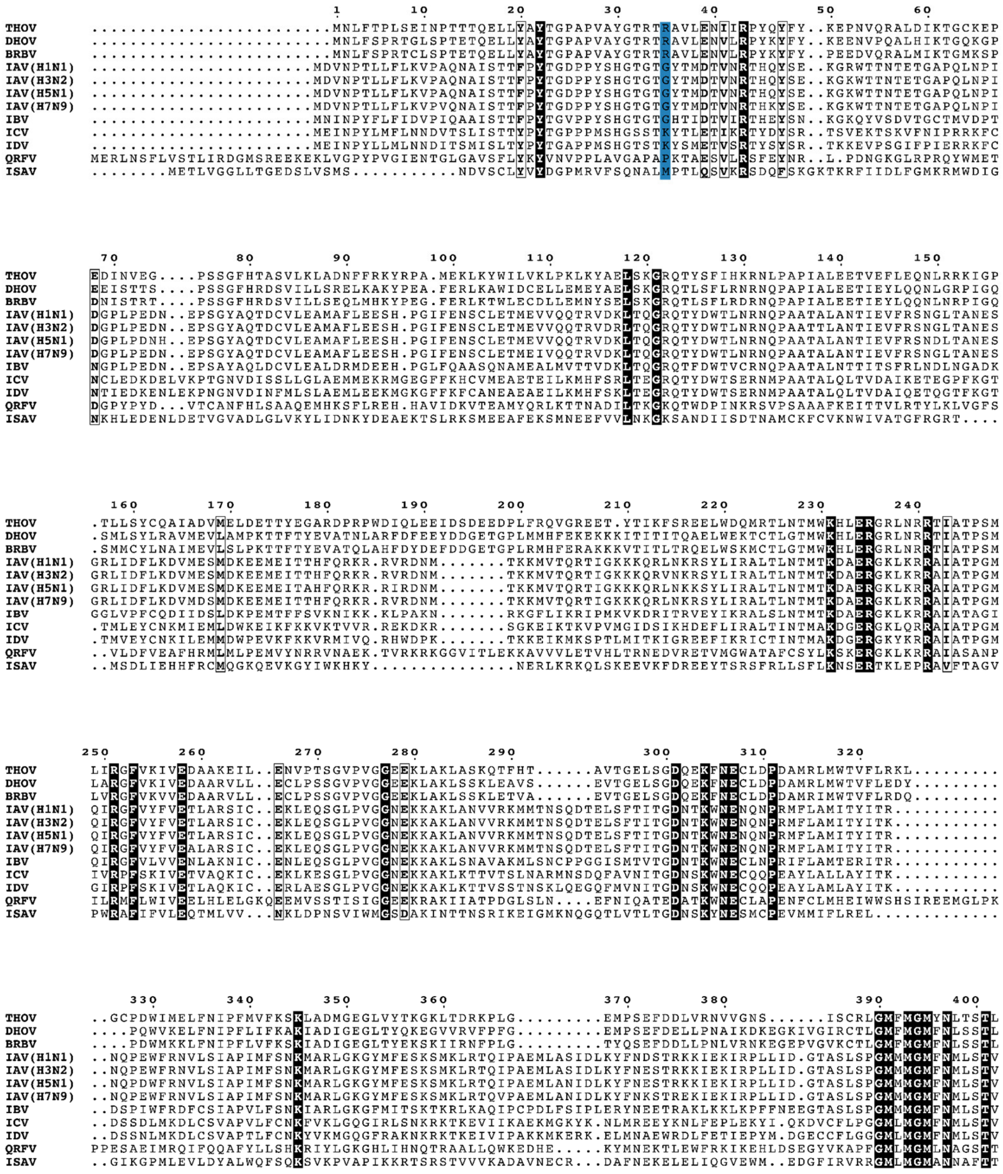
THOV
DHOV
BRBV
IAV(H1N1)
IAV(H3N2)
IAV(H5N1)
IAV(H7N9)
IBV
ICV
IDV
QRFV
ISAV

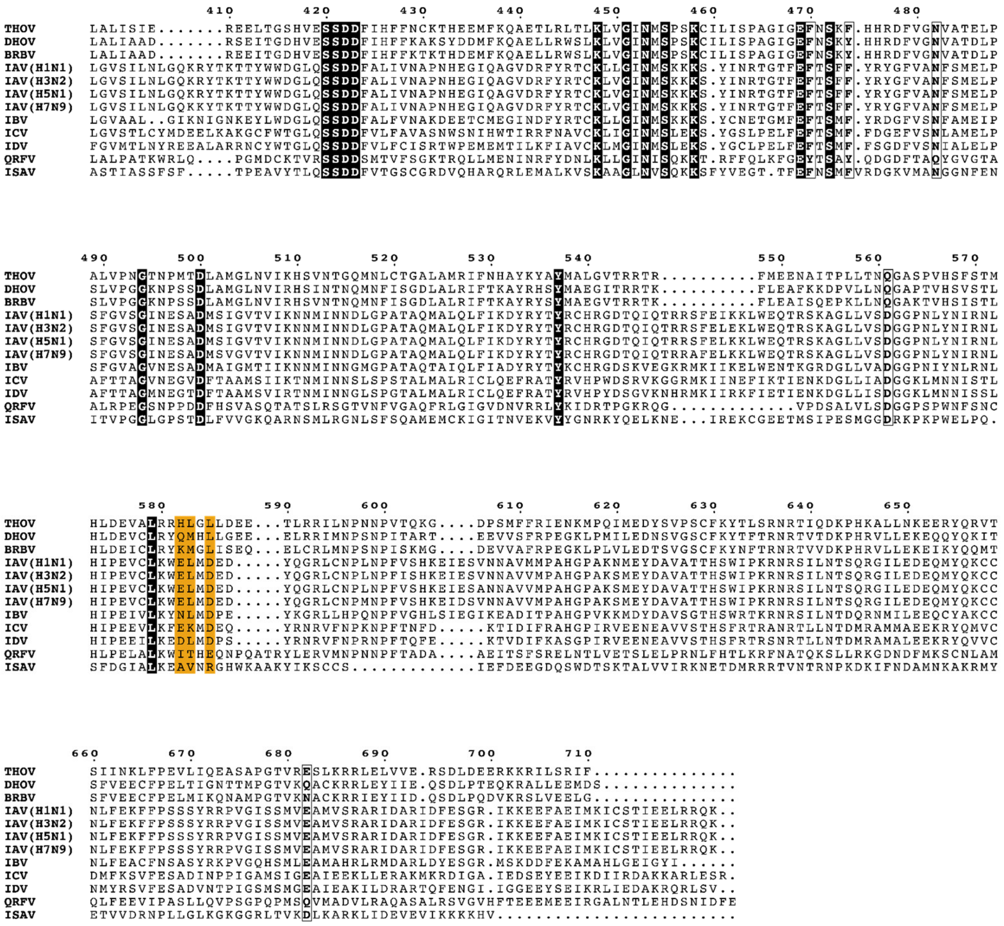

c

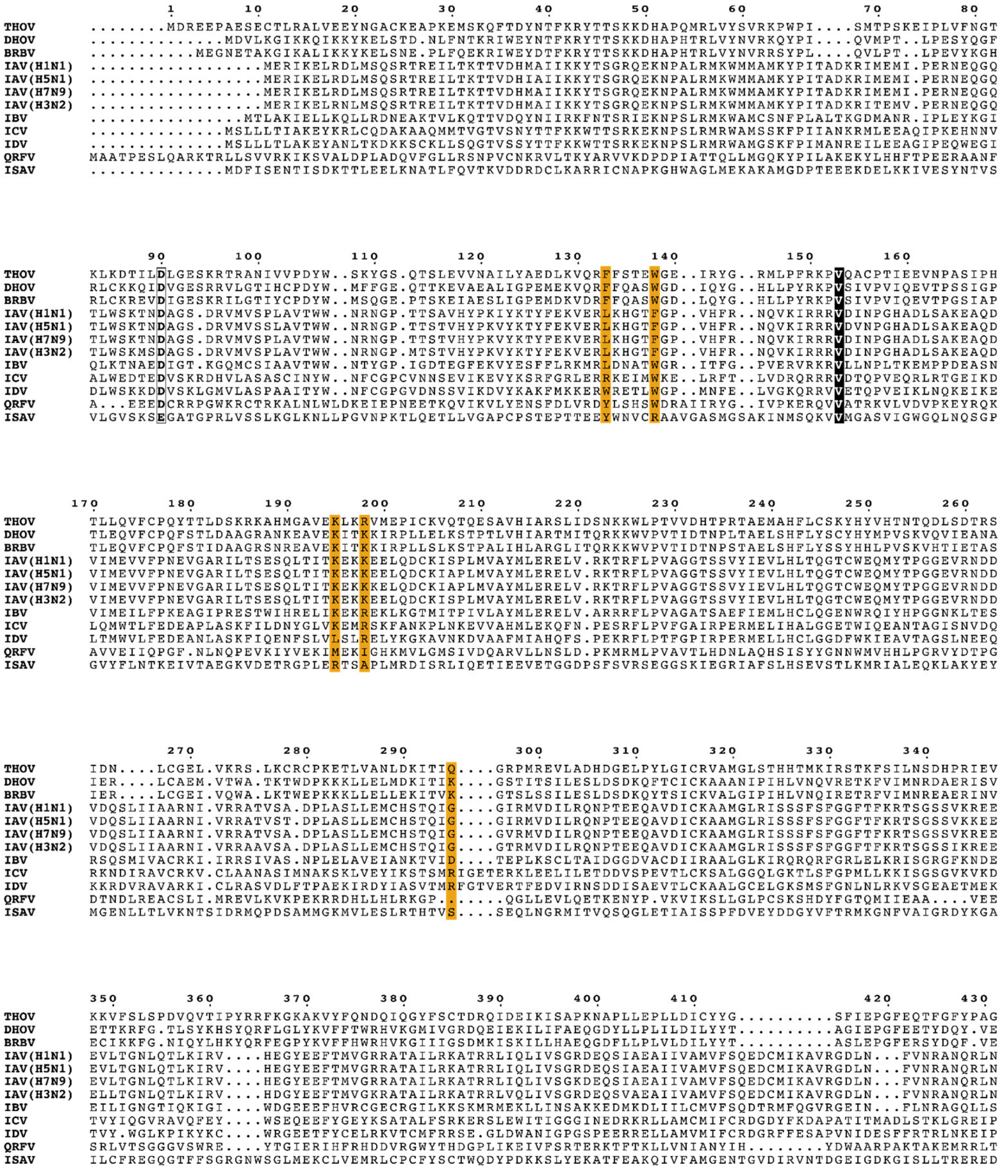

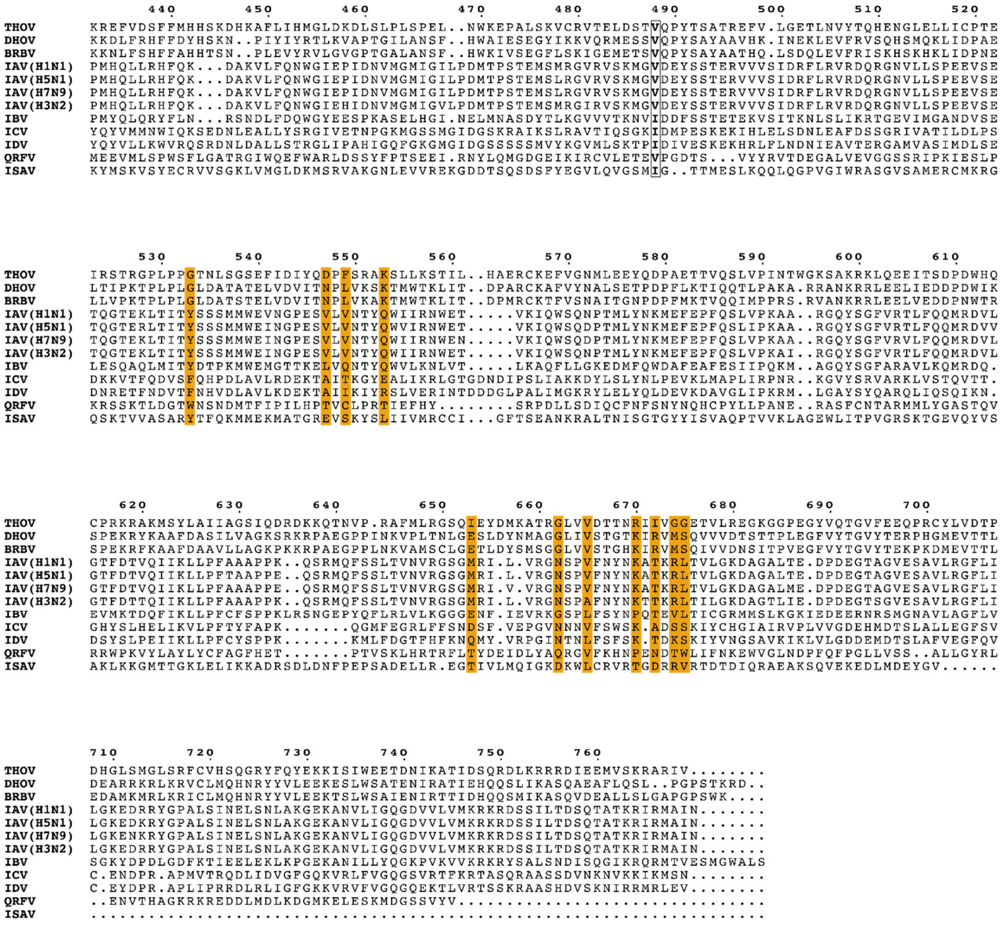

Supplementary Fig. 6 | Sequence alignment of THOV polymerase and polymerases of other orthomyxoviruses. a, Sequence alignment of PA/P3 protein sequences. **b**, Sequence alignment of PB1 protein sequences. **c**, Sequence alignment of PB2 protein sequences. Polymerase sequences used are THOV [Thogoto virus (isolate SiAr 126)], DHOV [Dhori virus isolate Dhori/1313/61], BRBV [Bourbon virus strain Original], IAV(H1N1) [A/Brevig Mission/1/1918 (H1N1)], IAV(H5N1) [A/duck/Fujian/01/2002 (H5N1)], IAV(H7N9) [A/Zhejiang/DTID-ZJU01/2013 (H7N9)], IAV(H3N2) [A/NT/60/1968 (H3N2)], IBV [B/Panama/45/1990], ICV [C/Johannesburg/1/66] and IDV [D/swine/Oklahoma/1334/2011]. Residues in THOVPol involved in the interaction with the 5′ vRNA hook structure and their equivalent residues in the other orthomyxovirus polymerases are highlighted in the blue boxes. Residues involved in forming the asymmetric dimer interface in THOVPol and their equivalent residues in the other orthomyxovirus polymerases are highlighted in the orange boxes. Residues forming the pocket accommodating the cap-1 structure of the product RNA in THOVPol and their equivalent residues in the other orthomyxovirus polymerases are highlighted in the pink boxes. Fully conserved residues among the shown sequences are highlighted in the black boxes.

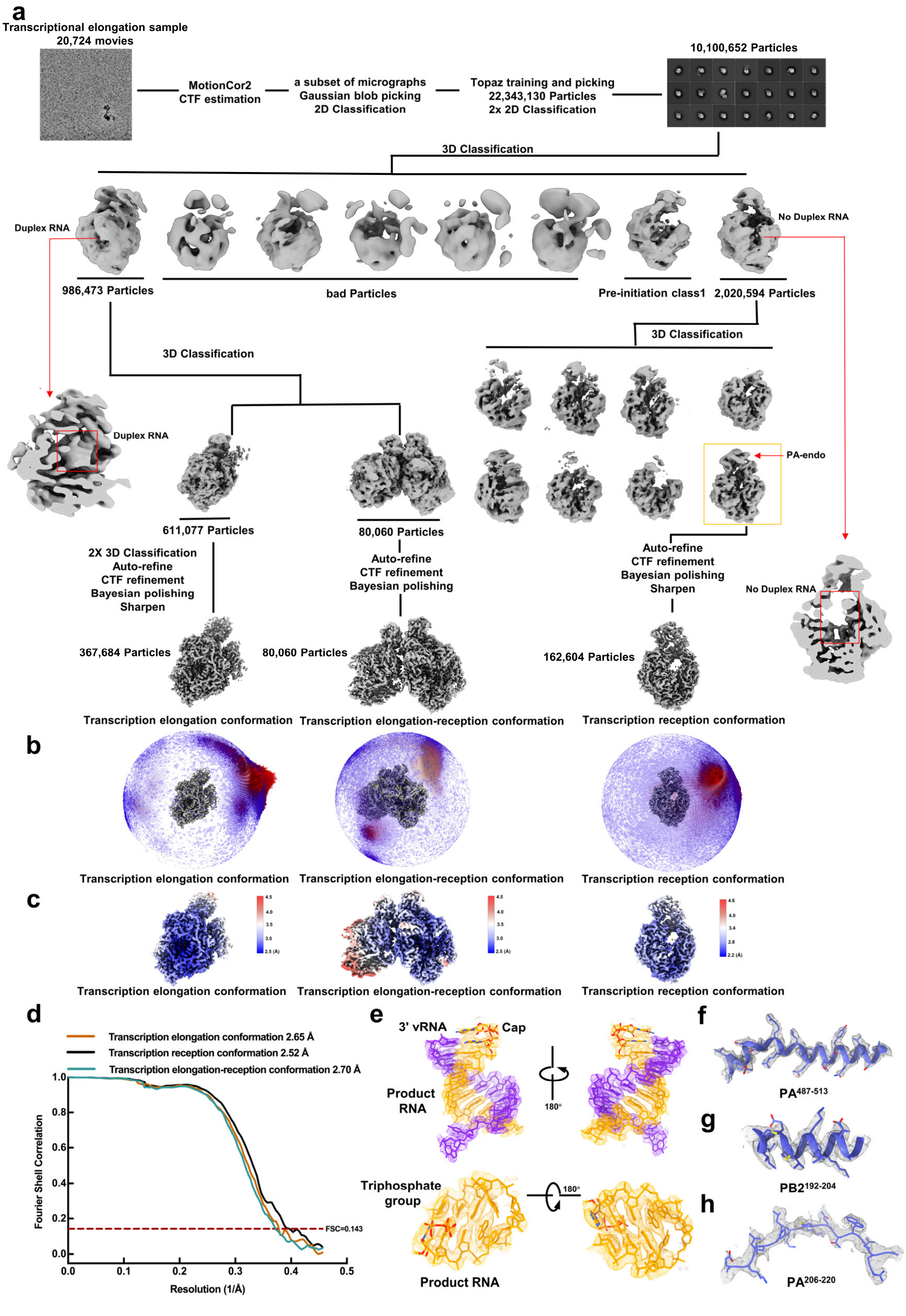

Supplementary Fig. 7 | Cryo-EM data processing and analysis of the THOVPol transcriptional elongation sample. a, Cryo-EM image processing flowchart for the transcriptional elongation sample. **b,** Angular distribution of the particles used for the reconstruction of the polymerase structures**. c**, Local resolution maps for the 3 distinct THOVPol conformations relating to transcriptional elongation. **d**, Fourier shell

correlation (FSC) curves of the reconstructed maps for the three transcriptional elongation conformations. Overall resolutions of the structures were assessed by the gold-standard FSC 0.143 cut-off criteria. **e**, Top, cryo-EM density for the RNA duplex formed between template (violet) and product RNA (orange) in the polymerase active site cavity of the elongation polymerase, THOVPol-EL of asymmetric dimer; bottom, cryo-EM density for the product RNA (orange) bound in the 5′ promoter binding site of the receiving polymerase, THOVPol-RE of asymmetric dimer. **f-h**, Representative densities from maps of THOVPol in transcriptional elongation (THOVPol-EL) (**f**), reception (THOVPol-RE) (**g**) and elongation-reception (THOVPol-EL-THOVPol-RE) asymmetric dimer (**h**) conformations.

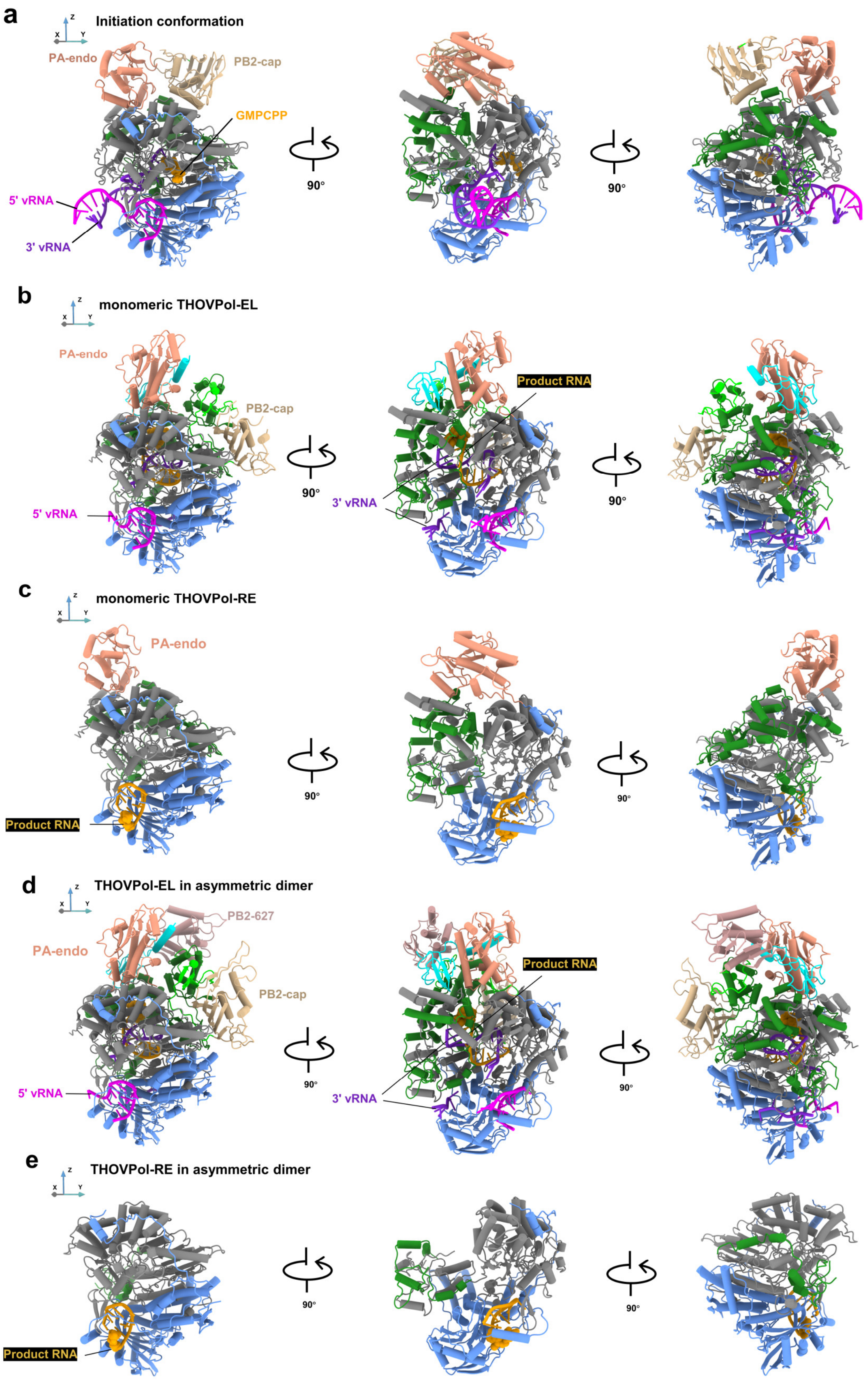

Supplementary Fig. 8 | Comparison of THOVPol structures in different conformations. a, three-views of THOVPol undergoing transcription initiation. **b-e**,

three-views of THOVPol structures obtained from the transcriptional elongation sample.

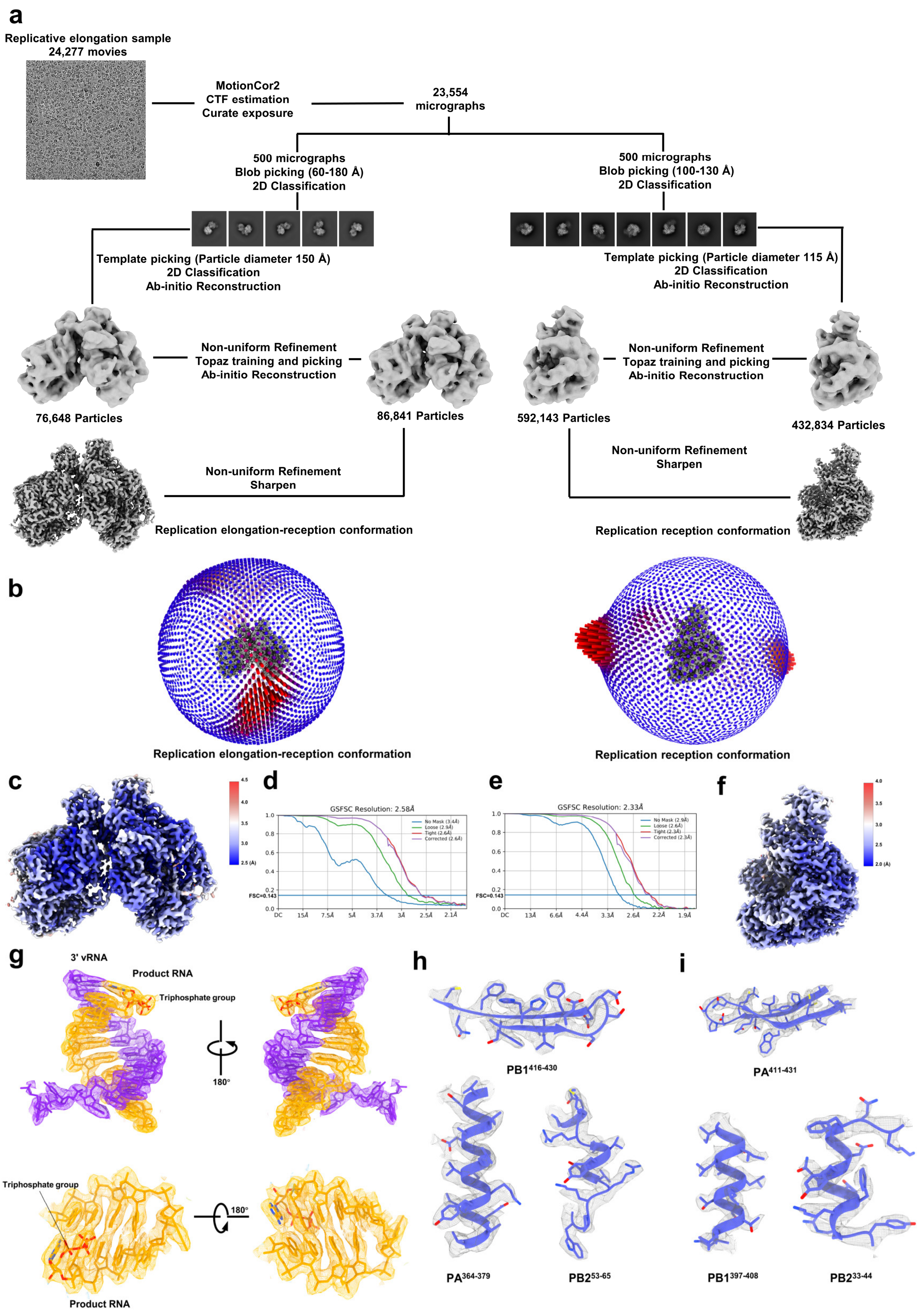

Supplementary Fig. 9 | Cryo-EM data processing and analysis of the THOVPol replicative elongation sample. a, Cryo-EM image processing flowchart for the replication elongation sample. **b,** Angular distribution of the particles used for the reconstruction of the replication elongation-reception asymmetric dimer (THOVPol-EL$_R$-THOVPol-RE$_R$) and the monomeric replication product receiving polymerase

(THOVPol-RE$_{R}$) structures**.** **c**, Local resolution maps for the replication elongation-reception conformation. **d**, Fourier shell correlation curves of the reconstructed map for the replication elongation-reception conformation. Overall resolution of the structure was assessed by the gold-standard FSC 0.143 cut-off criteria **e**, Fourier shell correlation curves of the reconstructed map for the replication reception conformation. **f**, Local resolution map for the replication reception conformation. Overall resolution of the structure was assessed by the gold-standard FSC 0.143 cut-off criteria. **g.** Top, cryo-EM density for the RNA duplex formed between template (violet) and product RNA (orange) in the polymerase active site cavity of the replication elongation polymerase, THOVPol-EL$_{R}$ in the THOVPol-EL$_{R}$-THOVPol-RE$_{R}$ asymmetric dimer; bottom, cryo-EM density for the product RNA (orange) bound in the 5′ promoter binding site of the receiving polymerase, THOVPol-RE in the THOVPol-EL$_{R}$-THOVPol-RE$_{R}$ asymmetric dimer. **h** and **i**, Representative densities from the structures of THOVPol-EL$_{R}$-THOVPol-RE$_{R}$ asymmetric dimer (**h**) and THOVPol-RE$_{R}$ monomer (**i**).

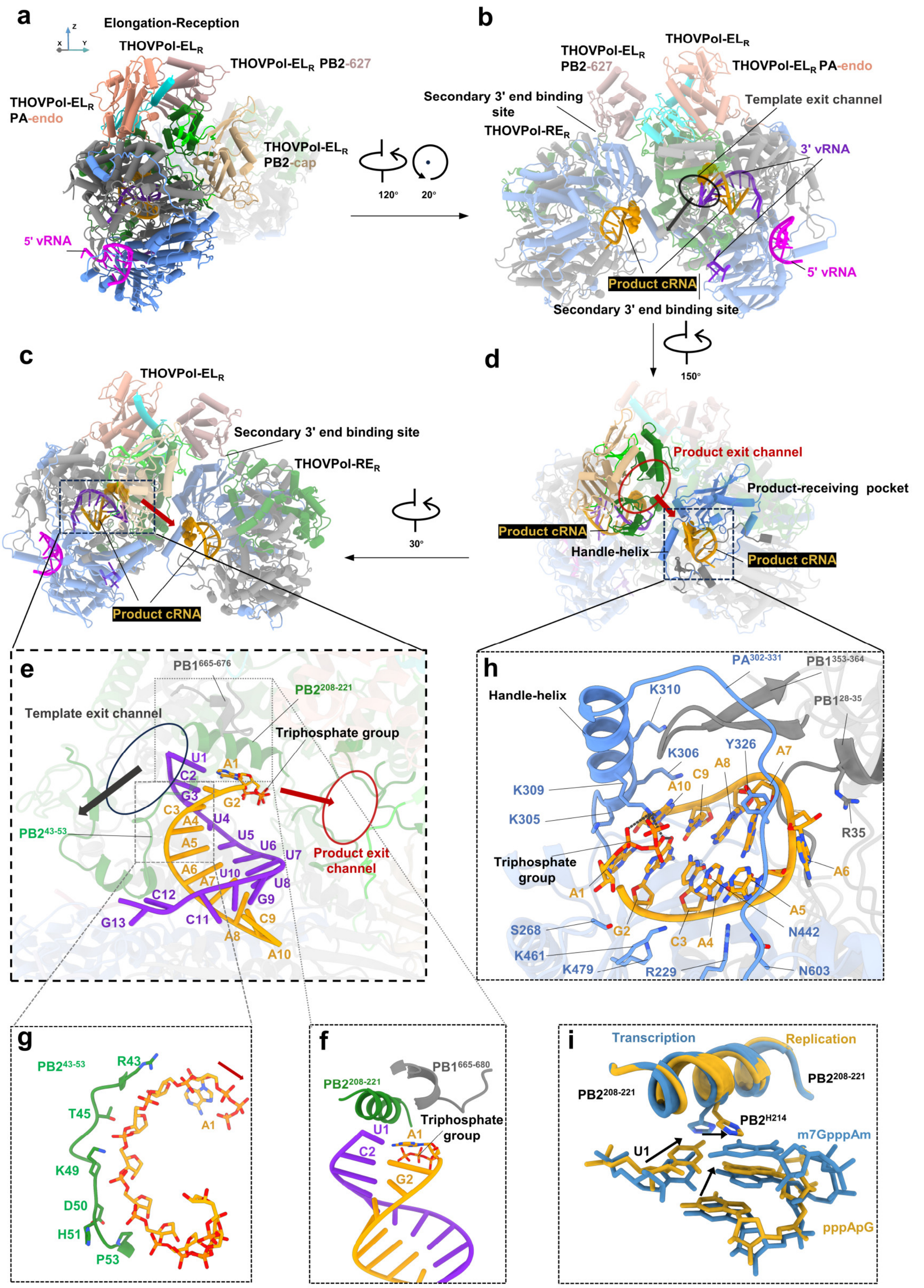

Supplementary Fig. 10 | Structural features of the asymmetric THOV polymerase dimers undergoing replicative RNA synthesis. a-d, Four different views of the THOVPol asymmetric dimer formed during replicative RNA synthesis. The product cRNA is colored orange. The 5′ triphosphate attached nucleotide, pppA, is shown as spheres. The template exit channel in THOVPol-EL$_R$ is circled and the template exit

trajectory is indicated by a black arrow. The product exit channel in THOVPol-EL_R is circled and the product exit trajectory is indicated by a red arrow. **e**, Cartoon representation of the 10-basepair RNA duplex between the cRNA product and the 3′ vRNA template in the polymerase active site cavity. **f**, Two motifs - $PB2^{208\text{-}221}$ and $PB1^{665\text{-}680}$ are found to stack on the template nucleotide U1 and the product cRNA nucleotide A1, respectively. **g**, The stretched $PB2^{43\text{-}53}$ loop allows interactions with the phosphate-ribose backbone of the cRNA product. The residues interacting with the phosphate-ribose backbone of the cRNA are depicted as sticks. **h**, Close-up view of detailed interactions between product cRNA and product-receiving pocket (5′ promoter binding site). The 5′-triphosphate group attached to nucleotide A1 at the 5′ end of product cRNA are depicted as sticks. Sidechains of the RNA interacting residues from the product-receiving pocket are shown as sticks, they form similar interactions with bound 5′ vRNA (see **Fig. 2e**); hydrogen bonds and salt-bridges formed by them are omitted for clarity. Hydrogen bonds and salt-bridges between the 5′-triphosphate group of the product cRNA and the 5′ promoter binding site handle-helix are shown by dashed lines. **i**, Superposition of template-product RNA duplexes in the replicative elongation (gold) and the transcriptional elongation (blue) structures. The comparison reveals that, due to the lack of the cap nucleotide, the cRNA product interacts differently with the $PB2^{208\text{-}221}$ helix within the polymerase active site cavity by comparison with the capped mRNA product.

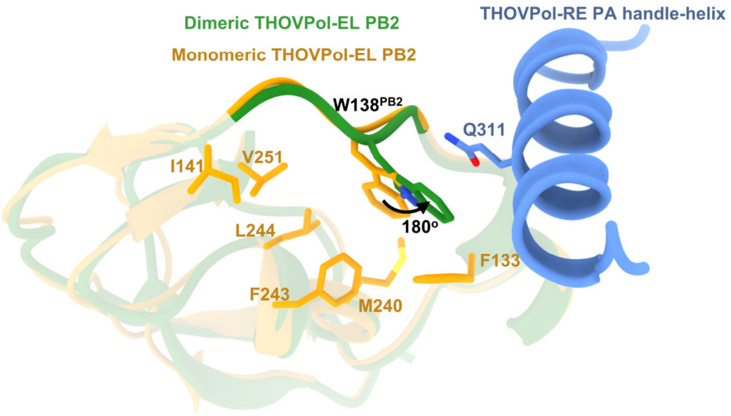

Supplementary Fig. 11 | Conformational change of the THOVPol-EL W138^{PB2} sidechain upon asymmetric dimer formation. a, The sidechain of W138^{PB2} is rotated by ~180° upon dimer formation to engage in an amide-π interaction with Q311^{PA} of the THOVPol-RE handle-helix.

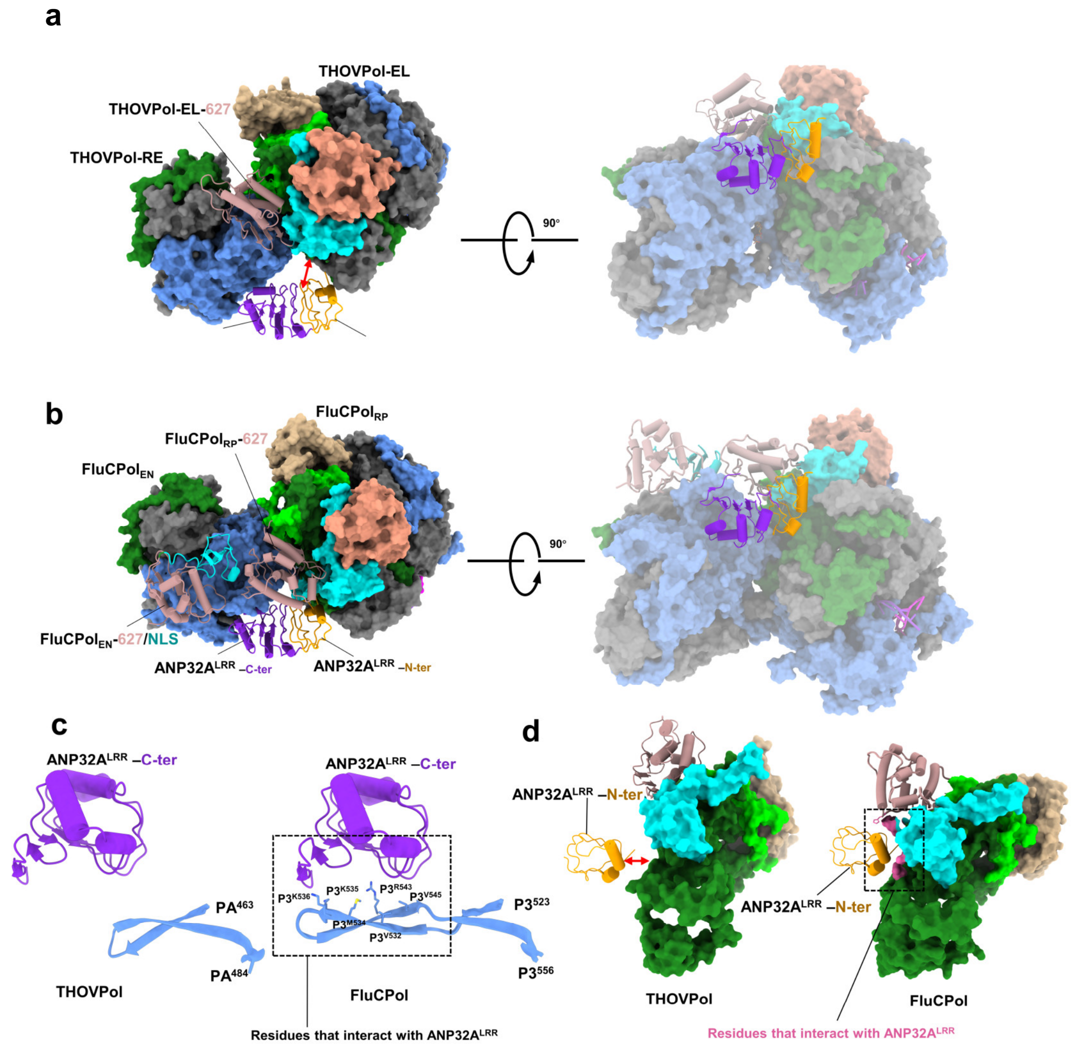

Supplementary Fig. 12 | THOVPol is unable to bind ANP32A with the same interaction mode as in FluCPol. a and b, Up-and-down comparison of the asymmetric dimer structures of THOVPol (**a**) and FluCPol (**b**) (PDB 6XZQ) based on the superposition of THOVPol-RE and FluCPol$_{EN}$. In **a**, an ANP32A molecule is modelled in the equivalent position as found in the FluCPol structure. **c**, The β-strand formed by PA$^{463\text{-}484}$ in THOVPol-RE is shorter compared to the β-strand formed by PA$^{523\text{-}556}$ in FluCPol$_{EN}$. The missing portion is involved in the interaction with the C-terminal domain of ANP32A^{LRR}, suggesting that THOVPol PA$^{463\text{-}484}$ is unable to bind ANP32A^{LRR} with the same interactions as identified in the FluCPol structure. **d**, Schematic representations of the relative position of ANP32A^{LRR} in relation to THOVPol-RE (left) and FluCPol$_{RP}$ (right). The space between the N-terminal domain of modelled ANP32A^{LRR} and THOVPol-EL indicates a lack of interaction between THOVPol and ANP32A in the interaction mode as identified in the FluCPol structure. The surfaces containing residues that interact with C-terminal domain of ANP32A^{LRR} in FluCPol$_{RP}$ are highlighted pink.

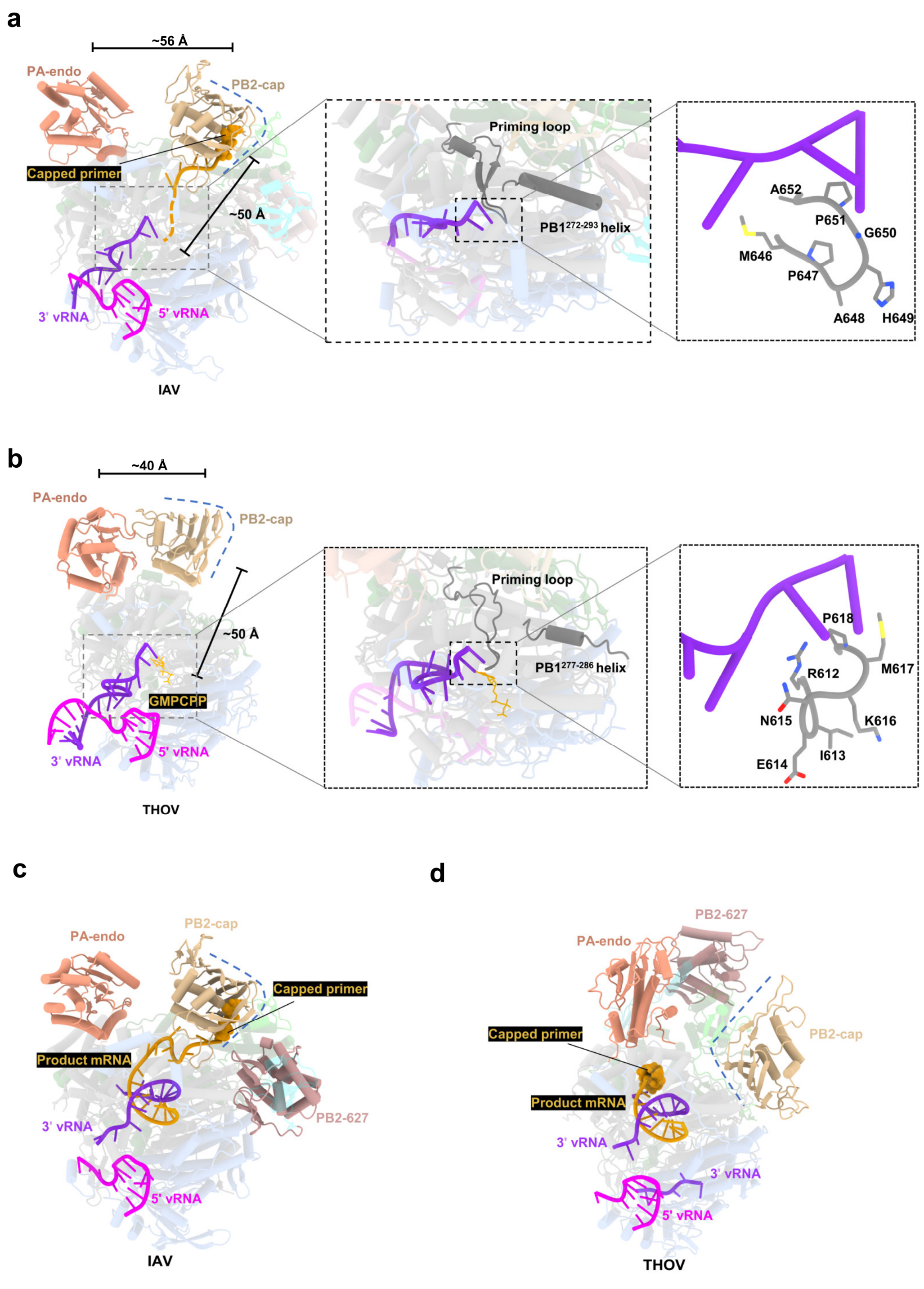

Supplementary Fig. 13 | Comparison of polymerase structures relating to RNA transcription between IAV and THOV. a-b, Structural comparison of the FluAPol transcription initiation conformation (PDB:6RR7) (**a**) and the THOVPol transcription initiation conformation (**b**). Distances between the active sites of cap-snatching domains and distances between the polymerase active site and cap-binding domain active site are indicated by sold-lines. The dashed lines indicate the cap-binding domain molecular surfaces on which active site or putative active site are located. **c-d**, Structural comparison of the FluAPol transcriptional elongation conformation

(PDB:7QTL) (**c**) and the THOVPol transcriptional elongation conformation (**d**). **c**, the polymerase has structural restraints posed by the primer tethered PB2 cap-binding domain such that peripheral domains are unable to adopt a configuration to allow asymmetric dimer formation. **d**, In THOVPol, due to likely non-functional cap-snatching domains, elongation of short primer alone is found to induce a peripheral domain configuration to allow asymmetric dimer formation.

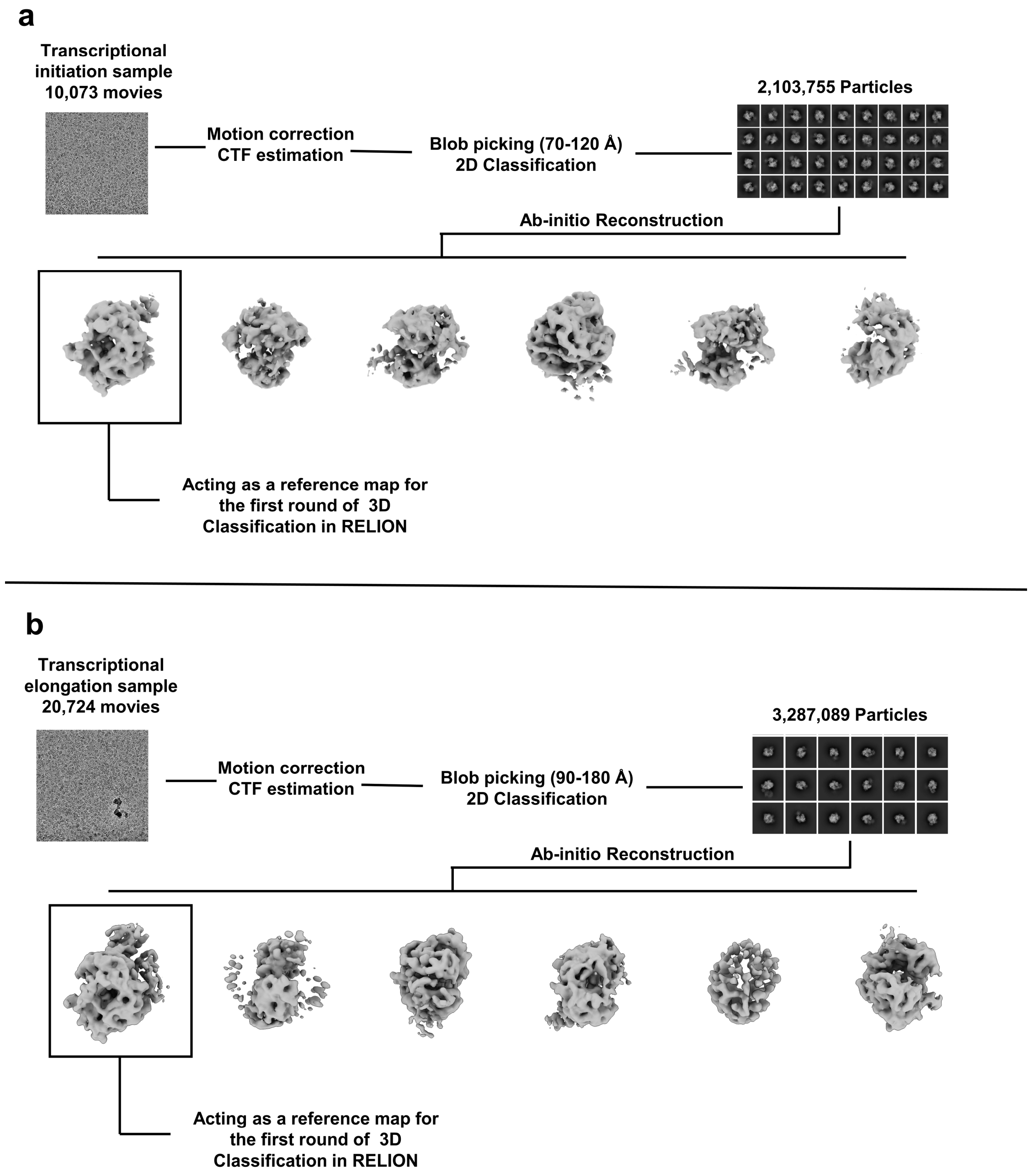

Supplementary Fig. 14 | Flowcharts for initial model generation by cryoSPARC. Procedures of initial model generation for transcriptional initiation (**a**) and elongation (**b**) datasets.

Supplementary Table 1. Oligonucleotides used in this study.

Primer	Sequence (5'-3')	Source
3' vRNA	GACUGCCUGUUUUUGCU	GenScript
5' vRNA	AGAGAAAUCAAGGCAGUU	GenScript
Capped primer	(m7Gppp)A	NEB
Capped primer	(m7Gppp)AmG	TriLink Biotechnologies
Uncapped primer	pppApG	Jena Bioscience
Marker-7	pAGCAAAA	GenScript
Marker-8	pAGCAAAAA	GenScript
Marker-10	pGACUGCCUGU	GenScript
Marker-13	AGAGAAAUCAAGG	GenScript
Marker-17	GACAGCCGGGAUAAGAG	GenScript
Marker-20	CGUAACUGCUUGUUUUUGCU	GenScript
Marker-23	AGAGAAAUCAAAGCAGUUUUUCU	GenScript
ATP		Thermo Fisher
CTP		Thermo Fisher
GMPCPP		Jena Bioscience

Supplementary Table 2. List of primers used for RT-qPCR.

Primer	Sequence (5'-3')
vRNA	CATTTCGCAGCCTACCGTGGTGTT
cRNA	AGAGAAATCAAGGCAGT
mRNA	Oligo-dT_{20}
luc-PCR-F	GATTACCAGGGATTTCAGTCG
luc-PCR-R	GACACCTTTAGGCAGACCAG
β-actin-F	CTCCTCCTGAGCGCAAGTACTC
β-actin-R	TCCTGCTTGCTGATCCACATC

Supplementary Table 3. Cryo-EM data collection, refinement and validation statistics.

	THOVPol pre-initiation conformation 1	THOVPol pre-initiation conformation 2	THOVPol pre-initiation conformation 3	THOVPol initiation conformation	THOVPol initiation conformation 2
**Data collection and processing**					
Magnification			165000		
Voltage (kV)			300		
Electron exposure (e^{-}/Å^2)			50		
Defocus range (μm)			0.6-2.4		
Pixel size (Å)			0.73		
Movies (no.)			10073		
Initial particle images (no.)			9889120		
Symmetry imposed			*C1*		
Final particle images (no.)	350712	29738	194071	36971	132762
Map resolution (Å)	2.30	3.16	2.78	3.06	2.87
FSC threshold	0.143	0.143	0.143	0.143	0.143
Map resolution range (Å)	2.20-4.01	3.02-5.63	2.68-4.44	2.88-5.05	2.70-4.24
**Refinement**					
Initial model used	PDB: 6KUU, 4CGX	PDB: 6KUU, 4CHE, 4CGX	PDB: 6KUU, 4CGX	PDB: 6KUU, 4CHE, 4CGX	PDB: 6KUU, 4CGX
Model resolution (Å)	2.34	3.21	2.92	3.12	2.98
FSC threshold	0.5	0.5	0.5	0.5	0.5
Map sharpening *B* factor (Å^2)	-44	-61	-70	-59	-70
Model composition					
Non-hydrogen atoms	10467	13766	12485	14768	13481
Protein residues	1256	1665	1510	1730	1573
Ligands	0	0	0	1	1
Nucleotides (RNA)	18	15	15	35	35
B factors (Å^2)					
Protein	96.99	96.78	72.38	100.59	73.68
Nucleotide (RNA)	75.18	86.05	64.67	116.08	99.04
Ligand	-	-	-	112.33	89.85
R.m.s. deviations					
Bond lengths (Å)	0.002	0.002	0.003	0.003	0.003
Bond angles (°)	0.483	0.548	0.557	0.610	0.666
**Validation**					
MolProbity score	1.49	1.84	1.83	1.94	1.81
Clash score	7.23	9.60	10.03	12.18	10.72
Poor rotamers (%)	0	0	0		
Ramachandran plot					
Favored (%)	97.58	95.26	95.58	95.06	96.18
Allowed (%)	2.42	4.74	4.42	4.94	3.82
Disallowed (%)	0	0	0	0	0

Supplementary Table 4. Cryo-EM data collection, refinement and validation statistics.

	THOVPol transcription elongation	THOVPol transcription elongation-reception	THOVPol transcription reception
**Data collection and processing**			
Magnification		165000	
Voltage (kV)		300	
Electron exposure (e^{-}/Å^2)		50	
Defocus range (μm)		0.6-2.4	
Pixel size (Å)		0.73	
Movies (no.)		20724	
Initial particle images (no.)		22343130	
Symmetry imposed		*C1*	
Final particle images (no.)	367684	80060	162604
Map resolution (Å)	2.65	2.70	2.52
FSC threshold	0.143	0.143	0.143
Map resolution range (Å)	2.46-5.71	2.54-4.86	2.39-4.41
**Refinement**			
Initial model used	PDB: 6KUU, 4CHE, 4CGX	PDB: 6KUU, 4CHE 4CHD, 4CGX	PDB: 6KUU, 4CGX
Model resolution (Å)	2.68	2.87	2.64
FSC threshold	0.5	0.5	0.5
Map sharpening *B* factor (Å^2)	-32	-35	-32
Model composition			
Non-hydrogen atoms	15732	26475	12363
Protein residues	1843	3153	1505
Ligands	1	2	1
Nucleotide (RNA)	39	48	9
B factors (Å^2)			
Protein	87.44	107.30	73.88
Nucleotide (RNA)	82.46	85.55	91.51
Ligand	135.63	101.92	94.16
R.m.s. deviations			
Bond lengths (Å)	0.002	0.002	0.003
Bond angles (°)	0.542	0.570	0.530
**Validation**			
MolProbity score	1.92	1.85	1.69
Clash score	10.57	9.95	8.69
Poor rotamers (%)	0	0	0
Ramachandran plot			
Favored (%)	94.52	95.24	96.57
Allowed (%)	5.48	4.76	3.43
Disallowed (%)	0	0	0

Supplementary Table 5. Cryo-EM data collection, refinement and validation statistics.

	THOVPol replication elongation-reception	THOVPol replication reception
**Data collection and processing**		
Magnification	165000	
Voltage (kV)	300	
Electron exposure ($e^{-}/Å^2$)	50	
Defocus range (μm)	0.6-2.4	
Pixel size (Å)	0.73	
Movies (no.)	24277	
Initial particle images (no.)	11337414	20119573
Symmetry imposed	*C1*	*C1*
Final particle images (no.)	86841	592143
Map resolution (Å)	2.58	2.33
FSC threshold	0.143	0.143
Map resolution range (Å)	2.19-40.99	2.03-36.36
**Refinement**		
Initial model used	PDB 6KUU, 4CHE, 4CHD, 4CGX	PDB 6KUU, 4CGX
Model resolution (Å)	2.89	2.60
FSC threshold	0.5	0.5
Map sharpening *B* factor ($Å^2$)	-72	-91
Model composition		
Non-hydrogen atoms	28089	12362
Protein residues	3358	1505
Nucleotides (RNA)	47	9
Ligands	2	1
B factors ($Å^2$)		
Protein	111.70	81.81
Nucleotide (RNA)	90.53	90.53
Ligand	93.90	96.12
R.m.s. deviations		
Bond lengths (Å)	0.002	0.003
Bond angles (°)	0.524	0.534
**Validation**		
MolProbity score	1.73	1.66
Clash score	8.34	8.28
Poor rotamers (%)	0	0
Ramachandran plot		
Favored (%)	95.95	96.64
Allowed (%)	4.05	3.36
Disallowed (%)	0	0
