# Supplementary material for: Cryo-EM structures of Thogoto virus polymerase reveal unique RNA transcription and replication mechanisms among orthomyxoviruses
Source: Nat Commun. 2024 May 30;15:4620. doi: 10.1038/s41467-024-48848-3 (PMC11139864; doi:10.1038/s41467-024-48848-3)
Supplement: Supplementary file 3 — Reporting Summary [file 41467_2024_48848_MOESM3_ESM.pdf]

Corresponding author(s): Xinwen Chen, Xiaoli Xiong

Last updated by author(s): Apr 24, 2024

Reporting Summary

Nature Portfolio wishes to improve the reproducibility of the work that we publish. This form provides structure for consistency and transparency in reporting. For further information on Nature Portfolio policies, see our Editorial Policies and the Editorial Policy Checklist.

Statistics

For all statistical analyses, confirm that the following items are present in the figure legend, table legend, main text, or Methods section.

n/a	Confirmed	
☐	☒	The exact sample size (*n*) for each experimental group/condition, given as a discrete number and unit of measurement
☐	☒	A statement on whether measurements were taken from distinct samples or whether the same sample was measured repeatedly
☐	☒	The statistical test(s) used AND whether they are one- or two-sided<br>*Only common tests should be described solely by name; describe more complex techniques in the Methods section.*
☒	☐	A description of all covariates tested
☒	☐	A description of any assumptions or corrections, such as tests of normality and adjustment for multiple comparisons
☐	☒	A full description of the statistical parameters including central tendency (e.g. means) or other basic estimates (e.g. regression coefficient) AND variation (e.g. standard deviation) or associated estimates of uncertainty (e.g. confidence intervals)
☐	☒	For null hypothesis testing, the test statistic (e.g. *F*, *t*, *r*) with confidence intervals, effect sizes, degrees of freedom and *P* value noted<br>*Give P values as exact values whenever suitable.*
☒	☐	For Bayesian analysis, information on the choice of priors and Markov chain Monte Carlo settings
☒	☐	For hierarchical and complex designs, identification of the appropriate level for tests and full reporting of outcomes
☒	☐	Estimates of effect sizes (e.g. Cohen's *d*, Pearson's *r*), indicating how they were calculated

Our web collection on statistics for biologists contains articles on many of the points above.

Software and code

Policy information about availability of computer code

Data collection	EPU automated image acquisition software
Data analysis	CryoEM data processing (all available and referenced in methods): RELION v4.0; cryoSPARC v4.3.<br>Modelling/structure refinement/visualization (all available and referenced in methods): Coot v0.9.8.1; PHENIX v1.20.1; UCSF Chimera v1.16; UCSF ChimeraX v1.4.<br>Cell assay data analysis: Graphpad Prism v9.0.

For manuscripts utilizing custom algorithms or software that are central to the research but not yet described in published literature, software must be made available to editors and reviewers. We strongly encourage code deposition in a community repository (e.g. GitHub). See the Nature Portfolio guidelines for submitting code & software for further information.

Data

Policy information about availability of data

All manuscripts must include a data availability statement. This statement should provide the following information, where applicable:

- Accession codes, unique identifiers, or web links for publicly available datasets
- A description of any restrictions on data availability
- For clinical datasets or third party data, please ensure that the statement adheres to our policy

Cryo-EM density maps and coordinates generated in this study have been deposited in the Electron Microscopy Data Bank (EMDB, https://www.ebi.ac.uk/emdb/)

and the Protein Data Bank (PDB, https://www.rcsb.org/) with the following accession numbers: THOVPol transcription pre-initiation conformation 1: EMD-39838 and 8Z85; THOVPol transcription pre-initiation conformation 2: EMD-39848 and 8Z8J; THOVPol transcription pre-initiation conformation 3: EMD-39849 and 8Z8N; THOVPol transcription initiation conformation: EMD-39850 and 8Z8X; THOVPol transcription initiation conformation 2: EMD-39852 and 8Z90; THOVPol transcription elongation conformation: EMD-39855 and 8Z97; THOVPol transcription reception conformation: EMD-39856 and 8Z98; THOVPol transcription elongation-reception conformation: EMD-39862 and 8Z9H; THOVPol replication reception conformation: EMD-39867 and 8Z9Q; THOVPol replication elongation-reception conformation: EMD-39868 and 8Z9R. Source data are provided with this paper.

Research involving human participants, their data, or biological material

Policy information about studies with human participants or human data. See also policy information about sex, gender (identity/presentation), and sexual orientation and race, ethnicity and racism.

Reporting on sex and gender	Not applicable
Reporting on race, ethnicity, or other socially relevant groupings	Not applicable
Population characteristics	Not applicable
Recruitment	Not applicable
Ethics oversight	Not applicable

Note that full information on the approval of the study protocol must also be provided in the manuscript.

Field-specific reporting

Please select the one below that is the best fit for your research. If you are not sure, read the appropriate sections before making your selection.

☒ Life sciences ☐ Behavioural & social sciences ☐ Ecological, evolutionary & environmental sciences

For a reference copy of the document with all sections, see nature.com/documents/nr-reporting-summary-flat.pdf

Life sciences study design

All studies must disclose on these points even when the disclosure is negative.

Sample size	No sample size calculation was performed as this study does not involve human or animal subjects.
Data exclusions	No data were excluded.
Replication	All experiments were performed with appropriate replicates, as described in the figure legends and Methods section.
Randomization	Not applicable or relevant, as no human or animal subjects were used in this study.
Blinding	Blinding was not necessary in this study since no subjective allocation was involved.

Reporting for specific materials, systems and methods

We require information from authors about some types of materials, experimental systems and methods used in many studies. Here, indicate whether each material, system or method listed is relevant to your study. If you are not sure if a list item applies to your research, read the appropriate section before selecting a response.

Materials & experimental systems

n/a	Involved in the study	
☐	☒	Antibodies
☐	☒	Eukaryotic cell lines
☒	☐	Palaeontology and archaeology
☒	☐	Animals and other organisms
☒	☐	Clinical data
☒	☐	Dual use research of concern
☒	☐	Plants

Methods

n/a	Involved in the study	
☒	☐	ChIP-seq
☒	☐	Flow cytometry
☒	☐	MRI-based neuroimaging

Antibodies

Antibodies used	Mouse anti-HA-monoclonal antibody (1:2000 dilution, Sino Biological, 100028-MM10).<br>HRP-labeled Goat Anti-Rabbit IgG(H+L) (Beyotime, cat. A0208, dilution 1:2000).<br>HRP-labeled Goat Anti-Mouse IgG(H+L) (Beyotime, cat. A0216, dilution 1:2000).
Validation	All antibodies are commercially available and validated by the manufacturers or by refereed articles cited on each supplier's website. The information are included in the Methods section.

Eukaryotic cell lines

Policy information about cell lines and Sex and Gender in Research

Cell line source(s)	Human embryonic kidney 293T cells (ATCC CRL-3216) were purchased from ATCC. ANP32A, B and E triple-knockout human 293T cells (TKO cells) were generated by coauthor Prof. Xiaojun Wang's group.<br>Sf9 insect cells were from coauthor Dr. Jun. He's group.
Authentication	All cell lines were frequently checked for growth rates, cellular morphologies and functions, but none of cell lines were authenticated.
Mycoplasma contamination	Mycoplasma testing revealed no contamination.
Commonly misidentified lines (See ICLAC register)	No commonly misidentified cell lines were used.

Plants

Seed stocks	Not applicable in this study.
Novel plant genotypes	Not applicable in this study.
Authentication	Not applicable in this study.
