# Supplementary material for: Cryo-EM structures of Thogoto virus polymerase reveal unique RNA transcription and replication mechanisms among orthomyxoviruses
Source: Nat Commun. 2024 May 30;15:4620. doi: 10.1038/s41467-024-48848-3 (PMC11139864; doi:10.1038/s41467-024-48848-3)
Supplement: Supplementary file 2 — Peer Review File [file 41467_2024_48848_MOESM2_ESM.pdf]

nature portfolio

Peer Review File

Cryo-EM structures of Thogoto virus polymerase reveal unique RNA transcription and replication mechanisms among orthomyxoviruses

Open Access This file is licensed under a Creative Commons Attribution 4.0 International License, which permits use, sharing, adaptation, distribution and reproduction in any medium or format, as long as you give appropriate credit to the original author(s) and the source, provide a link to the Creative Commons license, and indicate if changes were made. In the cases where the authors are anonymous, such as is the case for the reports of anonymous peer reviewers, author attribution should be to 'Anonymous Referee' followed by a clear attribution to the source work. The images or other third party material in this file are included in the article's Creative Commons license, unless indicated otherwise in a credit line to the material. If material is not included in the article's Creative Commons license and your intended use is not permitted by statutory regulation or exceeds the permitted use, you will need to obtain permission directly from the copyright holder. To view a copy of this license, visit http://creativecommons.org/licenses/by/4.0/.

REVIEWER COMMENTS

Reviewer #1 (Remarks to the Author):

Xue et al. reported ~10 cryo-EM structures for multiple conformations of THOV polymerase in different transcriptional and replicative states, including the pre-initiation, initiation, and elongation states. They used abundant structural information to describe and discuss the mechanisms concerning how the RNA template can be bound, introduced to the catalytic site, transcribed, and released. Unlike other Flu polymerases in the same virus family, Orthomyxoviridae, the absence of a cap-snatching mechanism in mRNA synthesis initiation with the THOV polymerase was proposed and discussed based on structural and biochemical analysis. One highlight is that the authors reported for the first time on a distinct asymmetric dimer conformation in transcriptional and replicative elongation states and also found one monomer is responsible for receiving the RNA product while another one works on the synthesis. Finally, they showed that the RNA synthesis mechanism of the THOV polymerase is independent of the host factor ANP32A/B, which was found to interact with the FluC polymerase dimer.

This is nice work in the negative strand RNA virus field. Some major and minor comments are listed below:
1. The most important issue with this manuscript is the presentation of the data and the body text: the figures can be improved to convey the author's information better: for example, for structure comparison, it is better to put different conformations into a single figure: Line 236 and others; legend for some figures is too small;
2. The language used can be more concise and accurate; for instance, in the method part, the cryo-EM image processing part included some repeats, and inconsistency was found in many places, including the number of particles in line 909 is not the same as one in fig S4, please ensure accuracy throughout the whole manuscript.
3. Another question is about the preparation of THOV polymerase in different states: that authors may explain the reason for the mixtures and conditions or provide references for readers to believe their definitions of the multiple conformations, for example, why the elongation states samples require a 6 hrs incubation at 30 degrees? Does $MnCl_2$ make a difference than $MgCl_2$?

Minor comments:
1. An English language editing on the current version of the manuscript is recommended so that readers can clearly receive information from the authors.
2. Please explain the sources and category number for all the materials used.
3. Please explain "DKO" in the first places it came.
4. Line 888, the generation of the initial model by cryoSPARC should be described.
5. Please explain "VDW" and "BRBV" in the first places they appeared.
6. Fig 1d, the stars may not be used to cover the image.
7. Please unify the usage of 5'- and 3'-vRNA in lines 850 and 855. Please confirm if only one 5'- and one 3'-vRNA were used in this study.

Reviewer #2 (Remarks to the Author):

Like the influenza viruses (Flu), Thogotovirus (THOV) is a member of the family Orthomyxoviridae, a segmented negative-sense RNA virus. Xue et al presented different conformations of the THOV polymerase in the transcriptional pre-initiation, initiation, and elongation states, as well as the replicative elongation states. Informative details concerning RNA template entry, synthesis, and exit as well as RNA product formation and exit are provided or discussed. Structural and biochemical analysis on the reason why THOV polymerase may not have a cap-snatching mechanism for mRNA synthesis initiation as other Flu polymerases was provided. The authors further discovered RNA-synthesis-mediated asymmetric homo-dimer conformations of THOV during the transcriptional and replicative

elongation states, which are distinct from the FluC polymerase dimer structure containing the ANP32A protein that is a host factor. They demonstrated a host factor ANP32A/B independent RNA synthesis mechanism of the THOV polymerase by cell-based assays. This nice study is of immediate interest to the RNA virus field and would deepen the understanding of the RNA synthesis mechanism and host infectivity range of the Orthomyxoviridae, however, before consideration for publication I have 2 major comments on its flaws and some minor comments as listed below:

Major ones:

1. The methods part should be revised to enable reproducing the results:

1) please provide sufficient information and description of all the used materials, for instances: cat No. in lines 730, 767, 775, 807, and 835, etc; line 711, please explain the "DKO" and its source; line 725, after this line, protein expression details should be described; line 730, please clarify if wash before elution was done; line 816, please explain "loading buffer"; line 884, please provide a reference for the "Gaussian filter";

2) please revise the Cryo-EM image processing part:

a. line 884-886, the number in line 885 and description are not consistent with the figure S1, please clarify which is the right one.

b. line 888, and line 911, please describe how the initial model was generated by cryoSPARC.

c. Line 892-893, 3′ vRNA was not indicated in any cryo-EM map in fig S1a.

d. Line 909, the number of particles is not consistent with the one in fig S4, please clarify which is the right one.

e. Fig S4a, please explain how the duplex RNA can be identified.

2. Quality of presentation and context can be improved:

1) The introduction part seems to be not enough, while content in lines 119-131 may be integrated into the introduction part.

2) Figures containing multiple domains in different colors can be improved: in fig2a, 3 different regions of PB2 other than the cap-binding domain and linker may be labeled with different colors; captions are too small for readers in some figures including 2e, 3b, s3f, s4c, and s7h, for example, interactions mentioned in line 170 are hard to view, and it seems difficult for readers to do the comparison in line 236.

3) Please proofread the manuscript to avoid any errors and confusion, for example, in line 185 and 188, the R305 and R309 should be wrong; line 193, please specify "see below"; line 234, fig. 4e should be 3e; line 352, "576-585" is not consistent with fig 5c; line 459-463, description is not consistent with the figure;

4) A professional English language editing is advisable: for example, line 306-307; line 236-238, it is hard to follow.

Minor comments:

1. Line 148, more introduction content is needed for "segment 4"
2. Line 167, please add a reference after the "FluPols"
3. Line 258, please explain "VDW"
4. Line 279, "sequences" should be changed to "residues"
5. Line 407, please explain what is the "interaction" mentioned here?
6. Line 444, please explain why the activity is abrogated but 100 times that of background.
7. Line 471, please explain "BRBV"
8. Line 855 and 859, please explain why different concentrations of 3′/5′ vRNA were used for transcriptional and replicative elongation samples;
9. Fig 1d, a gel image with a clear background is preferred
10. Fig S3d, the red cross symbol may be too big for comparison with S3a

Reviewer #3 (Remarks to the Author):

This manuscript presents a lot of structural information about the Thogoto virus polymerase. In that respect it is quite novel and will be of significant interest to virologists and structural biologists. Thogoto virus is an orthomyxovirus related to influenza viruses, about which there is already a lot of

structural information. For example, a number of papers have shown that the trimeric influenza polymerase can exist in different forms as monomers or dimers or even higher order forms. Switching between these forms may be driven by the polymerase's interaction with different factors from virus or host.
This manuscript presents some tantalizing differences.. for example it suggests that the transcriptase form of Thogoto virus polymerase exists as a dimer. It also suggests that the asymmetric dimer does not require the host factor ANP32 to form.

If these assertions are validated they will be of great interest to the community.
However there is very little biological validation provided in this manuscript, and a number of other caveats mean that as it stands the highly controversial claims are too poorly supported.

In figure 1 the authors confirm that Thogoto virus transition is indeed somewhat different from influenza transcription because the product does not contain any host derived nucleotides downstream from the cap- in other words thee is either no cap snatching or cap snatching must work very differently.
The data for this is in figure 1a but is hardly explained at all. It involves the use of a minireplicon system with Thogoto virus polymerase compared to that of influenza A virus. The readout of such systems is usually a luciferase assay but this is not provided and therefore it is very difficult to know how efficient the Thogoto virus polymerase is in the human cells used.
A more expanded explanation of these data is required.

The second part of figure 1 demonstrates that the trimeric Thogoto virus polymerase expressed in insect cells functions as an RNA dependent RNA polymerase. In figure 1b there is a large amount of contaminating/ co-purifying RNA that authors do not comment. In figure 1c and d two different primers are used for the in vitro reaction, and it is assumed that the products of the RdRp with capped primer are true mRNA transcripts whereas the products of the RdRp from an uncapped primers are replication products. It is not clear from the data provided that this is the case… and this is important because later in the manuscript the polymerase is 'frozen' in conjunction with one or other primers and then because a dimer is found in conjunction with the capped primer it is claimed that transcription proceeds via the dimer.
It is difficult to understand how orthomyxovirus transcription would work if a dimer is needed, since the incoming virus only has the cis-acting polymerase so the primary transcript must be performed by a monomer.. what then is the difference for secondary transcription and why is the dimer needed there?
The obvious alternative explanation is that the assays they have used do not distinguish between the transcription and replication mode of polymerase, ie the dimers they see are replicases that can happen to use a short capped primer to initiate as well as an uncapped one. The authors should address this as it is central to their claims.

The only real biological validation of their structures is presented in figure 6 g and h, when the authors engineer mutations to the Thogoto virus polymerase and perform minireplicaon assays. Mutations deigned to interfere with asymmetric dimers formation decrease the luciferase signal and this is due to decreases in all 3 species of viral RNA v,m and c. The authors claim this indicates that both replication AND transcription are affected by the dimeric form of polymerases, however, the mRNA produced in these assays is absolutely dependent on the amplified signal from replication. So a decrease in mRNA is exactly what is expected if replication is decreased. In the text the authors refer to 2 previous papers that have disrupted the influenza virus polymerase dimeric interface and seen bigger impacts on cRNA than mRNA. However it is not appropriate to compare these ratios across difference viruses using different techniques. The authors will need to develop a biological assay that reads out transcription only. Such assays do exist in the influenza field, such as treating cells with cycloheximide to inhibit protein translation prior to infection, allowing primary transcription but no viral replication to occur due to the lack of complementing polymerases and NP for nascent RNPs. It might be interesting to ask whether preexpressed polymerase affects transcription of Thogoto virus in such an assay.

However, establishing new assays like this will not be easy and the authors may instead need to tone down their claims that are currently based on structure alone.
Another large part of their story is that the Thogoto replicase (asymmetric dimer) is independent of host factor ANP32.
Here they present some functional assays in Figure 6. However they perform these assays in human cells that lack human ANP32A and ANP32B. Others have recently shown that where the interaction between influenza polymerase symmetric dimers is less strong, other ANP32 family members such as ANP32E can be used instead. At the very least the authors should perform these assays in triple knockout cells.
In addition since thogoto virus is not a natural virus of humans, the authors should also assessing whether the addition of ANP32 proteins cloned from different more relevant species such as ticks or mice might further enhance the replication activity they observe- the small but significant enhancement they see in presence of human ANP32B might be much greater if a better species matched host protein were used.
It would also be useful to show the raw data so that the absolute levels of polymerase activity can be compared in these cells between Thogoto virus and IAV polymerase.

In summary the structural data here are very interesting but it is not clear whether the dimeric forms of polymerase described are biologically relevant in the way the authors suggest and the biological validation is currently lacking. To have such a controversial piece of work accepted in the field the authors will need to develop better biological assays to validate their findings.

REVIEWER COMMENTS AND RESPONSES

Reviewer #1 (Remarks to the Author):

Xue et al. reported ~10 cryo-EM structures for multiple conformations of THOV polymerase in different transcriptional and replicative states, including the pre-initiation, initiation, and elongation states. They used abundant structural information to describe and discuss the mechanisms concerning how the RNA template can be bound, introduced to the catalytic site, transcribed, and released. Unlike other Flu polymerases in the same virus family, Orthomyxoviridae, the absence of a cap-snatching mechanism in mRNA synthesis initiation with the THOV polymerase was proposed and discussed based on structural and biochemical analysis. One highlight is that the authors reported for the first time on a distinct asymmetric dimer conformation in transcriptional and replicative elongation states and also found one monomer is responsible for receiving the RNA product while another one works on the synthesis. Finally, they showed that the RNA synthesis mechanism of the THOV polymerase is independent of the host factor ANP32A/B, which was found to interact with the FluC polymerase dimer.
This is nice work in the negative strand RNA virus field. Some major and minor comments are listed below:

Response:

We thank the reviewer for his/her positive comments. We have revised the manuscript according to the comments listed below:

1. The most important issue with this manuscript is the presentation of the data and the body text: the figures can be improved to convey the author's information better: for example, for structure comparison, it is better to put different conformations into a single figure: Line 236 and others; legend for some figures is too small;

Response:

We greatly appreciate the reviewer's suggestions. We checked the whole manuscript carefully and updated the figures. We now present a new figure (**Fig. S8)** showing all the conformations involved in cross-figure comparison mentioned in the manuscript for ease of comparison. We have enlarged the panels in **Figs.2-4** and **Fig. S7** to annotate the Figures with larger captions.

2. The language used can be more concise and accurate; for instance, in the method part, the cryo-EM image processing part included some repeats, and inconsistency was found in many places, including the

number of particles in line 909 is not the same as one in fig S4, please ensure accuracy throughout the whole manuscript.

Response:

We thank the reviewer for pointing out this issues in our manuscript. We have rewritten the cryo-EM method section to be more concise and to reduce repeats. We have corrected the inconsistency regarding the particle number after 2D classification for the "transcriptional elongation sample" in the "Method-Cryo-EM image processing" section (Line 1527 of the track-change MS). Now, the number (10.1 million) matches the number in **Fig.S7** in the new manuscript. We thank the reviewer for his/her careful check on the manuscript again!

3. Another question is about the preparation of THOV polymerase in different states: that authors may explain the reason for the mixtures and conditions or provide references for readers to believe their definitions of the multiple conformations, for example, why the elongation states samples require a 6 hrs incubation at 30 degrees? Does MnCl2 make a difference than MgCl2?

Response:

The previously reported conditions for the influenza A and B polymerase elongation reactions were 28 °C, 5 h, 5 mM $MgCl_2$ (Wandzik *et al*, 2020) and 30 °C, 5 h, 5 mM $MgCl_2$ (Kouba *et al*, 2019) respectively. The two conditions are similar, therefore, to prepare the sample for the THOVPol elongation states, we initially attempted the reported condition for influenza B polymerase (30 °C, 5 h, 5 mM $MgCl_2$). Unfortunately, under such condition, the polymerase was not active, and the expected product bands could not be observed. We simply substituted the 5 mM $MgCl_2$ with 2 mM $MnCl_2$ and extended the reaction time by 1h; and we were able to obtain the anticipated result, which is shown in **Fig.1d**. Cryo-EM samples giving different THOVPol elongation conformations were based on the primer extension results.

We have added the references for influenza A and B polymerase elongation reactions in the Method section and explained that our conditions were developed based on those reported conditions in the "Methods-Cap-dependent transcription activity assay" (Line 1317 of tracked-change MS) and "Methods- Cryo-EM sample preparation and data collection" (Line 1426 of tracked-change MS).

Minor comments:

1. An English language editing on the current version of the manuscript is recommended so that readers can clearly receive information from the authors.

Response:

We thank the reviewer for pointing out the language issues. We have proof-read the whole manuscript carefully and corrected/rephrased sentences where needed. Modifications are shown in the track-change MS.

2. Please explain the sources and category number for all the materials used.

Response:

We apologize for the missing information. We have ensured that manufacture information and catalogue numbers are added to the materials used through out the revised manuscript.

3. Please explain "DKO" in the first places it came.

Response:

We apologize for the confusion. Due to extra experiments suggested by reviewer 3, we now use "TKO" cells which are ANP32A/B/E triple knock-out cells. We have added the explanation (ANP32A/B/E triple knock-out human 293T cells) and the source "generated by coauthor Prof. Xiaojun Wang's group in a previous study (Zhang et al, 2020)" for the "TKO" cells in the revised manuscript in the "Methods-Cells" section (Line 1211 of tracked-change MS).

4. Line 888, the generation of the initial model by cryoSPARC should be described.

Response:

Generation of the initial models was performed using a simple procedure in cryoSPARC. We blob picked particle in cryoSPARC. Particles with good features, selected by 2D classification, were used for initial model generation using standard settings in cryoSPARC. We added further descriptions in the "Method- Cryo-EM image processing" section (Line 1471/1517 of tracked-change MS) and included a new **Fig.S14** to illustrate the procedures.

5. Please explain "VDW" and "BRBV" in the first places they appeared.

Response:

We have added the explanations for "VDW" (Van der Waals) and "BRBV" (Bourbon virus) in the revised manuscript.

6. Fig 1d, the stars may not be used to cover the image.

Response:

We thank the reviewer for this suggestion. We have moved the necessary symbols to an empty lane or outside of the gel (revised **Fig.1d**). We will also provide an unedited original gel image as Source Data as required by the journal.

7. Please unify the usage of 5′- and 3′-vRNA in lines 850 and 855. Please confirm if only one 5′- and one 3′-vRNA were used in this study.

Response:

We thank the reviewer for pointing out this issue. We have removed the hyphens and unified the usage as "5' vRNA" and "3' vRNA" throughout the revised manuscript. In the study, we only utilized one type of 5' vRNA and one type of 3' vRNA.

Reviewer #2 (Remarks to the Author):

Like the influenza viruses (Flu), Thogotovirus (THOV) is a member of the family Orthomyxoviridae, a segmented negative-sense RNA virus. Xue et al presented different conformations of the THOV polymerase in the transcriptional pre-initiation, initiation, and elongation states, as well as the replicative elongation states. Informative details concerning RNA template entry, synthesis, and exit as well as RNA product formation and exit are provided or discussed. Structural and biochemical analysis on the reason why THOV polymerase may not have a cap-snatching mechanism for mRNA synthesis initiation as other Flu polymerases was provided. The authors further discovered RNA-synthesis-mediated asymmetric homo-dimer conformations of THOV during the transcriptional and replicative elongation states, which are distinct from the FluC polymerase dimer structure containing the ANP32A protein that is a host factor. They demonstrated a host factor ANP32A/B independent RNA synthesis mechanism of the THOV polymerase by cell-based assays. This nice study is of immediate interest to the RNA virus field and would deepen the understanding of the RNA synthesis mechanism and host infectivity range of the Orthomyxoviridae, however, before consideration for publication I have 2 major comments on its flaws and some minor comments as listed below:

Response:

We thank the reviewer for the positive comments and we have revised the manuscript according to the points raised.

Major ones:

1. The methods part should be revised to enable reproducing the results:

1) please provide sufficient information and description of all the used materials, for instances: cat No. in lines 730, 767, 775, 807, and 835, etc; line 711,

Response:

We thank the reviewer for pointing out these issues. We now provide manufacture names and catelogue numbers for all the reagents used in this manuscript.

please explain the "DKO" and its source;

Response:

Due to extra experiments suggested by reviewer 3, we now use "TKO" cells which are ANP32A/B/E triple knock-out cells instead of the "DKO" ANP32A/B double knock-out cells. We have added the explanation (ANP32A/B/E triple knock-out human 293T cells) and the source (generated by coauthor Prof. Xiaojun Wang's group in a previous study (Zhang *et al.*, 2020)) in the "Methods-Cells" section (Line 1210 of tracked-

change MS).

line 725, after this line, protein expression details should be described;

Response:

We have included more details on protein expression in the "Method-Protein expression and purification" section (Line 1220 of tracked-change MS), including plasmid construction, recombinant-baculovirus generation, sf9 cell culture condition etc.

line 730, please clarify if wash before elution was done;

Response:

We have added a sentence to describe that the resin incubated with cleared lysate was washed twice with wash buffer A before elution in the "Method-Protein expression and purification" section (Line 1260 of tracked-change MS).

line 816, please explain "loading buffer";

Response:

We have added more details about the "loading buffer" as "5x protein loading buffer (Solarbio, P1040)" in the "Methods-Western blotting" section (Line 1384 of tracked-change MS).

line 884, please provide a reference for the "Gaussian filter";

We have supplied the RELION 3.0 reference (Zivanov *et al*, 2018) for the "Gaussian filter" which was introduced in RELION 3.0 in the "Method- Cryo-EM image processing" section (Line 1467 of tracked-change MS).

2) please revise the Cryo-EM image processing part:

a. line 884-886, the number in line 885 and description are not consistent with the figure S1, please clarify which is the right one.

Response:

We apologize for the mistake. We blob picked 100 micrographs and used 2D-classfication to select good featured particles (40,969 particles) from the 100 micrographs. The 40,969 good particles were used for training Topaz, which subsequently picked 9,889,120 particles from all the micrographs. Following 2D classification, well-featured particles (5,457,133 particles) were used for further data processing. We apologize for any confusion caused, and we have modified **Fig.S2** (**Fig.S1** in the previous manuscript) and updated the "Method-Cryo-EM image processing" section (Line 1468 of tracked-change MS) to rectify the errors.

b. line 888, and line 911, please describe how the initial model was generated by cryoSPARC.

Response:

We added further details in the "Method-Cryo-EM image processing" section (Line 1472 and 1517 of tracked-change MS) and included **Fig.S14** to describe the procedures to obtain initial models used for 3D classification in RELION. Briefly, the initial models were generated in cryoSPARC for both the transcriptional initiation and the transcriptional elongation datasets. We blob picked particles in cryoSPARC. Using standard settings, well featured particles identified by 2D classification were used to generate initial models in cryoSPARC.

c. Line 892-893, 3' vRNA was not indicated in any cryo-EM map in fig S1a.

Response:

We thank the reviewer for raising this point. **Fig.S2a** (**Fig.S1a** in old MS) has been revised to outline the densities for the 3' vRNA (regions within red-dashed lines) as shown below:

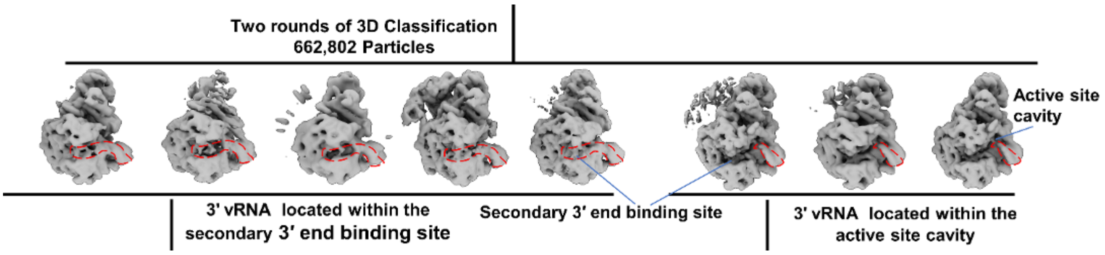

As can be seen from the above illustrations, the 3' vRNAs adopt 2 distinct trajectories when bound to the secondary 3' end binding site or when extended into the active site cavity. Based on the above features, particles were pooled and further processed.

d. Line 909, the number of particles is not consistent with the one in fig S4, please clarify which is the right one.

Response:

We apologize for this mistake. The correct number of particles is 10,100,652. We have corrected it in the "Method-Cryo-EM image processing" section (line 1516of the track-change MS) as "10.1 million particles".

e. Fig S4a, please explain how the duplex RNA can be identified.

Response:

We used cut-away views to identify whether the duplex RNA is present or not within the polymerase active site cavity as shown below:

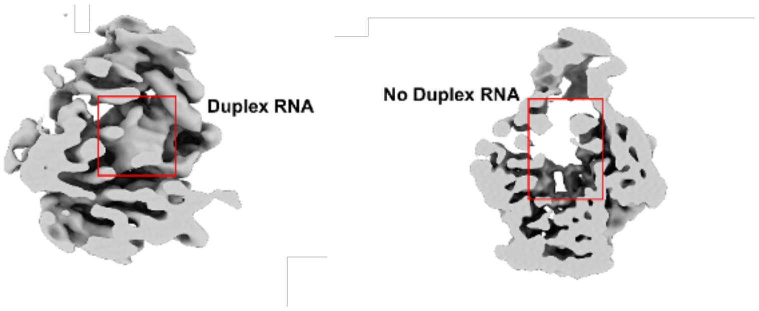

We have updated **Fig.S7** (**Fig.S4** in the old MS) with the above illustrations. In the updated **Fig.S7,** the class on the leftmost side features duplex RNA density within the polymerase active site cavity. In contrast, the class on the rightmost side has an empty polymerase active site cavity.

2. Quality of presentation and context can be improved:

1) The introduction part seems to be not enough, while content in lines 119-131 may be integrated into the introduction part.

Response:

We thank the reviewer for this suggestion. We have expanded the Introduction section (Line 58 of the track-change MS) to include extra information about cap-snatching in flu viruses and unique features found for THOV mRNA in the revised manuscript.

2) Figures containing multiple domains in different colors can be improved: in fig2a, 3 different regions of PB2 other than the cap-binding domain and linker may be labeled with different colors; captions are too small for readers in some figures including 2e, 3b, s3f, s4c, and s7h, for example, interactions mentioned in line 170 are hard to view, and it seems difficult for readers to do the comparison in line 236.

Response:

We appreciate the suggestions provided by the reviewer. **Fig2a** has been updated, 3 different colors are now used to represent the 3 different PB2 regions. The captions for **Figs.2e, 3b, S4f** (**s3f** in the old MS) in the revised MS), **S7c** (**S4c** in the old MS) and **S10h** (**S7h** in the old MS) have been enlarged; The detailed interactions (as mentioned in Line 458 in the track-change MS) between the PA handle structure and the $PB1^{353-364}$ β-hairpin are now shown in a new Figure panel (**Fig.S5a** in the revised MS**)**. Finally, we have compiled structures relating to transcription initiation and transcription elongation (which are compared cross figures) into a new Figure (**Fig.S8** in the revised MS) to facilitate better comparison.

3) Please proofread the manuscript to avoid any errors and confusion, for example, in line 185 and 188, the R305 and R309 should be wrong; line 193, please specify "see below"; line 234,

fig. 4e should be 3e; line 352, "576-585" is not consistent with fig 5c; line 459-463, description is not consistent with the figure;

Response:

We apologize for the errors. The R305 and R309 should be K305 and K309, and we have corrected them in the text (Line 480-483 in the track-change MS); In line 193 of previous MS, the phrase "see below" has been revised to "see below, **Fig. 4f-g**" (Line 488 in the track-change MS); "fig. 4e" has been amended to "**Fig.3e**" (Line 525 in the track-change MS); We have unified the text as "$PB1^{576\text{-}585}$" (Line 716 in the track-change MS, Line 353 in the previous MS) to ensure its consistency with the representation in **Fig.5c**; We have revised the description (Line 880-883 in the track-change MS; Line 459-463 in old MS)

"*the distance (40 Å), between the active sites of the putative cap-snatching domains, is smaller than that between the active sites of the functional cap-snatching domains of FluAPol (56 Å). However, this distance is still substantial and thus incompatible with the suggestion that the putative THOVPol cap-snatching domains may provide short capped primers for THOV transcription.*"

to ensure its consistency with the representation in **Fig.S13**.

4) A professional English language editing is advisable: for example, line 306-307; line 236-238, it is hard to follow.

Response:

We thank the reviewer for pointing out the language issues. We have proof-read the whole manuscript and made necessary changes. The mentioned sentences (Line 306-307; Line 236-238, in previous MS) have been modified for better clarity (Line 650-652; Line 551-557 in the track-change MS)

Minor comments:

1. Line 148, more introduction content is needed for "segment 4"

Response:

We greatly appreciate the reviewer's suggestion. Being an orthomyxovirus, THOV's genome comprises six negative-sense single-stranded RNA segments. Segment 4 of THOV encodes the glycoprotein. We have added more info about THOV genome in the "Introduction" section (Line 77 in the track-change MS).

2. Line 167, please add a reference after the "FluPols"

Response:

We appreciate the reviewer for bringing this to our attention. 4 references (Carrique *et al*, 2020; Kouba *et al.*, 2019; Peng *et al*, 2019; Wandzik *et al.*, 2020) are now cited for the "5′ promoter binding sites of FluPols".

3. Line 258, please explain "VDW"

Response

We have added the explanation (Van der Waals) for "VDW" in the revised manuscript.

4. Line 279, "sequences" should be changed to "residues"

Response:

We have corrected the text accordingly.

5. Line 407, please explain what is the "interaction" mentioned here?

Response:

We have amended the text in the revised manuscript to clarify that "interaction" refers to the interactions to form the asymmetric dimer". The revised text is shown as follows:

"*Due to the absence of the handle-helix, FluCPol$_{EN}$ 5' promoter binding site structural elements (ie. PA^{346}-PA^{363}) is not involved in the interaction forming the asymmetric FluCPol dimer (**Fig.6e and f**)*" (Line 777-779 of the track-change MS).

6. Line 444, please explain why the activity is abrogated but 100 times that of background.

Response:

According to reviewer 3's suggestions, we have updated the activity experiment with TKO cells (ANP32A/B/E triple knock-out human 293T cells), the results remain similar to the previous DKO cell result. The assay is a luciferase assay and is sensitive enough to detect small amount of luciferase produced by the mini-replicon system. We used relative activity units in our experiment for ease of comparison. We now define the activity of THOVPol as 100x. IAV Pol now has an activity of ~2x, the background has an activity of 0.2x. So IAV Pol in TKO cell still has 10 times higher activity than the background. Therefore, IAV Pol mini-replicon in ANP32 TKO cells still has trace activity probably due to remaining transcription activity. However, the activity is very low due to that the activity can not be amplified by the polymerase replication activity which is heavily dependent on ANP32. Great amplification by the polymerase replication activity can be observed after ANP32 complementation, IAV Pol activity can be as high as 10,000x (increase by 5000x) after ANP32A or ANP32B complementation. Therefore, the activity of IAV Pol is greatly diminished but probably not completely abolished in the ANP32 TKO cells. We have changed the word from "abrogated" to "reduced" in the revised manuscript (Line 833 of track-change MS).

7. Line 471, please explain "BRBV"

Response:

We have added the explanation (Bourbon virus) for "BRBV" in the revised manuscript.

8. Line 855 and 859, please explain why different concentrations of 3'/5' vRNA were used for transcriptional and replicative elongation samples;

Response:

We initially prepared the transcriptional elongation sample. We identified both the presence of asymmetric dimers and monomers upon cryo-EM data analysis. In the asymmetric dimer, one monomer is in a transcription elongation conformation while the other serves as a recipient to bind the synthesized RNA product. This result suggests that THOVPol undergoing RNA synthesis can recruit a free “apo” polymerase for product reception. Hence, to optimize the sample for the replication elongation asymmetric dimer, we decreased the vRNA concentrations to increase the amount of free polymerases available for product reception.

9. Fig 1d, a gel image with a clear background is preferred

Response:

We thank the reviewer for this suggestion. We have moved the necessary symbols to an empty lane or outside of the gel. We will also provide an unedited image as Source Data as required by the journal.

10. Fig S3d, the red cross symbol may be too big for comparison with S3a

Response:

We appreciate the reviewer for bringing this to our attention. **Fig. S4d** (**Fig. S3d** in the old MS) has been revised to use a circle to mark the closed template exit channel.

Reviewer #3 (Remarks to the Author):

This manuscript presents a lot of structural information about the Thogoto virus polymerase. In that respect it is quite novel and will be of significant interest to virologists and structural biologists.
Thogoto virus is an orthomyxovirus related to influenza viruses, about which there is already a lot of structural information. For example, a number of papers have shown that the trimeric influenza polymerase can exist in different forms as monomers or dimers or even higher order forms. Switching between these forms may be driven by the polymerase's interaction with different factors from virus or host.
This manuscript presents some tantalizing differences.. for example it suggests that the transcriptase form of Thogoto virus polymerase exists as a dimer. It also suggests that the asymmetric dimer does not require the host factor ANP32 to form.

If these assertions are validated they will be of great interest to the community.
However there is very little biological validation provided in this manuscript, and a number of other caveats mean that as it stands the highly controversial claims are too poorly supported.

Response:

We thank the reviewer for his/her constructive comments. We have incorporated additional biological experiments as suggested by the reviewer. Furthermore, we have made adjustments to claims that were subject to controversy.

1) In figure 1 the authors confirm that Thogoto virus transition is indeed somewhat different from influenza transcription because the product does not contain any host derived nucleotides downstream from the cap- in other words thee is either no cap snatching or cap snatching must work very differently.
The data for this is in figure 1a but is hardly explained at all. It involves the use of a minireplicon system with Thogoto virus polymerase compared to that of influenza A virus. The readout of such systems is usually a luciferase assay but this is not provided and therefore it is very difficult to know how efficient the Thogoto virus polymerase is in the human cells used.
A more expanded explanation of these data is required.

Response:

We thank the reviewer for this comment. Previously, it has been reported that mRNA of THOV is devoid of 5' heterologous sequences of 9-14 nucleotides(Albo *et al*, 1996; Koppstein *et al*, 2015). This result is very unusual, so we first attempted to confirm this

result. We constructed a mini-replicon system expressing green fluorescent protein (GFP), driven by THOVPol. For comparison, we also constructed equivalent mini-replicon systems with DHOVPol and FluAPol. Of note, similar GFP expressing mini-replicon systems were used to study polymerases of FluA and Bourbon virus of thogotovirus family (Hao *et al*, 2020; Ozawa *et al*, 2013). We added an extra figure - **Fig.S1** to shown how the 3 systems express GFP in 293T cells. **Fig.S1** shows that the 3 mini-replicon systems can all express GFP with reasonable efficiency as judged by cell fluorescence. Our sequencing results are consistent with previous reports that the 5' terminal sequences of mRNAs transcribed by THOVPol and DHOVPol are homogeneous in both length and sequence without extraneous 9-14 nucleotides, while those of influenza A comprise heterologous sequences of 9-14 nucleotides (**Fig.1a**). These results validate previous observations on THOV and we went ahead to perform our structural study.

We added more explanation about the results in **Fig.1a** in the "Results-THOV polymerase transcription and replication activity" section (Line 290 of track-change MS).

2) The second part of figure 1 demonstrates that the trimeric Thogoto virus polymerase expressed in insect cells functions as an RNA dependent RNA polymerase. In figure 1b there is a large amount of contaminating/ co-purifying RNA that authors do not comment.

Response:

We apologize for the confusion. Fig.2b shows size-exclusion chromatogram of THOV polymerase complex during protein purification. The inset shows a Coomassie-blue stained SDS-PAGE protein gel, we included the marker lane (marker bands are labeled with sizes) and the sample lane, the sample lane clearly shows only 3 bands corresponding to the correct sizes for PB2, PB1 and PA without sign of contamination. At the peak of the THOV polymerase complex size-exclusion chromatogram, the 260:280 nm absorbance ratio is approximately 0.53, which suggests that the purified protein is largely free of nucleic acid contamination (260:280 nm absorbance ratio for a typical protein at 100% purity is ~ 0.57; while 260:280 nm absorbance ratio for protein with 5% nucleic acid contamination is ~ 1.06. https://en.wikipedia.org/wiki/Nucleic_acid_quantitation).

3) In figure 1c and d two different primers are used for the in vitro reaction, and it is assumed that the products of the RdRp with capped primer are true mRNA transcripts whereas the products of the RdRp from an uncapped primers are replication products. It is not clear from the data provided that this is the case… and this is important because later in the manuscript the polymerase is 'frozen' in conjunction with one or other primers and then because a dimer is found in conjunction with the capped primer it is claimed that transcription proceeds via the dimer.

It is difficult to understand how orthomyxovirus transcription would work if a dimer is needed, since the incoming virus only has the cis-acting polymerase so the primary transcript must be performed by a monomer.. what then is the difference for secondary transcription and why is the dimer needed there?

The obvious alternative explanation is that the assays they have used do not distinguish between the transcription and replication mode of polymerase, ie the dimers they see are replicases that can happen to use a short capped primer to initiate as well as an uncapped one. The authors should address this as it is central to their claims.

Response:

The in-vitro primer extension assay referred by the reviewer has been extensively employed to trap flu polymerases in various conformations for structural study (Kouba *et al.*, 2019; Wandzik *et al.*, 2020), therefore, we adopted similar procedures for the structural study of THOVPol. To trap FluPols in transcriptional conformations, previous studies employed capped RNA segments of 10-13 nucleotides (Cap-N_{12}AG) as primers for in-vitro transcriptional elongation. This design allows the cap binding domain to bind to the 5' end of the capped RNA segment, enabling the free 3' end to probe into the polymerase active site and to base pair with the template through 1-2 nt to start RNA synthesis. The synthesized RNA contains extraneous nucleotide sequences at the 5' end and is bound to both the cap-binding domain and the polymerase active site (see one such FluPol structure trapped under transcription elongation in **Fig. S13c**) (Kouba *et al.*, 2019; Wandzik *et al.*, 2020). For THOV, it has been reported that 1. THOVPol lacks cap binding and endonuclease activity (suggesting that capped long RNA segments won't work as primers) (Guilligay *et al*, 2014); 2. that THOV mRNA lacks extraneous sequence (Koppstein *et al.*, 2015); and 3. that in vitro transcription products have been obtained using capped-mononucleotide using the THOV viral core (Leahy *et al*, 1997). Based on these existing data, we chose to use the capped-dinucleotide, in our opinion, representing a possible starting primer under physiological condition (dinucleotide is known to have higher efficiency than mononucleotide to initiate RNA synthesis for FluPol), to recapitulate THOVPol transcription elongation.

Regarding the function of the THOVPol dimer. We do not claim that a polymerase dimer is absolutely needed for the initial transcription or secondary transcript. In fact, our structural data show that THOVPol functions as monomers during transcription preinitiation and initiation. Our structural data also show that THOVPol can function as monomers during transcription process. Cryo-EM imaging of THOVPol trapped under transcriptional elongation conformation shows that a population of transcribing THOV polymerases exits as monomers despite that some THOV polymerases exist as dimers. Based on these structural data, we agree with the reviewer that "incoming virus only has the cis-acting polymerase so the primary

transcript must be performed by a monomer" and we said in our Discussion section that "*asymmetric dimerization could be modulated by the availability of free THOVPol. Such a dimerization mechanism implies that during early viral infection, THOVPol should function as a monomer when the free THOVPol concentration is low; however, a switch to a polymerase asymmetric dimer may occur when free THOVPol accumulates*." We speculate that THOVPol asymmetric dimer formation is modulated by availability of free polymerase, an idea which has also been proposed for the switch of flu polymerase activity from transcription to replication (Olson *et al*, 2010; Zhu *et al*, 2023).

Although our structures show dimers are compatible with early stage of transcriptional RNA synthesis, there is no data regarding whether THOVPol dimers are compatible with later stage of transcriptional RNA synthesis including polyadenylation. Therefore, we agree with the reviewer that it is premature to claim that THOVPol dimer is physiologically involved in transcription. We removed claims implying THOVPol dimer is involved in transcription physiologically with the following modifications to the manuscript:

The following sentences are removed from the "Asymmetric THOVPol dimer and RNA synthesis" section (Line 782-789 of track-change MS) and Discussion section (Line 903-939 in the track-change MS):

"~~*In contrast, previous studies on FluPols found that mutations affecting ANP32-mediated asymmetric dimer formation resulted in a more significant reduction in cRNA levels compared with mRNA levels. The above observations suggest that, in addition to a role in genome replication, asymmetric dimerization of THOVPol may also play a functional role in RNA transcription.*~~"

~~"*It has been proposed that abolishment of FluPol asymmetric dimer formation more strongly affects viral genome replication. Mutagenesis of the THOVPol asymmetric dimer interface, however, shows that mutations affecting asymmetric dimer formation reduce both THOVPol RNA transcription and replication to similar extents. If both THOV RNA transcription and replication extend short nucleotides, the THOVPol asymmetric dimer should form during both RNA transcription and replication. The THOVPol asymmetric dimer appears to be structurally compatible with both transcriptional and replicative RNA synthesis.*"~~

5)The only real biological validation of their structures is presented in figure 6 g and h, when the authors engineer mutations to the Thogoto virus polymerase and perform minireplicaon assays. Mutations deigned to interfere with asymmetric dimers formation decrease the luciferase signal and this is due to decreases in all 3 species of viral RNA v,m and c. The authors claim this indicates

that both replication AND transcription are affected by the dimeric form of polymerases, however, the mRNA produced in these assays is absolutely dependent on the amplified signal from replication. So a decrease in mRNA is exactly what is expected if replication is decreased. In the text the authors refer to 2 previous papers that have disrupted the influenza virus polymerase dimeric interface and seen bigger impacts on cRNA than mRNA. However it is not appropriate to compare these ratios across difference viruses using different techniques. The authors will need to develop a biological assay that reads out transcription only. Such assays do exist in the influenza field, such as treating cells with cycloheximide to inhibit protein translation prior to infection, allowing primary transcription but no viral replication to occur due to the lack of complementing polymerases and NP for nascent RNPs. It might be interesting to ask whether preexpressed polymerase affects transcription of Thogoto virus in such an assay. However, establishing new assays like this will not be easy and the authors may instead need to tone down their claims that are currently based on structure alone.

Response:

We thank the reviewer for the constructive comments. We agree with the reviewer that mRNA production in mini-replicon assay is greatly subjected to amplification by the RNA replication process mediated by the viral Pol.

The method using cycloheximide to only study transcription relies on the use of live virus, unfortunately we don't have THOV live virus at the moment and we are not sure whether live viruses with mutations disrupting the THOVPol dimers could be generated via reverse-genetics. Therefore, we remove claims that suggest dimer formations directly affect mRNA production.

We removed the sentence:

"~~*In contrast, previous studies on FluPols found that mutations affecting ANP32-mediated asymmetric dimer formation resulted in a more significant reduction in cRNA levels compared with mRNA levels18,33. The above observations suggest that, in addition to a role in genome replication, asymmetric dimerization of THOVPol may also play a functional role in RNA transcription.*~~"

As a result, the paragraph in the "Asymmetric THOVPol dimer and RNA synthesis" section (Line 782-789 of track-change MS) is revised.

6) Another large part of their story is that the Thogoto replicase (asymmetric dimer) is independent of host factor ANP32.
Here they present some functional assays in Figure 6. However they

perform these assays in human cells that lack human ANP32A and ANP32B. Others have recently shown that where the interaction between influenza polymerase symmetric dimers is less strong, other ANP32 family members such as ANP32E can be used instead. At the very least the authors should perform these assays in triple knockout cells.

Response:

We thank the reviewer's insightful comments. We have cited the latest results regarding the usage of ANP32E (Sheppard *et al*, 2023; Zhang *et al.*, 2020). We have conducted additional assays in the ANP32A/B/E triple knockout 293T cells. As illustrated in the revised **Fig.6i**, it is evident that human ANP32E proteins have limited enhancement (~5x) of THOVPol activity. We have conducted additional experiments to test effects of mouse and tick ANP32 proteins on THOVPol activity, the strongest effect was found with mouse ANP32A which increases THOVPol activity by 10-times. Because these effects are much smaller than the effect of ANP32 proteins on FluPol (up to 5000x enhancement), we now conclude that THOVPol activity is weakly dependent on ANP32. The "Abstract", "Asymmetric THOVPol dimer and RNA synthesis" and "Discussion" sections have been modified to describe a weak ANP32 protein dependency for THOVPol activity.

7) In addition since thogoto virus is not a natural virus of humans, the authors should also assessing whether the addition of ANP32 proteins cloned from different more relevant species such as ticks or mice might further enhance the replication activity they observe- the small but significant enhancement they see in presence of human ANP32B might be much greater if a better species matched host protein were used.

Response:

We thank the reviewer for the suggested experiment. Accordingly, we performed complementation experiments in TKO cells (ANP32A/B/E triple knock-out human 293T cells) with ANP32 proteins of mouse and the *Rhipicephalus sanguineus* tick (brown dog tick, from which THOV has been isolated (Lledo *et al*, 2020)), the results are shown in the revised **Fig.6i**. The results show that in the TKO cells, human ANP32A, B and mouse ANP32B can greatly increase IAV Pol activity by ~ 5000x. In contrast, none of the ANP32 proteins (human ANP32A/B/E, mouse ANP32A/B/E and tick ANP32A/B) are able to greatly increase THOVPol activity. A maximum of ~10x activity increases were observed when mouse ANP32A protein were complemented. Based on these results we claim that there is a weak ANP32 protein dependency for THOVPol activity.

8) It would also be useful to show the raw data so that the absolute levels of polymerase activity can be compared in these cells between Thogoto virus and IAV polymerase.

Response:

We thank the reviewer for this suggestion. We have now included the raw data as an excel file for THOV and IAV polymerase activities in TKO cells in **Source data**.

9) In summary the structural data here are very interesting but it is not clear whether the dimeric forms of polymerase described are biologically relevant in the way the authors suggest and the biological validation is currently lacking. To have such a controversial piece of work accepted in the field the authors will need to develop better biological assays to validate their findings.

Response:

We thank the reviewer for the constructive suggestions and we hope our revisions have addressed the reviewer's concerns.

References

Sheppard, C.M., Goldhill, D.H., Swann, O.C., Staller, E., Penn, R., Platt, O.K., Sukhova, K., Baillon, L., Frise, R., Peacock, T.P., Fodor, E., Barclay, W.S. (2023) An Influenza A virus can evolve to use human ANP32E through altering polymerase dimerization. ***Nat Commun*** 14: 6135

Zhu, Z., Fan, H., Fodor, E. (2023) Defining the minimal components of the influenza A virus replication machinery via an in vitro reconstitution system. ***PLoS Biol*** 21: e3002370

Zhang, Z., Zhang, H., Xu, L., Guo, X., Wang, W., Ji, Y., Lin, C., Wang, Y., Wang, X. (2020) Selective usage of ANP32 proteins by influenza B virus polymerase: Implications in determination of host range. ***PLoS Pathog*** 16: e1008989

Carrique, L., Fan, H., Walker, A.P., Keown, J.R., Sharps, J., Staller, E., Barclay, W.S., Fodor, E., Grimes, J.M. (2020) Host ANP32A mediates the assembly of the influenza virus replicase. ***Nature*** 587: 638-643

Wandzik, J.M., Kouba, T., Karuppasamy, M., Pflug, A., Drncova, P., Provaznik, J., Azevedo, N., Cusack, S. (2020) A Structure-Based Model for the Complete Transcription Cycle of Influenza Polymerase. ***Cell*** 181: 877-893 e21

Lledo, L., Gimenez-Pardo, C., Gegundez, M.I. (2020) Epidemiological Study of Thogoto and Dhori Virus Infection in People Bitten by Ticks, and in Sheep, in an Area of Northern Spain. ***Int J Environ Res Public Health*** 17

Hao, S., Ning, K., Wang, X., Wang, J., Cheng, F., Ganaie, S.S., Tavis, J.E., Qiu, J. (2020) Establishment of a Replicon Reporter of the Emerging Tick-Borne Bourbon Virus and Use It for Evaluation of Antivirals. ***Front Microbiol*** 11: 572631

Peng, Q., Liu, Y., Peng, R., Wang, M., Yang, W., Song, H., Chen, Y., Liu, S., Han, M., Zhang, X., Wang, P., Yan, J., Zhang, B., Qi, J., Deng, T., Gao, G.F., Shi, Y. (2019) Structural insight into RNA synthesis by influenza D polymerase. ***Nat Microbiol*** 4: 1750-1759

Kouba, T., Drncova, P., Cusack, S. (2019) Structural snapshots of actively transcribing influenza polymerase. ***Nat Struct Mol Biol*** 26: 460-470

Zivanov, J., Nakane, T., Forsberg, B.O., Kimanius, D., Hagen, W.J., Lindahl, E., Scheres, S.H. (2018) New tools for automated high-resolution cryo-EM structure determination in RELION-3. ***eLife*** 7
Koppstein, D., Ashour, J., Bartel, D.P. (2015) Sequencing the cap-snatching repertoire of H1N1 influenza provides insight into the mechanism of viral transcription initiation. ***Nucleic Acids Res*** 43: 5052-64
Guilligay, D., Kadlec, J., Crepin, T., Lunardi, T., Bouvier, D., Kochs, G., Ruigrok, R.W., Cusack, S. (2014) Comparative structural and functional analysis of orthomyxovirus polymerase cap-snatching domains. ***PLoS One*** 9: e84973
Ozawa, M., Shimojima, M., Goto, H., Watanabe, S., Hatta, Y., Kiso, M., Furuta, Y., Horimoto, T., Peters, N.R., Hoffmann, F.M., Kawaoka, Y. (2013) A cell-based screening system for influenza A viral RNA transcription/replication inhibitors. ***Sci Rep*** 3: 1106
Olson, A.C., Rosenblum, E., Kuchta, R.D. (2010) Regulation of influenza RNA polymerase activity and the switch between replication and transcription by the concentrations of the vRNA 5' end, the cap source, and the polymerase. ***Biochemistry*** 49: 10208-15
Leahy, M.B., Dessens, J.T., Nuttall, P.A. (1997) In vitro polymerase activity of Thogoto virus: evidence for a unique cap-snatching mechanism in a tick-borne orthomyxovirus. ***J Virol*** 71: 8347-51
Albo, C., Martín, J., Portela, A. (1996) The 5' ends of Thogoto virus (Orthomyxoviridae) mRNAs are homogeneous in both length and sequence. ***J Virol*** 70: 9013-7

REVIEWERS' COMMENTS

Reviewer #1 (Remarks to the Author):

This revised submission has successfully addressed the concerns in the previous review.

Reviewer #2 (Remarks to the Author):

The authors have substantially revised the initial manuscript, including optimizing the method part especially the cryo-EM data processing, reorganizing and improving the text and figures. Results from this study are now well indicated and presented by the figures.
Overall, this work is of significance to the RNA virus field and a broader virology community in terms of molecular mechanisms of viral RNA transcription and replication and host infection. Particularly, the authors revealed the reason why THOVPol can not bind ANP32A with the same interaction mode as in the FluCPol. It is also enlightening that the authors obtained an asymmetric polymerase dimer with RNA product bound and described the structural details by collecting multiple large cryo-EM datasets. I don’t have any objection to its acceptance for publishment. To improve the manuscript, I suggest making the protein sequence alignment figures more colorful to help readers more easily find out the specific residues.

Reviewer #3 (Remarks to the Author):

I appreciate the thorough was in which the authors have addressed my comments. They have ammended their text to be more cautious in the claims about the biological experiments.

Remaining reviewer comments:
Reviewer #1:
This revised submission has successfully addressed the concerns in the previous review.

Response:
We thank the reviewer’s constructive comments which helped us to improve the manuscript.

Reviewer #2:
The authors have substantially revised the initial manuscript, including optimizing the method part especially the cryo-EM data processing, reorganizing and improving the text and figures. Results from this study are now well indicated and presented by the figures. Overall, this work is of significance to the RNA virus field and a broader virology community in terms of molecular mechanisms of viral RNA transcription and replication and host infection. Particularly, the authors revealed the reason why THOVPol can not bind ANP32A with the same interaction mode as in the FluCPol. It is also enlightening that the authors obtained an asymmetric polymerase dimer with RNA product bound and described the structural details by collecting multiple large cryo-EM datasets. I don’t have any objection to its acceptance for publishment. To improve the manuscript, I suggest making the protein sequence alignment figures more colorful to help readers more easily find out the specific residues.

Response:
We thank the reviewer’s constructive comments which helped us to improve the manuscript. We again thank the reviewer’s suggestion. We updated the protein sequence alignment figures (Supplementary Fig. 6) by adding color boxes to highlight specific residues, thereby aiding readers in quickly identifying them.

Reviewer #3:
I appreciate the thorough was in which the authors have addressed my comments. They have ammended their text to be more cautious in the claims about the biological experiments.

Response:
We thank the reviewer’s suggested experiments and constructive comments which helped us to improve the manuscript.
